# Solution Spectroscopic Analysis of Ligand Exchange in Uranium Mono-Oxo Complexes

**DOI:** 10.1021/acs.inorgchem.6c03607

**Published:** 2026-10-06

**Authors:** Florian Ressnik, Tyler-Rayne Nero, Maximilian G. Bernbeck, Johannes Leisen, Bess Vlaisavljevich, Julie E. Niklas, Henry S. La Pierre

**Affiliations:** † School of Chemistry & Biochemistry, 1372 Georgia Institute of Technology, Atlanta, Georgia 30332, United States; ‡ Nuclear and Radiological Engineering and Medical Physics Program in the School of Mechanical Engineering, Georgia Institute of Technology, Atlanta, Georgia 30332, United States; § Physical Sciences Division, Pacific Northwest National Laboratory, Richland, Washington 99352, United States; ∥ Department of Chemistry, The University of Iowa, Iowa City, Iowa 52242, United States

## Abstract

Four uranium mono-oxo imidophosphorane complexes with a closed set of systematically changed substituents (pyrrolidine vs piperidine and introduction of one *tert*-butyl group) are presented. All complexes exhibit an inverse *trans*-influence (ITI). The rapid exchange of axial and equatorial ligands at room temperature, the unique ^31^P nuclear magnetic resonance (NMR) handle of the ligands, and the complexes’ overall closed-shell singlet ground state facilitate the determination of ligand-exchange barriers via variable-temperature (VT) and exchange spectroscopy (EXSY) NMR. Computational analysis delineates electronic and steric contributions to the experimentally determined exchange barriers.

## Graphical abstract

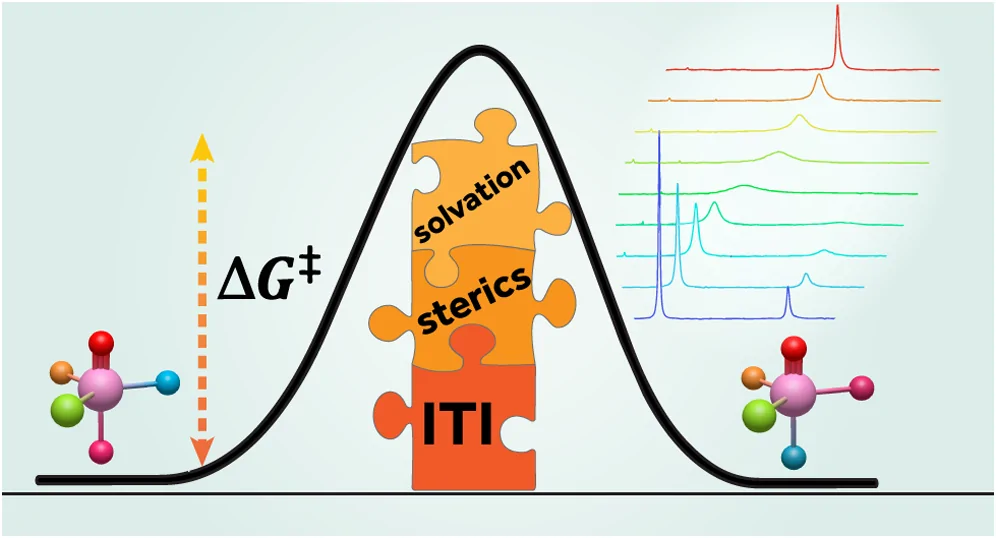

## Introduction

The inverse *trans*-influence (ITI) is a unique structural phenomenon of the *f*-elements, most prominently observed in systems with metal–ligand multiple bonds (in high-oxidation-state early- and mid-actinide complexes.^1–3^ In the ITI, the group *trans* to strongly σ- or π-donating ligands are more strongly bound (mutually reinforcing each other) to the metal and exhibit shorter metal–ligand bond lengths.^1–3^ This phenomenon contrasts with the more widely observed *trans*-influence in transition-metal complexes, where bonds *trans*- to strong σ-donating ligands are weakened and lengthened.^4^ More specifically, in dioxo complexes, transition-metal species favor the *cis*-orientation,^5^ whereas actinyl species exclusively exist in the *trans*- configuration.^2,6^ While limited to a handful of elements, it drives the bonding of the dominant chemical form of uranium – uranyl, [UO_2_]^2+^.^1,6–8^ Therefore, determining the thermodynamics and physical basis of this phenomenon is critical to understanding the structure and environmental mobility of the uranyl ion, leading to opportunities for selective separations and divergent chemical reactivity in the actinides.

While computational studies of the ITI have been employed to rationalize the observed bonding and structural features in lanthanide and actinide complexes,^1,2,7,9–18^ experimental quantification has been limited to the solid state.^19–25^ Comparison of equivalent ligand types in both the equatorial and axial (the *trans*-) positions in a given complex can reveal differences in bond lengths. The ITI% is defined as the ratio of the axial to equatorial metal–ligand bond length to quantify the observed shortening of the former bond with respect to the latter.^12^ The smaller the ITI%, the stronger the observed ITI. However, a limitation of this analysis is that it requires the same ligands to be present in the axial and equatorial positions. While structural features and distortions in the solid state can imply the thermodynamic effect of the ITI, there are relatively few complexes that meet this requirement. The earliest structural evidence in which the ITI can be observed is in [NEt_4_]_2_[PaOCl_5_] and its uranium structural analogue [PPh_4_][UOCl_5_].^19,20^ Other canonical examples include complexes with uranyl, uranium mono-oxo, and mono-imido groups.^26–46^

Determining an ITI% relies on high-precision bond length data and is therefore limited to solid-state crystallographic methods and theoretical calculations. This analysis will rely heavily on modeling results, which present their own challenges, if such metrics cannot be extracted from single-crystal X-ray diffraction (SC-XRD) data, either due to difficulties in crystallization or disorder. Additionally, some complexes in which the ITI is demonstrated to drive structural features (by combining spectroscopy and modeling approaches) do not provide a crystallographically quantifiable ITI.^46^ Ideally, solution spectroscopic methods could be developed to quantify the ITI to circumvent crystallographic issues, provide benchmarks for modeling efforts, and give insight into the thermodynamics of this phenomenon in solution. In this study, we investigate intramolecular ligand-exchange barriers with dynamic nuclear magnetic resonance (NMR) studies as a spectroscopic probe of the kinetics and thermodynamics of structural rearrangement in actinide mono-oxo complexes.

Recently reported uranium mono-oxo complexes (**UO-(NP** ^**pip**^ **)**_**4**_, **UO-(NPC** ^**1**^ **)**_**4**_, and **UO-(NPC^1‑pip^)**_**4**_) and a new **UO-(NP** ^**pyrr**^ **)**_**4**_ derivative are ideal candidates for this solution-based ITI study (Figure 1).^21,24^ These trigonal-bipyramidal complexes each feature four identical imidophosphorane ligands, where one is axial (*trans* to the oxo ligand) and three are in the equatorial plane. This geometry allows for direct comparison of axial and equatorial environments and consequently provides an opportunity to probe how ligand steric effects and donor properties govern ligand-exchange dynamics in solution, since these complexes can be probed with ^31^P­{^1^H}-NMR. The complexes are also closed-shell, allowing for density functional theory (DFT) modeling of the NMR spectra, leading to a more direct comparison between idealized modeling environments and solution measurements.

**Figure 1:**
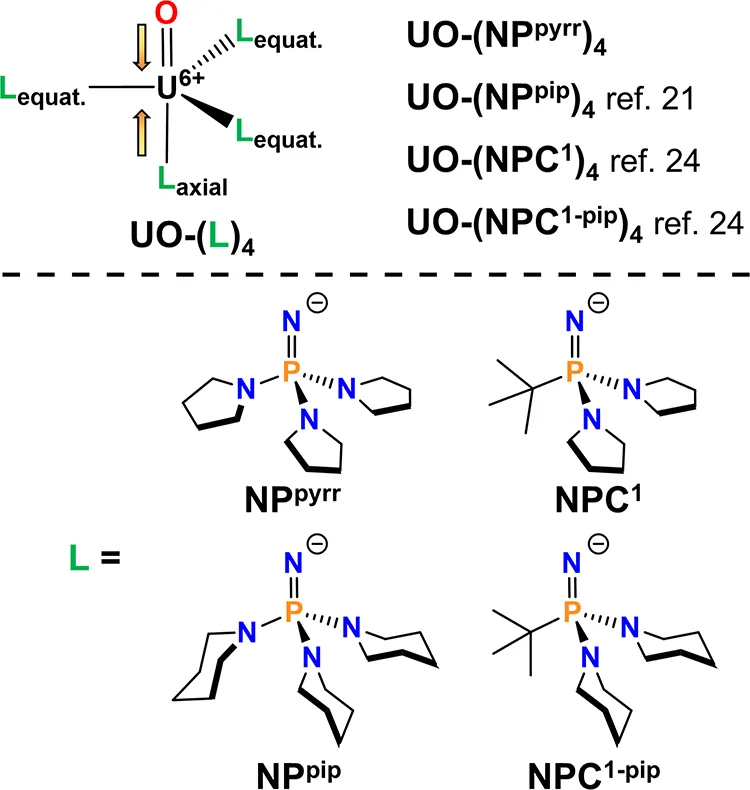
Overview of uranium mono-oxo complexes used in this study.

## Results

The uranium mono-oxo complexes **UO-(NP** ^**pip**^ **)**_**4**_, **UO-(NPC^1‑pip^)**_**4**_, and **UO-(NPC** ^**1**^ **)**_**4**_ were prepared as previously described by oxidation of their tetravalent precursor with N_2_O or 2,3:5,6-dibenzo-7-azabicyclo[2.2.1]­hepta-2,5-diene (dbabhNO).^21,24^ **UO-(NP** ^**pyrr**^ **)**_**4**_ was prepared for this study by analogous methods (see Experimental Section). SC-XRD analysis has been performed on all four compounds.^21,24^ It should be noted that we have previously demonstrated that significant crystal engineering is required to crystallize these complexes in low-symmetry space groups and with limited disorder.^24^ **UO-(NPC** ^**1**^ **)**_**4**_ was identified through these prior studies as the only case to yield a high-quality structure. The three other complexes included in this spectroscopic study crystallize in high-symmetry space groups with extensive static whole-molecule disorder and dynamic disorder of piperidine and pyrrolidine groups. Connectivity can be assigned from the crystal structures, and in all cases, the axial U–N bonds are qualitatively shorter than the equatorial bonds (Table S5), as expected based on the structure of **UO-(NPC** ^**1**^ **)**_**4**_. These connectivities do indicate an ITI in all cases; however, precise description of the ITI% metric is limited to **UO-(NPC** ^**1**^ **)**_**4**_ (ITI% 93.8) due to limitations of data quality for **UO-(NP** ^**pyrr**^ **)**_**4**_, **UO-(NP** ^**pip**^ **)**_**4**_, and **UO-(NPC^1‑pip^)**_**4**_. The three samples were chosen, despite their intrinsic crystallographic disorder, in order to examine the solution dynamics across a series of electronically and sterically modulated complexes.

These mono-oxo complexes were probed by variable-temperature (VT) NMR to examine their solution dynamics. The complexes each exhibit a single resonance in the ^31^P­{^1^H}-NMR spectra in THF-*d*_8_ at room temperature (Figure 2, top for **UO-(NP** ^**pyrr**^ **)**_**4**_), which is in contrast with the expectation of two signals in a 3:1 ratio for three equatorial and one axial ligand based on their solid-state structures. Upon cooling to −95 °C (Figure 2, bottom), two resonances in a 3:1 ratio reflecting the equatorially and axially coordinated NP ligands, respectively, are observed. The assignment of the two signals is supported by DFT results (*vide infra*). This spectroscopic feature can be attributed to fast ligand exchange that cannot be resolved on the NMR time scale at room temperature. The same is observed for the other complexes, but the splitting occurs at different temperatures (coalescence temperature, *T*_c_), specifically at −71 °C for **UO-(NP** ^**pyrr**^ **)**_**4**_, −56 °C for **UO-(NP** ^**pip**^ **)**_**4**_, −47 °C for **UO-(NPC** ^**1**^ **)**_**4**_, and −41 °C for **UO-(NPC^1‑pip^)**_**4**_ (Figures S31–S38).

**Figure 2:**
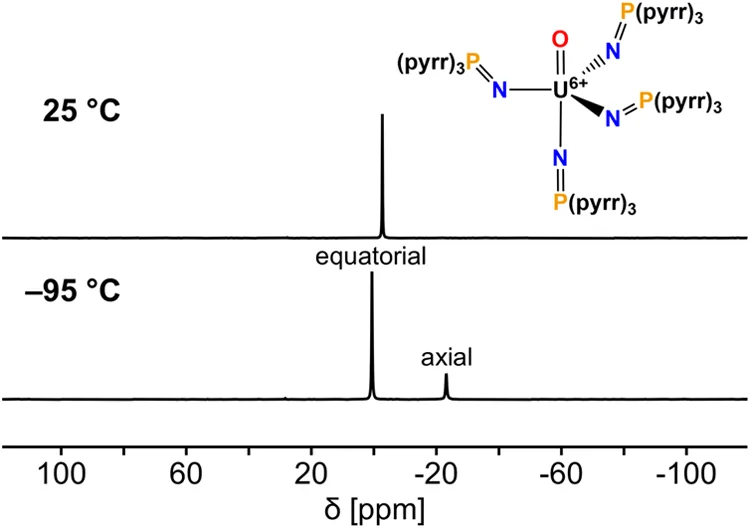
^31^P­{^1^H}-NMR spectra of **UO-(NP** ^**pyrr**^ **)**_**4**_ in THF-*d*_8_ at 25 °C (top) and −95 °C (bottom).

While a ligand rearrangement is evident, the molecular-level details of such an exchange cannot be assigned from the NMR measurement alone. Polytopal rearrangements in pentacoordinate trigonal-bipyramidal complexes have been proposed to proceed via a range of related mechanisms.^47,48^ While the exact mechanism is unknown for these complexes, the rearrangement must involve a transition state, which does not retain axial coordination of the oxo and imidophosphorane ligands. Thus, the energetic preference of the inverse *trans*-influence is lost and contributes to the height of the exchange barrier (Δ*G* ^‡^). Additionally, the barrier is impacted by ligand steric effects, solvation, and solvent viscosity.^49^ Parsing these relative contributions to the barrier, even computationally, is difficult because of the many conformational degrees of freedom that are energetically accessible under experimental conditions. Note that the steric contributions, and in turn conformational flexibility, vary across the complexes and can be quantified by a geometric parameter referred to as the *G*-parameter (Table S27).^50^

A reasonable first assumption is that the exchange process occurs via a square-pyramidal intermediate or transition state. The energy landscape of this simplified ligand-exchange process can be visualized as depicted in Figure 3. While elucidating structural details is challenging, Δ*G* ^‡^ can be extracted from variable-temperature NMR data by measuring the coalescence temperature (*T*_c_) and maximum peak separation (δ_ν_) of the exchanging signals in the “no exchange limit” (at low temperatures). The relation between these parameters and Δ*G* ^‡^ for a system of exchange between unequal populated sites was described by Shanan-Atidi and Bar-Eli.^51^ Equation 1 is a variation of the original published equation, where *R* is the ideal gas constant, *k* is the Boltzmann constant, *h* is Planck’s constant, and Δ*P* corresponds to the difference in the relative concentration of the two exchanging sites (for a 3:1 ratio, Δ*P* = 0.75–0.25 = 0.5). The values for *X* are dependent on Δ*P* and are tabulated by Egan.^52^ For Δ*P* = 0.5, *X* = 2.3008.

1 $$\Delta G^{‡}=RT_{c}\ln\left[\frac{kT_{c}}{h\pi \delta_{\nu }}\left(\frac{X}{1+\Delta P}\right)\right]$$

Originally, this equation was derived for exchange between two energetically different molecules. The population difference of the two species in equilibrium then directly translates to an energetic bias for one over the other, and two different exchange barriers (back-and-forth exchange) can be calculated. For the uranium mono-oxo complexes, the integration for the two resonances observed at low temperature is fixed at 3:1 for the number of equatorial and axial ligands. Due to the nature of this polytopal rearrangement, the molecule is invariant before and after exchange (the reactant and product are equal in energy, as shown in Figure 3). Consequently, defining two exchange barriers is not reasonable. The ligand-exchange process in uranium mono-oxo complexes consists of a simultaneous exchange of an axial to equatorial ligand and vice versa. The concentration of axial ligands equals the concentration of uranium mono-oxo complexes, and consequently, the formal rate of exchange of the axial ligand is identical with the overall exchange process of the molecule. Equation 1 represents this overall exchange process.

**Figure 3:**
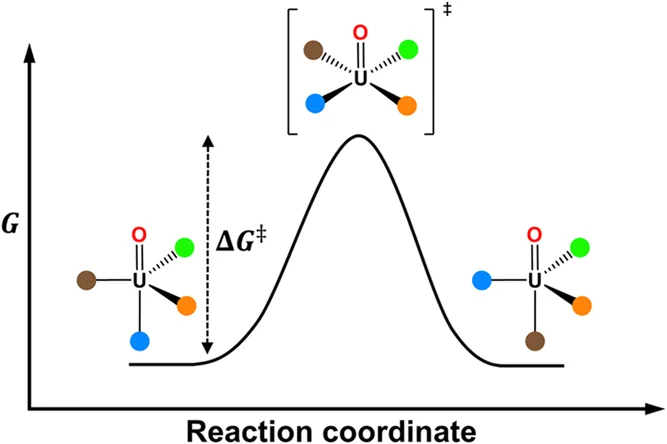
Schematic energy diagram for a ligand-exchange process in uranium mono-oxo complexes via a square-pyramidal transition state. Each colored dot represents an imidophosphorane ligand.

Variable-temperature ^31^P­{^1^H}-NMR experiments for the four complexes **UO-(NP** ^**pyrr**^ **)**_**4**_, **UO-(NP** ^**pip**^ **)**_**4**_, **UO-(NPC** ^**1**^ **)**_**4**_, and **UO-(NPC^1‑pip^)**_**4**_ were conducted to obtain the respective coalescence temperature and δ_ν_ value (Figures 4 and S31–S38). Using eq 1, exchange barriers for the complexes were calculated (Table 1). Qualitative VT-NMR data of **UO-(NP** ^**pip**^ **)**_**4**_ had been previously reported but was recollected for this study with consistent temperature calibration for all four samples.^21^ Computational studies included as the Supporting Information for **UO-(NP** ^**pip**^ **)**_**4**_ suggest that at least one intermediate exists in an energy range consistent with experimental barriers (Figure S71); however, a thorough study of the exchange mechanism was not undertaken.

**Figure 4:**
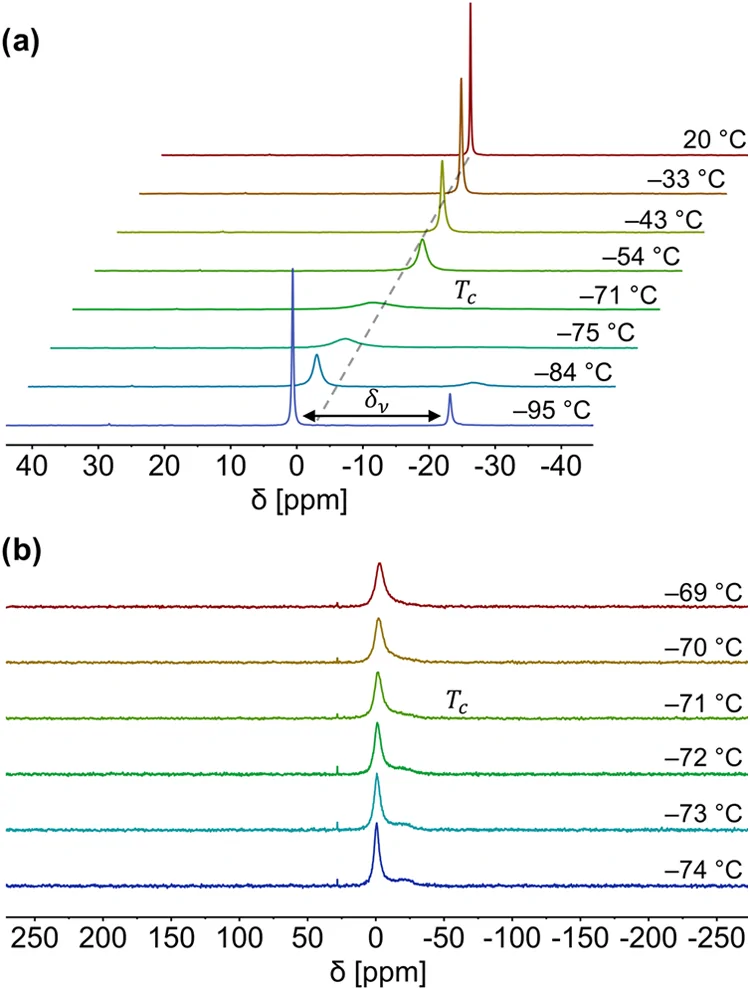
(a) Stack plot (along 30° vector) of VT-^31^P­{^1^H}-NMR spectra of **UO-(NP** ^**pyrr**^ **)**_**4**_ between −95 and 20 °C in THF-*d*_8_. (b) Stack plot of VT-^31^P­{^1^H}-NMR spectra of **UO-(NP** ^**pyrr**^ **)**_**4**_ between −74 and −69 °C in THF-*d*_8_.

**Table 1:** Summary of *T*_c_, δ_ν_, and Δ*G* ^‡^ (See eq 1) of **UO-(NP^pyrr^)_4_**, **UO-(NP^pip^)_4_**, **UO-(NPC^1^)_4_**, and **UO-(NPC^1‑pip^)_4_** in THF-*d*_8_ Based on VT-^31^P­{^1^H}-NMR Experiments^a^

	*T*_c_ [°C]	δ_ν_ [Hz]	Δ*G* ^‡^ [kcal/mol]
**UO-(NP^pyrr^)_4_**	–71(2)	4815(1)	7.98(8)
**UO-(NP^pip^)_4_**	–56(2)	5694(1)	8.53(8)
**UO-(NPC^1^)_4_**	–47(2)	5704(1)	8.90(8)
**UO-(NPC^1‑pip^)_4_**	–41(2)	5710(1)	9.15(8)

a Errors on Δ*G* ^‡^ are calculated by propagating the errors on *T*_c_ and δ_ν_ estimated from the data.

Note that there are uncertainties in the exchange barriers obtained via the VT-NMR method. The coalescence temperature can only be measured to ±2 K accuracy, and the measured peak separation δ_ν_ in the no-exchange regime is limited by the solvent freezing point (−108.5 °C) and accessible instrument probe temperatures.

Other methods, such as exchange spectroscopy (EXSY) NMR, facilitate complementary insight (Figure 5). This 2D experiment shows correlations between signals that exchange with each other. The ^31^P–^31^P-EXSY-NMR spectrum of **UO-(NP** ^**pyrr**^ **)**_**4**_ at −95 °C in THF-*d*_8_ is reported in Figure 5a. The spectrum shown along each dimension is comparable to that given in Figure 2. The two signals correspond to the phosphorus atoms in the equatorial (0.6 ppm) and axial ligands (−23.2 ppm) in **UO-(NP** ^**pyrr**^ **)**_**4**_. The blue correlation signals can be distinguished into diagonal (highlighted with blue ellipses) and cross peaks (highlighted with red ellipses) (Figure 5a). Cross peaks only occur when there is exchange. The intensity of the correlation signals is dependent on the mixing time, τ_m_, and the exchange rate constant, *k*. This relation is described by equations from Jeener et al. (details in Supporting Information).^53^ Several ^31^P–^31^P-EXSY-NMR spectra of **UO-(NP** ^**pyrr**^ **)**_**4**_ were collected with different mixing times, and integrated signal intensities are listed in Table S3. The data is normalized by forming the cross-to-diagonal peak intensity ratio *I*_c_/*I*_d_ (see Supporting Information). Additionally, the buildup curve for the cross peak-to-diagonal peak intensity ratio (*I*_c_/*I*_d_) with increasing mixing time is given in Figure 5b. This ratio can be described by the equations from Jeener et al.^53^ For a 3:1 ratio (χ_A_ = 0.75, χ_B_ = 0.25), neglecting dipolar cross relaxation, and using the assumption that the *T*_1_ relaxation for both signals is identical, the equations simplify to the following expression

2 $$\frac{I_{c}}{I_{d}}=\frac{I_{BA}+I_{BA}}{I_{AA}+I_{BB}}=\frac{3\;\sinh\left(\frac{1}{2}k\tau_{m}\right)}{4\;\cosh\left(\frac{1}{2}k\tau_{m}\right)+\sinh\left(\frac{1}{2}k\tau_{m}\right)}$$

Only the initial curve (prior to reaching a plateau) is affected by the exchange rate constant, *k*. For the ligand exchange in **UO-(NP** ^**pyrr**^ **)**_**4**_, *k* can be extracted by fitting the *I*_c_/*I*_d_ data points with eq 2. The fitted *k* value must be corrected for the respective ratio. For the uranium mono-oxo complexes, the following expression can be used

3 $$k_{ax\to eq}=\frac{k}{0.25}$$

With a variant of the Eyring equation, the rate constant is used to calculate the exchange barrier Δ*G* ^‡^.

4 $${\Delta G}^{‡}=\mathrm{RT}\ln\left[\frac{k_{b}T}{hk_{ax\to eq}}\right]$$

The results are summarized and compared with the VT-NMR method in Table 2. The ligand-exchange barrier obtained with the EXSY-NMR method is in good agreement with that of the VT-NMR method, validating the results of the latter. Importantly, VT-NMR details the temperature dependence of the process, and EXSY-NMR determines the kinetics of this process at a given temperature. Both methods provide equivalent values for Δ*G* ^‡^ and support the hypothesis that this exchange proceeds through a one-step mechanism. Since VT-NMR is much more time-efficient, it was employed across all four complexes after EXSY was established to give consistent values for **UO-(NP** ^**pyrr**^ **)**_**4**_.

**Figure 5:**
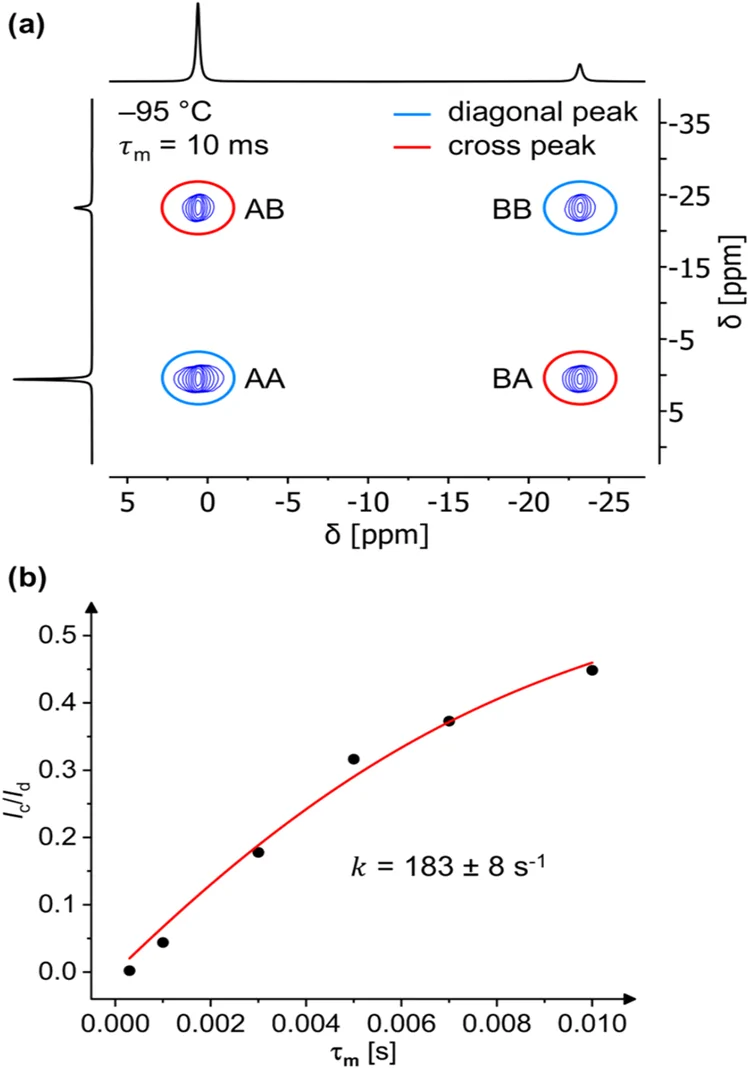
(a) ^31^P–^31^P-EXSY-NMR spectrum of **UO-(NP** ^**pyrr**^ **)**_**4**_ in THF-*d*_8_ at −95 °C with a mixing time of 10 ms. Blue ellipses highlight diagonal (AA and BB) and red ellipses highlight cross peaks (AB and BA). (b) *I*_c_/*I*_d_ vs mixing time of ^31^P–^31^P-EXSY-NMR spectra (THF-*d*_8_, −95 °C) of **UO-(NP** ^**pyrr**^ **)**_**4**_ with fitting curve.

**Table 2:** Comparison of Exchange Barriers of **UO-(NPpyrr)_4_** Derived from VT- and EXSY-NMR Methods

	*k*_ax→eq_ [s^–1^]	*T* [K]	Δ*G* ^‡^ [kcal/mol]
**UO-(NP^pyrr^)_4_** VT method	9862	202(2)	7.98(8)
**UO-(NP^pyrr^)_4_** EXSY method	732	178(1)	7.90(5)

Given that high-resolution XRD structural data are not available for three of the complexes, DFT calculations are employed to explore the role of the ITI as a function of ligand substitution. First, DFT geometry optimizations were performed for the four complexes with the PBE, TPSS, and MN15-L functionals (see computational details below). For the high-quality experimental structure of **UO-(NPC** ^**1**^ **)**_**4**_, the bond distances obtained by DFT are in excellent agreement with the experiment for all three functionals (maximum differences of 0.06 Å across the U=O and U–N bonds) (Table S5). However, the U–N_axial_–P angle deviates by 22.2° from experiment using MN15-L, while the structures obtained with PBE and TPSS reproduce the angle well with differences from experiment of only 1.8 and 1.6°, respectively (Table S6). Larger differences between DFT and experiment are obtained for the three other complexes, as is expected due to the extensive disorder in the solid state. That said, the differences between the various DFT methods are similar for all four complexes, suggesting similar functional dependence across the systems. The structures optimized with the TPSS functional were used in the following analyses.

In addition to structural changes, the presence of an ITI is also supported by DFT bonding analyses. Taking **UO-(NP** ^**pyrr**^ **)**_**4**_ as a representative example, the U–N_axial_ bond distance is 0.100 Å shorter than the average of the three U–N_equat_ bond distances (Table 3). Likewise, the Mayer and Nalewalski–Mrozek Type 3 (NM-3) bond orders have a marked increase of 0.176 and 0.244, respectively. Differences of a similar magnitude are obtained for the other three complexes as well (Table S7). Similar conclusions are obtained via the quantum theory of atoms in molecules (QTAIM), in which the ratio of the kinetic energy density to the total energy density, |*V*(*r*)|/*G*(*r*), indicates that the U–N_axial_ bonds are less polarized than the U–N_equat_ bonds (Table S24).

**Table 3:** Selected Bond Distances and Bond Orders (Mayer and Nalewalski–Mrozek Type 3, NM-3) Are Reported for **UO-(NPpyrr)_4_** ^a^

bond	distance	mayer	NM-3
U=O	1.851 Å	1.922	2.620
U–N_axial_	2.096 Å	1.132	1.783
U–N_equat_ (mean)	2.196(9) Å	0.956(19)	1.539(12)

a Standard deviation is reported for mean values.

These bonding metrics can be further understood by inspection of the natural localized molecular orbitals (NLMOs) (Figures S62–S65). While the U**–**N interaction for each ligand involves both σ and π contributions, the differences between the equatorial and axial ligands due to the ITI emerge in the σ bonding (Figure 6). The total percent contribution of uranium is larger by 6.0–9.2% (depending on the ligand) in the axial bonds than in equatorial bonds (Table S18). Moreover, the *f*-orbitals have a larger contribution than the *d*-orbitals to the σ bond in the axial position, while the opposite is true for the equatorial σ bond. A correlation between an increase in *f*-orbital contribution and a stronger ITI has been proposed to arise from increased mixing from the 6*p* semicore orbitals.^2^ Since no significant 6*p* mixing is observed in the NLMOs, single-point calculations were performed including the 6*p* orbitals in the frozen core, which has been used to quantify the stabilization from core polarization.^12,54^ A minimal impact on the total energy of the complex (ca. 17 kJ/mol) and the energies of the valence orbitals was obtained (<0.02 eV). This suggests that while the delocalized bonding in the ITI leads to an energetic stabilization in the axial position, the driving force in these complexes is not due to increased 6*p* orbital mixing. On the other hand, the aforementioned differences in bond distances, bond orders, NLMO orbital contributions, and atomic charges on the nitrogen atoms (Table S8) are consistent with the *cis*-destabilization mechanism.^12,39,55^

**Figure 6:**
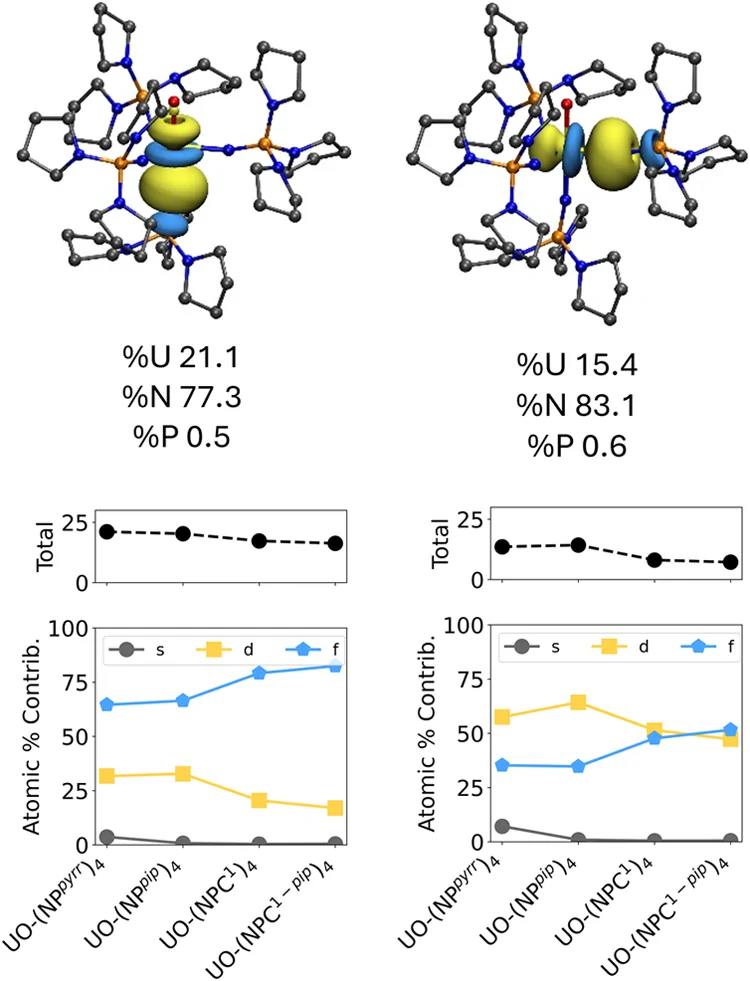
Natural localized molecular orbitals (NLMOs) for the σ bonds in U–N_axial_ (left) and U–N_equat_ (right). Top: NLMOs for **UO-(NP^pyrr^)_4_** with the percent total U, N, and P contributions. Bottom: The atomic orbital decomposition for all four complexes. The total uranium contribution is in black, and its decomposition into atomic contributions is in color.

Moreover, the increased orbital mixing in the ITI does indeed result in the strengthening of the metal–ligand bond in the axial position for all four complexes. Specifically, energy decomposition analysis (EDA) was performed by dividing the molecule into two fragments, one containing a single monoanionic ligand and the other consisting of the remainder of the molecule, which is a monocation. From the calculations on the fragments, the interaction energy (*E*_int_), described by eq 5

5 $$E_{\mathrm{int}}=E_{\mathrm{Pauli}}+E_{\mathrm{Elec}}+E_{\mathrm{Orb}}+E_{\mathrm{Disp}}$$

is computed and subsequently decomposed into the contributions from Pauli repulsion (*E*_Pauli_), electrostatic (*E*_Elec_), orbitalic (*E*_Orb_), and dispersion interactions (*E*_Disp_). These computations are performed for both the axial and equatorial ligands. The trends in **UO-(NP** ^**pyrr**^ **)**_**4**_ are given in Table 4 and are representative of the results for the other complexes (Tables S21 and S22). The interaction energy in the axial position for **UO-(NP** ^**pyrr**^ **)**_**4**_ is −180.9 kcal/mol, which is 13.3 kcal/mol stronger than that in the equatorial position at −167.6 kcal/mol (Table 4), supporting the presence of an ITI. Dispersion contributes a similar amount at 5.6 and 5.3% for the axial and equatorial ligands, respectively. The percent orbital contribution to this interaction is 39.1% in the axial position for all four ligands, falling to 35.1% in the equatorial position. This change can be quantified via the extended transition-state natural orbitals for chemical valence (ETS-NOCV) approach. The pair orbital interaction energies show a larger and more stabilizing interaction of −59.4 kcal/mol associated with the axial ligand, where the analogous interaction for an equatorial ligand is −35.1 kcal/mol (Table S23). The associated deformation density shows that this corresponds to the σ donation in the axial ligand, which is weaker by ca. −25 kcal/mol than that in the equatorial position (Figures S67–S70). Moreover, the number of electrons transferred (or the NOCV eigenvalue for each pair) is larger for the axial ligand at 0.75 but falls to 0.64 in the equatorial position.

**Table 4:** Energy Decomposition Analysis Results for **UO-(NPpyrr)_4_** for the Axial Ligand and Equatorial Ligand^a^

	*E*_int_	%Elect	%Orb	%Disp
axial	–180.9	55.3	39.1	5.6
equatorial	–167.7	59.6	35.1	5.3

a Interaction energy (*E*_int_) in kcal/mol. The percent electrostatic, orbitalic, and dispersion interactions constitute the total attractive energy.

Finally, the ^31^P-NMR chemical shifts can be calculated for comparison with the measured values. DFT spectra show two peaks, one associated with each ligand, axial and equatorial (Figure 7). When the H_3_PO_4_ acid reference (computed by DFT directly) is used for all four complexes, both peaks shift to more positive values across the four complexes, following the experimental trend. Once again, taking **UO-(NP** ^**pyrr**^ **)**_**4**_ as a representative example, the chemical shifts from experiment are −23.2 and +0.6 ppm, assigned to the axial and equatorial positions, respectively. This assignment is supported by DFT, where both the PBE and PBE0 functionals qualitatively agree with the assignment; however, the chemical shifts are in qualitative but not quantitative agreement with experiment (Tables S11 and S12). Achieving quantitative agreement between calculations and experiment for ^31^P-NMR is challenging,^56^ in part due to the inherent temperature dependence of ^31^P-NMR shifts.^57^ Moreover, errors arising from the use of the acid reference in DFT computations have been previously reported.^58^ Fortunately, an alternative approach where a secondary reference is used has been shown to improve agreement with the experiment.^59–61^ Several secondary references were tested; however, the **HNP** ^**pip**^ ligand and PH_3_ shifted the values in best agreement with experiment (see Supporting Information for details). Differences with experiment were reduced on average when using a secondary reference (Tables S15 and S16 and Figures 7 and S61). As expected, including the Hartree–Fock exchange (PBE0) improved agreement compared to the local PBE functional (Figure S61). Nevertheless, errors remain in the calculated results. Structural fluctuation and the ability of DFT to quantitatively recover the large spin–orbit shift are expected to contribute in this case.

**Figure 7:**
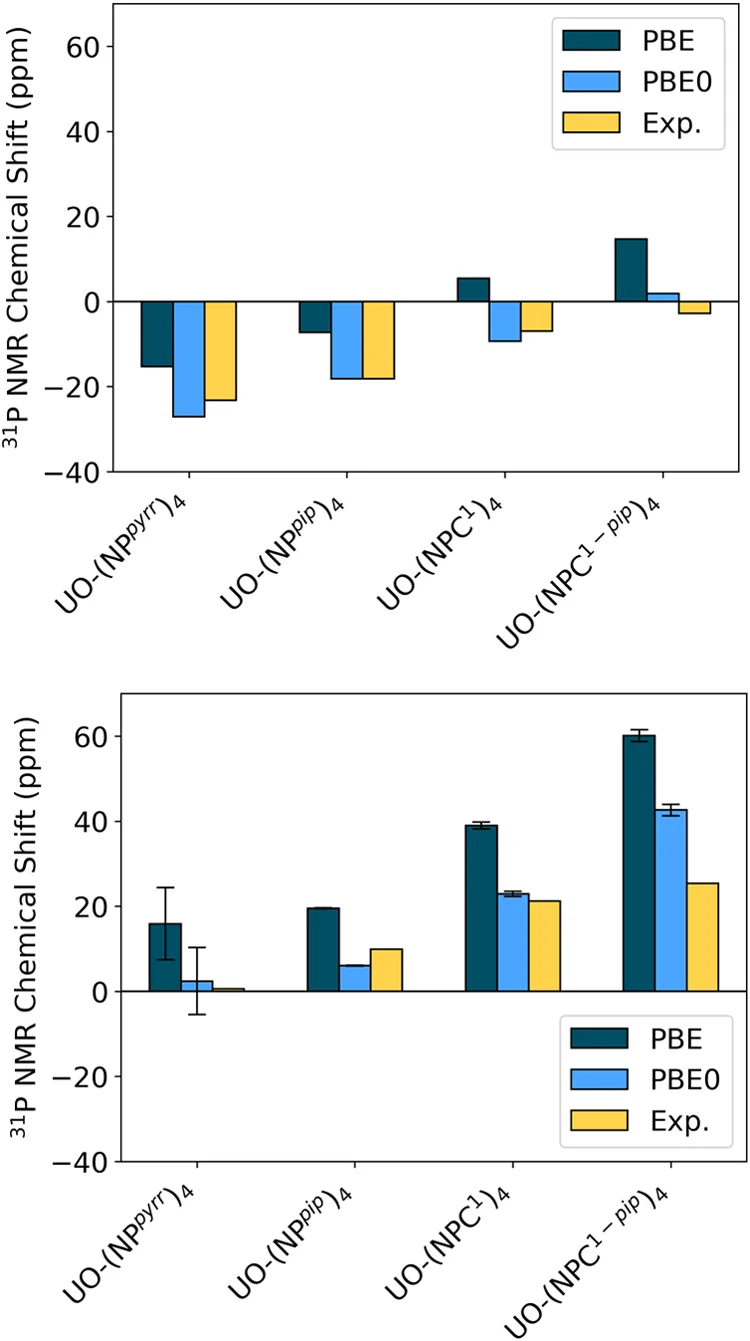
DFT and experimental ^31^P chemical shifts for the axial (top) and equatorial (bottom) ligands using the **HNP** ^**pip**^ ligand as the secondary reference. DFT values for the equatorial ligands are averages, and those for standard deviations are reported as error bars.

In addition to assigning the peaks as corresponding to the axial and equatorial ligands and reproducing the experimental trend, significant spin–orbit shifts of −41.2 ppm for the axial ligand and −25.9 ppm for the equatorial ligand are observed for **UO-(NP** ^**pyrr**^ **)**_**4**_ (Table S16). A larger spin–orbit shift has been shown to correlate with an increased orbital contribution to the bond.^62–65^ The large value obtained here is consistent with the presence of both σ and π interactions between the imidophosphorane ligand and the U^6+^ center. Likewise, the larger spin–orbit shift for the axial ligand supports an increased orbital interaction consistent with the assignment of the ITI. This extends beyond **UO-(NP** ^**pyrr**^ **)**_**4**_, since the average spin–orbit shift for the axial ligands in all four complexes is −40.2 ± 5.6 ppm, which is larger than the average for the equatorial ligands at −23.5 ± 4.2 ppm (Table S16), supporting the presence of the ITI in all four complexes.

## Discussion

Both crystallographic and dynamic NMR measurements demonstrate that the preferred low-temperature geometry of **UO-(NP** ^**pyrr**^ **)**_**4**_, **UO-(NP** ^**pip**^ **)**_**4**_, **UO-(NPC** ^**1**^ **)**_**4**_, and **UO-(NPC^1‑pip^)**_**4**_ is trigonal bipyramidal, featuring an imidophosphorane ligand positioned axial to the oxo ligand. Structural metrics based on SC-XRD data (of varying quality) indicate the presence of an ITI interaction for all complexes. This is supported by DFT calculations in which geometry-optimized structures have ca. 0.1 Å shorter U–N_axial_ than U–N_equat_ bond lengths. As expected, bond orders increase as bond distances decrease in values that are larger by 0.18–0.23 (Mayer) and 0.24–0.27 (NM-3) for the U–N_axial_ bonds. Additionally, stronger axial than equatorial bonds are observed by EDA (differences of −13.3 to −18.7 kcal/mol in total interaction energy) and ETS-NOCV (differences of −24.3 to −27.4 kcal/mol in the σ-type interactions) studies. NLMOs reveal an increased uranium orbital contribution (6.0–9.2%) for the U–N_axial_ σ bond in comparison to the U–N_equat_ σ bond, highlighting increased orbital mixing in the axial bond and a consequently stronger bond, further indicating the presence of an ITI interaction.

The four complexes show rapid ligand exchange at room temperature, supported by the observation of only a single resonance in the ^31^P­{^1^H}-NMR spectra that splits into two signals upon cooling below the coalescence temperature. Variable-temperature and EXSY-NMR studies enabled the determination of ligand-exchange barriers from 7.98(8) to 9.15(8) kcal/mol for the four complexes (Figure 8). Changing the ligand substituents does indeed result in different exchange barriers. These variations are consistent with an increase in the ITI for the four complexes in the following order: **UO-(NP** ^**pyrr**^ **)**_**4**_ < **UO-(NP** ^**pip**^ **)**_**4**_ < **UO-(NPC** ^**1**^ **)**_**4**_ < **UO-(NPC^1‑pip^)**_**4**_. As described previously,^24^ exchanging amide substituents for alkyl substituents increases the electron-donating effect. This is further modulated by the similar but different pyrrolidinyl and piperidinyl substituents. As electron donation of the imidophosphorane increases, the stabilization of the *trans*-oxo, and, in turn, the ligand-exchange barrier, is expected to increase. The exchange barrier reports to a transition-state energy that in solution is not just governed by the ITI but also other contributions, such as solution dielectric and intramolecular steric effects of the imidophosphorane ligand substituents. The computational studies provide a basis to parse the steric and electronic contributions to the experimentally observed exchange barriers. Recall that the ITI% is defined as the ratio of the axial to equatorial metal–ligand bond length. In other words, the trends across complexes associated with the ITI must involve the relative differences between the distance associated with the axial bond with respect to the equatorial bond. Plotting experimentally derived exchange barriers against the ITI% derived from the DFT optimized structures shows the expected correlation (Figure 9a). As Δ*G* increases, the ITI% decreases, indicating a strengthening ITI.

**Figure 8:**
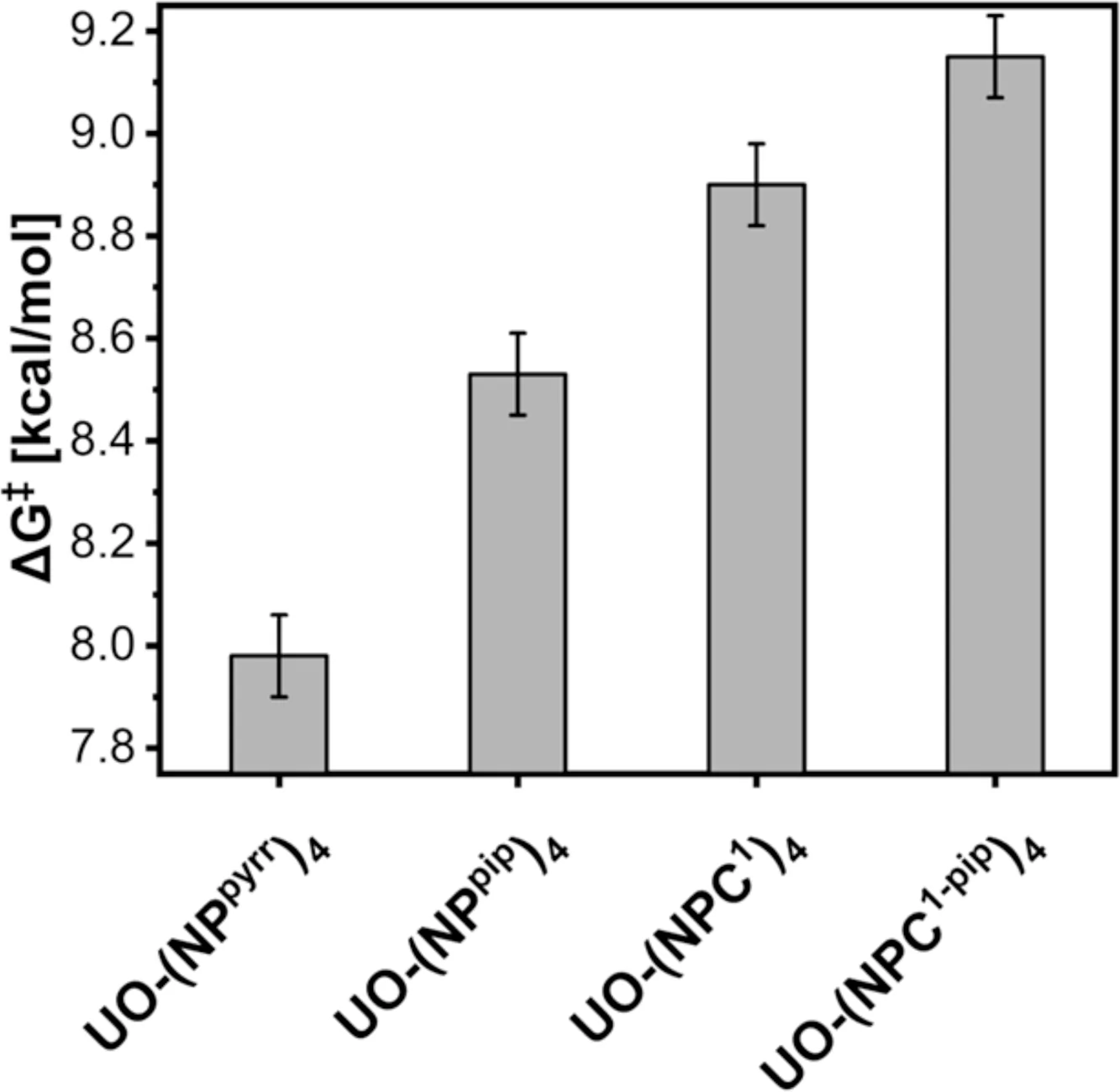
Experimentally derived ligand-exchange barriers (Δ*G* ^‡^) for the uranium mono-oxo complexes.

**Figure 9:**
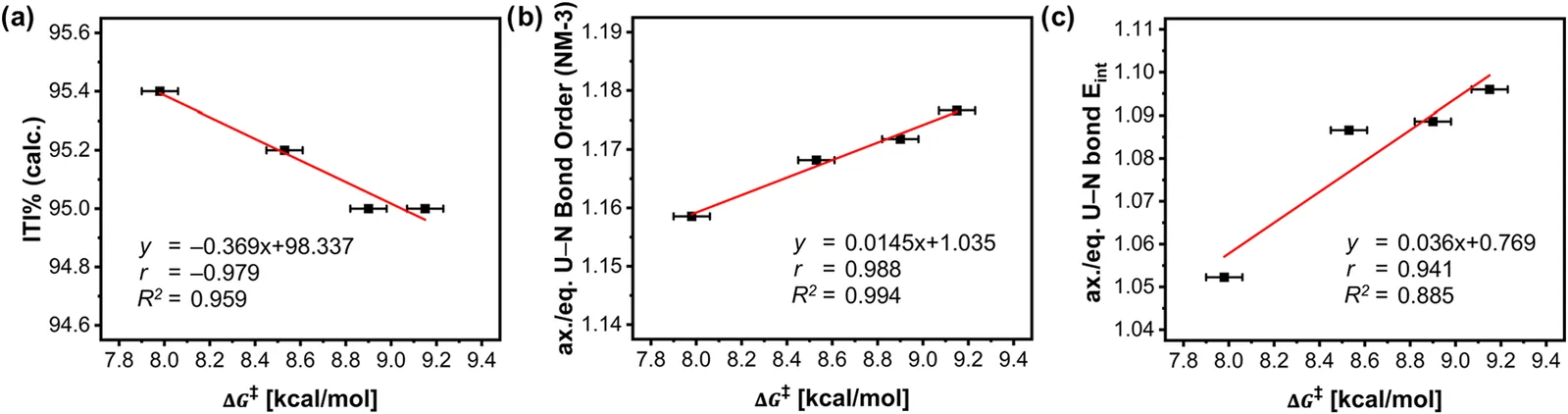
Correlation plots of experimental exchange barriers with (a) ITI% (from DFT structures), (b) ratio of ax./eq. U–N Nalewajski–Mrozek Type 3 bond orders, (c) ratio of ax./eq. U–N bond interaction energy (*E*_*i*nt_) derived from energy decomposition analysis (EDA).

Additionally, steric effects and structural flexibility can also contribute to the observed exchange barrier. With this in mind, the *G*-parameter was calculated to quantify the steric effects of the complex for a given ligand. However, no correlation between the *G*-parameter and Δ*G* ^‡^ is observed (Figure 10 and Table S27). It should be noted, however, that the piperidine derivatives always show a higher *G*-parameter and exchange barrier than their respective pyrrolidine analogues and that the *tert*-butyl group-containing derivatives in general show higher barriers. While steric effects can modulate barriers and present challenges for DFT modeling, the lack of correlation can be explained in two ways: (1) the *G*-parameter derived from minimum-energy structures based on solid-state measurements does not sufficiently represent the steric contribution to the exchange process, and (2) under experimental conditions, these ligands have sufficient structural flexibility to adopt lowest-energy conformers at both the minima and barrier that mask steric differences leading to the largest contribution to differences in Δ*G* ^‡^ arising from variations in the ITI bond strength.

**Figure 10:**
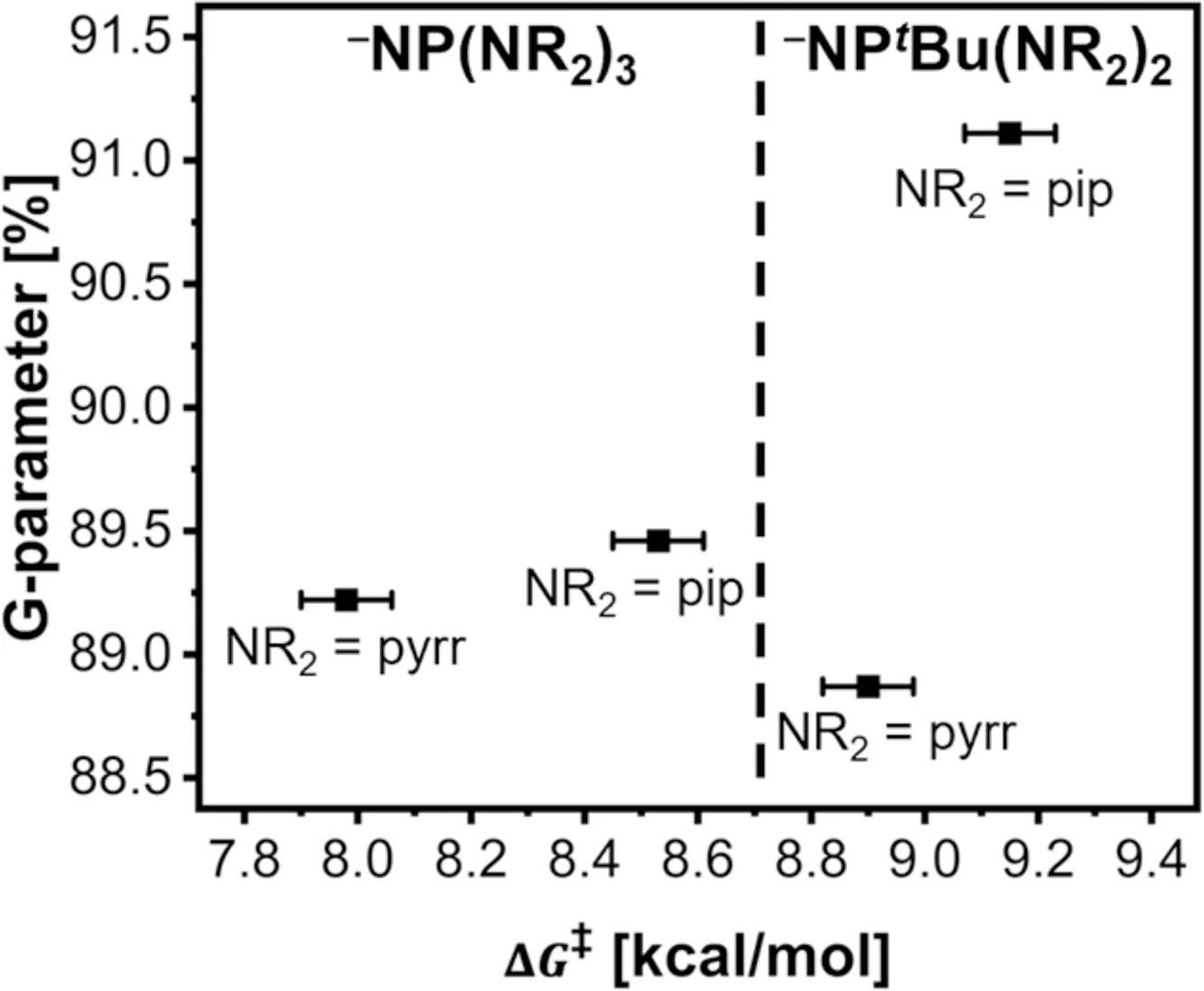
Correlation plot of experimental exchange barriers with the *G*-parameter derived from DFT geometries.

Moreover, correlations are observed when comparing the Δ*G* ^‡^ values with metrics that assess ITI strength and not structural features alone. In analogy to the ITI% parameter for crystallographic analysis, the changes in the axial and equatorial bond orders themselves cannot be interpreted in a meaningful way (Table 5); however, the trends in the ratio of axial to equatorial U–N bond order can delineate the small changes in these values (Figure 9b). A larger ratio denotes a higher bond order of the axial compared to equatorial bonds, indicating a stronger ITI. As the ratio increases, the exchange barrier increases across the series. Likewise, the trend in the difference between the axial and equatorial bond orders (Δ) matches that of the exchange barriers (Table 5). These trends indicate that the electronic stabilization afforded by the ITI is modulated by the ligand substituents and contributes to the exchange barrier.

**Table 5:** DFT-Derived Nalewajski–Mrozek Type 3 (NM-3) Bond Orders for the Axial and Equatorial U–N Bonds in Each Complex^a^

	**UO-(NP^pyrr^)_4_**	**UO-(NP^pip^)_4_**	**UO-(NPC^1^)_4_**	**UO-(NPC^1‑pip^)_4_**
axial	1.783	1.792	1.794	1.792
equat.	1.539	1.534	1.531	1.523
Δ	0.244	0.258	0.263	0.269
ratio	1.159	1.168	1.172	1.177

a The difference (Δ) and ratio (axial/equatorial) between the two ligand positions are also reported.

Another metric that captures the subtle bond strengthening of axial over equatorial U–N bonds is observed in interaction energies (*E*_int_) from EDA analysis. Plotting the ratio of axial vs equatorial U–N bond interaction energies vs the experimental ligand-exchange barriers (Figure 9c) again reveals a trend of an increasing *E*_int_ ratio with increasing Δ*G* ^‡^, further supporting the hypothesis that a larger exchange barrier indicates a stronger inverse *trans*-influence.

## Conclusion

This study is the first solution spectroscopic interrogation of the inverse *trans*-influence. Four uranium mono-oxo complexes with varying pyrrolidine-, piperidine-, and *tert*-butyl-substituted imidophosphorane ligands are investigated with dynamic NMR techniques and DFT. Identical ligands in the axial and equatorial positions allow for a reliable comparison of the two sites. The presence of phosphorus in the ligands provides an NMR handle sensitive to ligand exchange and metal–ligand bonding. These closed-shell singlet complexes enable the computation of the DFT-derived NMR chemical shifts and facilitate direct comparison with experiment. The complexes exhibit rapid ligand exchange at room temperature. Exchange barriers ranging from 7.98 to 9.15 kcal/mol were determined with variable-temperature and EXSY-NMR. The exchange barriers probe the energy difference to the transition state in the exchange process, which is not solely affected by the ITI but by an interplay of steric, electronic, solution dielectric, and solvent viscosity contributions. However, a modulation of the exchange barriers by different ligand substituents is observed, and the supporting DFT computations suggest that the difference in the ITI strength is the best metric to rationalize the trends observed. All four uranium mono-oxo complexes exhibit an ITI, as shown crystallographically and computationally. Correlations of Δ*G* ^‡^ with computationally derived metrics such as ITI%, bond orders, and EDA are observed.

The ITI potentially represents an opportunity to design ligands to match preferred structural features of high-oxidation-state actinides and effect separations from transition-metal and lanthanide ions. Therefore, detailing the solution dynamics of ligands that facilitate this preferred geometric distortion of the actinide coordination sphere can provide insights into ideal donor properties. While the ligands included in this study are electronically similar to each other, the sensitivity of the exchange barrier to comparably small changes in the ligand architecture suggests that the larger perturbation of substituents, architecture (chelating systems), or electronic properties could enable solution discrimination between metal ions.

## Experimental Section

General synthetic considerations and additional analytical details are reported in the Supporting Information. The syntheses of **UO-(NP** ^**pip**^ **)**_**4**_, **UO-(NPC^1‑pip^)**_**4**_, and **UO-(NPC** ^**1**^ **)**_**4**_ were carried out as previously described.^21,24^ In the NMR assignments below, 1 and 2 refer to the α and β positions of the pyrrolidine. Detailed spectra, including assignments, are provided in the SI.

*Caution!* Depleted uranium is primarily composed of ^238^U, a weak α-emitter (4.197 MeV, *t*_1/2_ = 4.47 × 10^9^ years). Manipulations should be carried out in a ventilated fume hood or glovebox in a radiation laboratory that is equipped with appropriate counting equipment.

### P^pyrr^

In a 500 mL Schlenk flask, 7.1 mL (11.18 g, 81.42 mmol, 1.00 equiv) of PCl_3_ in 250 mL of diethyl ether was cooled to 0 °C with an ice bath. Forty-eight milliliters (41.57 g, 584.51 mmol, 7.18 equiv) of pyrrolidine was added dropwise over 30 min via an addition funnel, resulting in the formation of a white precipitate. The resulting mixture was warmed to room temperature and stirred for 3 h. Inside a glovebox, the mixture was filtered, and the filtrate was reduced to a colorless oil *in vacuo*. Vacuum distillation (104 °C, 5 mTorr) afforded 18.44 g (93.9%) of the title compound as a colorless oil. ^1^H-NMR (400.13 MHz, C_6_D_6_): δ [ppm] = 3.21–3.04 (m, 12H, H-1), 1.68–1.51 (m, 12H, H-2). ^13^C­{^1^H}-NMR (100.62 MHz, C_6_D_6_): δ [ppm] = 47.4 (d, ^2^ *J*_CP_ = 16.5 Hz, C-1), 26.0 (d, ^3^ *J*_CP_ = 4.8 Hz, C-2). ^31^P­{^1^H}-NMR (161.97 MHz, C_6_D_6_): δ [ppm] = 104.2 (s). Elemental analysis was not collected on this air-sensitive liquid.

### HNP^pyrr^

HNP^pyrr^ is synthesized in a two-step procedure. *Step 1*: In a 200 mL Schlenk flask, 6.8 mL (5.96 g, 51.73 mmol, 1.50 equiv) of trimethylsilyl azide was added dropwise over 10 min by syringe to a solution of 8.31 g (34.44 mmol, 1.00 equiv) of **P** ^**pyrr**^ in 40 mL of toluene. The mixture was brought to reflux for 1 h. Volatiles were removed *in vacuo* to afford a colorless oil of Me_3_SiNP­(pyrr)_3_ and used directly in *Step 2* without further purification. ^1^H-NMR (400.13 MHz, C_6_D_6_): δ [ppm] = 3.12–2.99 (m, 12H, H-1), 1.58–1.49 (m, 12H, H-2), 0.46–0.41 (m, 9H, H-3). ^13^C­{^1^H}-NMR (100.62 MHz, C_6_D_6_): δ [ppm] = 46.9 (d, ^2^ *J*_CP_ = 4.3 Hz, C-1), 26.7 (d, ^3^ *J*_CP_ = 8.0 Hz, C-2), 5.1 (d, ^3^ *J*_CP_ = 2.8 Hz, C-3). ^31^P­{^1^H}-NMR (161.97 MHz, C_6_D_6_): δ [ppm] = 1.5 (s).

*Step 2*: In a 200 mL Schlenk flask, Me_3_SiNP­(pyrr)_3_ (used directly from *Step 1*) was dissolved in 30 mL of methanol. Two drops of concentrated H_2_SO_4_ (96%) were added, and the mixture was stirred for 36 h at room temperature. The volatiles were removed *in vacuo*, and the residue was purified by vacuum distillation (75 °C, 5 mTorr) to afford the title compound (7.04 g, 79.8%) as a clear colorless liquid. ^1^H-NMR (400.13 MHz, C_6_D_6_): δ [ppm] = 3.20–3.07 (m, 12H, H-1), 1.60–1.48 (m, 12H, H-2), 0.58 (s, 1H, H-3). ^13^C­{^1^H}-NMR (100.62 MHz, C_6_D_6_): δ [ppm] = 47.0 (d, ^2^ *J*_CP_ = 3.3 Hz, C-1), 26.7 (d, ^3^ *J*_CP_ = 7.1 Hz, C-2). ^31^P­{^1^H}-NMR (161.97 MHz, C_6_D_6_): δ [ppm] = 28.5 (s). Elemental analysis was not collected on this air-sensitive liquid.

### KNP^pyrr^

In a 50 mL Schlenk flask, 6.66 g (25.98 mmol, 1.00 equiv) of **HNP** ^**pyrr**^ was dissolved in 20 mL of hexane. 3.38 g (25.95 mmol, 1.00 equiv) of potassium benzyl was added as a solid, and any residuals were rinsed into the mixture with 15 mL of hexane. After stirring for 12 h at room temperature, the mixture was filtered through a fine-porosity glass-fritted funnel. The solid was dried and then redissolved in 15 mL of THF and filtered through a fine-porosity glass-fritted funnel packed with Celite. Volatiles were removed *in vacuo*, and the residue was redissolved in 10 mL of diethyl ether and filtered through a fine-porosity glass-fritted funnel, and the filter cake was washed with 10 mL of diethyl ether and then 30 mL of pentane. The filtrate was reduced *in vacuo* and afforded the title compound (5.82 g, 76.1%) as a white solid. ^1^H-NMR (400.13 MHz, C_6_D_6_): δ [ppm] = 3.24–3.09 (m, 12H, H-1), 1.88–1.73 (m, 12H, H-2). ^13^C­{^1^H}-NMR (100.62 MHz, C_6_D_6_): δ [ppm] = 47.6 (s, C-1), 27.0 (d, ^3^ *J*_CP_ = 7.1 Hz, C-2). ^31^P­{^1^H}-NMR (161.97 MHz, C_6_D_6_): δ [ppm] = −9.5 (s). FT-IR (ATR): ν [cm^–1^] = 2957 (w), 2806 (w), 1443 (vw), 1220 (s), 1122 (w), 1045 (m), 972 (s), 911 (w), 872 (vw), 749 (w), 561 (m), 548 (s), 515 (w). Elemental analysis found (calcd.) for C_12_H_24_KN_4_P: C, 48.95 (49.02); H, 8.22 (8.38); N, 19.03 (19.21).

### U­(NP^pyrr^)_4_

In a 20 mL scintillation vial with a glass stir bar, UI_4_(dioxane)_2_ (200.2 mg, 0.217 mmol, 1.00 equiv) was dissolved in 4 mL of Et_2_O. **KNP** ^**pyrr**^ (257.1 mg, 0.873 mmol, 4.02 equiv) was added as a solid, followed by 6 mL of Et_2_O. The brown suspension was stirred at 35 °C for 16 h. The reaction mixture was filtered through a pipette packed with glass fiber filter paper and Celite, and the solvent was removed *in vacuo*. The orange residue was triturated two times with 7 mL of pentane prior to being taken up in 9 mL of pentane and filtered through a pipette packed with glass fiber filter paper and Celite. The filtrate was concentrated *in vacuo* and placed in a −35 °C freezer. Overnight, pale pink XRD-quality crystals were formed at the bottom of the vial. These were isolated by decantation, dissolved in 3 mL of pentane (to separate from orange crystals higher up in the vial), followed by removing all volatiles *in vacuo* to afford the title compound (136.6 mg, 50.0%) as pale pink crystalline powder. A second crop of crystals was obtained from the supernatant (28.8 mg, 10.5%). Combined yield: 165.4 mg (60.5%). ^1^H-NMR (500.13 MHz, C_6_D_6_): δ [ppm] = 3.72 (s, 48H, H-1), 1.15 (s, 48H, H-2). ^13^C­{^1^H}-NMR (125.77 MHz, C_6_D_6_): δ [ppm] = 27.2 (s, C-1), 22.7 (s, C-2). ^31^P­{^1^H}-NMR (202.48 MHz, C_6_D_6_): δ [ppm] = 309.6 (s). FT-IR (ATR): ν [cm^–1^] = 2954 (w), 2836 (w), 1455 (vw), 1192 (w), 1149 (s), 1067 (m), 986 (m), 909 (m), 871 (w), 757 (w), 552 (m). Elemental analysis found (calcd.) for C_48_H_96_N_16_P_4_U: C, 45.78 (46.04); H, 7.68 (7.67); N, 17.80 (18.05).

### UO-(NP^pyrr^)_4_

In a 20 mL scintillation vial with a glass stir bar, **U­(NP** ^**pyrr**^ **)**_**4**_ (165 mg, 0.13 mmol) was dissolved in 3 mL of THF. A balloon was filled with N_2_O from a lecture bottle and then affixed directly to the neck of the vial. The solution was rigorously stirred for 3 h and turned dark red-brown. Volatiles were removed *in vacuo*. The residue was triturated twice with 5 mL of pentane prior to being taken up in 8 mL of pentane and filtered through a pipette packed with glass fiber filter paper and Celite. The filtrate was concentrated *in vacuo* and stored at −35 °C overnight to yield dark red-brown crystals. These were isolated by decantation, and remaining volatiles were removed *in vacuo* to afford the title compound (83.9 mg, 50.2%) as a dark red-brown crystalline powder. Crystals suitable for SC-XRD were grown from pentane at −35 °C. ^1^H-NMR (400.13 MHz, THF-*d*_8_): δ [ppm] = 3.44–3.33 (m, 48H, H-1), 1.77–1.66 (m, 48H, H-2). ^1^H-NMR (400.13 MHz, C_6_D_6_): δ [ppm] = 3.67–3.55 (m, 48H, H-1), 1.87–1.74 (m, 48H, H-2). ^13^C­{^1^H}-NMR (100.62 MHz, C_6_D_6_): δ [ppm] = 47.2 (d, ^2^ *J*_CP_ = 3.1 Hz, C-1), 27.2 (d, ^3^ *J*_CP_ =7.4 Hz, C-2). ^31^P­{^1^H}-NMR (161.99 MHz, C_6_D_6_): δ [ppm] = −1.8 (s). ^31^P­{^1^H}-NMR (161.99 MHz, THF-*d*_8_): δ [ppm] = −2.7 (s). ^31^P­{^1^H}-NMR (202.48 MHz, THF-*d*_8_, −95 °C): δ [ppm] = 0.6 (s, 3P, P_eq_), −23.2 (s, 1P, P_ax_). FT-IR (ATR): ν [cm^–1^] = 2953 (w), 2841 (w), 1455 (vw), 1194 (w), 1108 (s), 1057 (s), 990 (s), 909 (m), 869 (w), 751 (m), 550 (m), 417 (vw). Elemental analysis found (calcd.) for C_48_H_96_N_16_OP_4_U: C, 45.21 (45.46); H, 7.59 (7.55); N, 17.57 (17.82).

## Computational Section

DFT gas phase geometry optimizations were performed as implemented in the Turbomole V7.8.1 program package.^66^ A basis set of triple-ζ quality is used. Specifically, def-TZVP and its corresponding effective core potential were used for uranium, while the def2-TZVP basis set was employed for the remaining atoms.^67–70^ Since all complexes contain U^6+^ centers, the structures are optimized for the closed-shell singlet state. The impact of functional choice was explored by employing the PBE, TPSS, and MN15-L functionals.^71–73^ The resolution of identity (RI) approximation was used for integral evaluation in all calculations.^70^ The SCF energy was converged to 10^–7^ a.u., and the Cartesian gradient was converged to 10^–4^ a.u. The grid size was selected using the m4 keyword. All structures were confirmed as minima using the standard harmonic approach, although in some cases, small imaginary frequencies were observed associated with peripheral groups on the sterically bulky ligands.

Using the Amsterdam Modeling Suite (AMS) software package (version 2024.105),^74^ single-point calculations were performed on TPSS-optimized geometries. The TPSS functional was used in conjunction with a Slater-type orbital (STO) basis set of TZP quality for all atoms.^75^ The only exception is that for the NMR computations, the PBE or PBE0 functionals were employed, since the use of metaGGA functionals has not been implemented to compute the chemical shifts.^71,76^ Analogous calculations were also performed on molecules used as NMR references; specifically, H_3_PO_4_, PPh_3_, and the protonated form of the four ligands of interest (**HNP** ^**pyrr**^, **HNP** ^**pip**^, **HNPC^1^**, **HNPC^1‑pip^**). The scalar-relativistic (SR) all-electron zero-order regular approximation (ZORA) Hamiltonian was used;^77^ specifically, Mayer and Nalewajski–Mrozek Type 3 (NM-3) bond orders and parameters derived from the quantum theory of atoms in molecules (QTAIM) were computed.^78–80^ To quantify the compositions of the chemical bonds of interest, natural localized molecular orbital (NLMO) analyses were carried out with the NBO 7.0 program as interfaced with AMS.^81^ Energy decomposition analysis (EDA) and extended transition state, natural orbitals for chemical valence (ETS-NOCV) analysis, were also performed at the same level of theory.^82^ Two sets of fragments were analyzed. First, the axial ligand was selected as one anionic fragment, with the rest of the molecule making up the other cationic fragment. Then, an analogous computation was performed in which one of the equatorial ligands was selected as the fragment. The computations were repeated using Grimme’s D3 correction with Becke–Johnson damping to assess the impact of dispersion on the interaction energies.^83,84^ Finally, ^31^P nuclear magnetic shielding calculations were also performed using both the scalar-relativistic and the spin–orbit ZORA Hamiltonians (SR-ZORA, SO-ZORA). The SO-ZORA shielding calculations included the DFT exchange–correlation response.^85,86^ The calculated nuclear shielding constants (σ) were converted to chemical shifts (δ) relative to shielding of both phosphoric acid (H_3_PO_4_) or the free ligands calculated at the same level of theory (i.e., PBE/TZP or PBE0/TZP).

In addition to understanding the structures as characterized in the solid state, we hypothesized that the variable-temperature NMR results could be rationalized via interconversion through a square-pyramidal structure. Geometry optimizations using the TPSS-D3 functional and the COSMO solvation model were performed for **UO-(NP** ^**pip**^ **)**_**4**_ as implemented in the Turbomole program.^87^ Note that while minimal structural changes were obtained in the synthesized structures when solvation and dispersion corrections were applied, these corrections were essential in describing reaction energies for systems with bulky ligand groups. EDA and ETS-NOCV analyses were also performed on this potential barrier structure, as described previously.

## Supplementary Material



## Data Availability

Computational data including input and output files are available in a FigShare repository. https://doi.org/10.6084/m9.figshare.32764770.

## Notes

Additional experimental considerations, NMR, UV–vis–NIR, and IR analyses, and computational and crystallographic details (PDF)
