# Supplementary material for: Solution Spectroscopic Analysis of Ligand Exchange in Uranium Mono-Oxo Complexes
Source: Inorg Chem. 2026 Oct 6;65(41):24331–42. doi: 10.1021/acs.inorgchem.6c03607 (PMC13685417; doi:10.1021/acs.inorgchem.6c03607)
Supplement: Supplementary file 1 [file ic6c03607_si_001.pdf]

Supporting Information

Solution Spectroscopic Analysis of Ligand Exchange in Uranium Mono-Oxo Complexes

Florian Ressnik,† Tyler-Rayne Nero,† Maximilian G. Bernbeck,† Johannes Leisen,† Bess Vlaisavljevich,*‖ Julie E. Niklas,*† and Henry S. La Pierre*†‡#

†School of Chemistry & Biochemistry, Georgia Institute of Technology, Atlanta, GA 30332, United States

‡Nuclear and Radiological Engineering and Medical Physics Program in the School of Mechanical Engineering, Georgia Institute of Technology, Atlanta, Georgia 30332, United States

#Physical Sciences Division, Pacific Northwest National Laboratory, Richland, Washington 99352, United States

‖Department of Chemistry, The University of Iowa, Iowa City, IA 52242, United States

Corresponding author email addresses:

Henry S. La Pierre: hsl@gatech.edu
Bess Vlaisavljevich: bess-vlaisavljevich@uiowa.edu
Julie E. Niklas: j.niklas@gatech.edu

Table of Contents

1 General synthetic and analytical details 2
  1.1 Procedures, solvents and chemicals 2
  1.2 Analytical techniques 2
2 Synthesis 3
3 NMR Spectroscopy 4
  3.1 1D and 2D NMR spectra 4
  3.2 VT and EXSY NMR Analysis 14
  3.3 Variable Temperature NMR spectra 18
  3.4 Exchange Spectroscopy (EXSY) NMR spectra 22
4 UV-vis-NIR Spectroscopy 28
5 IR Spectroscopy 31
6 SC-XRD 32
7 Computational Supporting Data 35
  7.1 DFT Geometry Optimizations 35
  7.2 Bond Order Results 38
  7.3 Computed ^{31}P-NMR Parameters 39
  7.4 Natural Localized Molecular Orbitals 45
  7.5 EDA and ETS-NOCV Results 49
  7.6 Selected Structure Proposed as Barrier 53
References 55

1 General synthetic and analytical details

1.1 Procedures, solvents and chemicals

Unless stated otherwise, all reactions and procedures were carried out under air and water free conditions by using Schlenk and glovebox (Vigor, N_2 atmosphere, <0.1 ppm O_2/H_2O) techniques. Glassware used in the glovebox was stored overnight in the oven at 160 °C and cycled in hot. All glassware used on the Schlenk line was stored overnight in the oven at 160 °C or flame dried under vacuum prior use. Molecular sieves and Celite were heated (>250 °C) under vacuum for a minimum of 24 h.

Tetrahydrofuran (THF), *n*-pentane, *n*-hexane, diethyl ether (Et_2O) and toluene were collected dry (passed through columns containing Q-5/alumina and molecular sieves) and degassed (purged with UHP-grade Argon (Airgas)) from a commercial solvent purification system (JC Meyer Solvent Systems, Pure Process Technology) and then stored over 10 % (v/v) 3 Å molecular sieves in media bottles inside the glovebox. A saturated solution of purple Na^0/benzophenone ketyl in THF was used to periodically verify solvent dryness. Deuterated solvents (THF-d_8 and C_6D_6 from Cambridge Isotope Laboratories) were degassed by 3-4 freeze-pump-thaw cycles, vacuum transferred from purple Na^0/benzophenone and then stored over molecular sieves (3 Å). Methanol (MeOH) was dried by refluxing over magnesium turnings (activated with iodine) overnight and then distilled and degassed (sparged with N_2 or Ar inert gas) and stored over molecular sieves (3 Å).

The syntheses of potassium benzyl (KBn), UI_4(dioxane)$_2$, dbabhNO, **UO-(NP^{pip})$_4$**, **UO-(NPC^{1-pip})$_4$** and **UO-(NPC^1)$_4$** were carried out as previously described in the literature.[1-5]

Pyrrolidine (>99 %, Thermo Scientific Chemicals) was refluxed over CaH_2 and then distilled prior use.

N_2O (99.99 %, Matheson), trimethylsilyl azide (94 %, Thermo Scientific Chemicals), phosphorus trichloride (99 %, Sigma-Aldrich) and sulfuric acid (96 %, Thermo Scientific Chemicals) were used as received.

1.2 Analytical techniques

NMR Spectroscopy: NMR data were collected on Bruker Avance III 400 and Bruker Avance III HD 500 spectrometer. Spectra were recorded (unless otherwise stated) at 25 °C using the deuterated solvents THF-d_8 and C_6D_6. ^{1}H- and ^{13}C-NMR spectra were referenced regarding the deuterated solvent. ^{31}P-NMR spectra were referenced indirectly regarding H_3PO_4 (not physically present) with Ξ-values based on a referenced ^{1}H-NMR spectrum. Chemical shifts δ are expressed in ppm and coupling constants *J* in Hz. The data was processed with the program TopSpin 4.4.0 and MestReNova.[6]

Single Crystal X-ray Crystallography: Crystals suitable for X-ray diffraction were coated in NVH oil in a glove box and transferred to the diffractometer in a 20 mL capped vial and then mounted on a nylon or MiTeGen loop. X-ray crystallographic data were collected using a Bruker D8 VENTURE dual wavelength Mo/Cu four-circle diffractometer with a microfocus sealed X-ray tube using a mirror optics as monochromator and a Bruker PHOTON III detector. Crystals were cooled and kept at T = 100(2) K during data collections using an Oxford Cryostream 800 low temperature device. Data integration and reduction were undertaken with SAINT V8.40B[7] in the APEX5 software suite (Bruker). Multi-scan corrections were applied using SADABS[8] as implemented in APEX5. Structures were solved using SHELXT[9] and refined using SHELXL[10] and by using the graphical interface Olex2 1.5-alpha.[11] All hydrogen atoms were refined

isotropic on calculated positions using a riding model with their U_{iso} values constrained to 1.5 times the U_{eq} of their pivot atoms for terminal sp^3 carbon atoms and 1.2 times for all other carbon atoms. The final cif files have been created with the help of FinalCif.[12] Molecular structure pictures were created using Mercury 2024.1.0.[13]

Infrared (IR) Spectroscopy: Infrared (IR) spectra of powder samples were recorded on a Bruker ALPHA FTIR spectrometer from 400 cm^{-1} to 4,000 cm^{-1}, using an attenuated total reflection (ATR) attachment inside a N_2 glovebox. The peaks are listed in wavenumbers [cm^{-1}] and the intensities of the peaks are reported using the following abbreviations: vw (very weak), w (weak), medium (medium), s (strong).

UV-vis-NIR Spectroscopy: UV-vis-NIR spectra were recorded on a Hitachi UH4150 UV-vis-NIR scanning spectrophotometer from 1600 to 200 nm in small-volume screw-cap quartz cuvettes (Starna Scientific) with a 1 cm path length.

Elemental Analysis: Elemental Analysis was performed on C, H, N at the University of Iowa MATFab Facility.

2 Synthesis

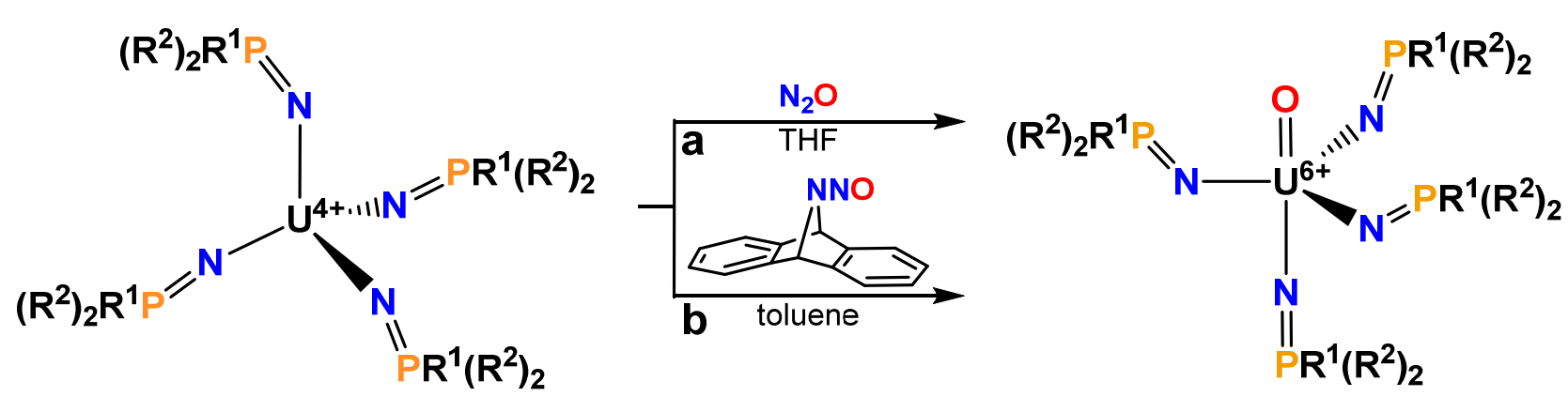

**U(NP^{pyrr})$_4$:**	R^1=R^2=pyrr	**UO-(NP^{pyrr})$_4$**	(50 %, method **a**)
**U(NP^{pip})$_4$:**	R^1=R^2=pip	**UO-(NP^{pip})$_4$**	(84 %, method **a**)
**U(NPC^1)$_4$:**	R^1=^{t}Bu, R^2=pyrr	**UO-(NPC^1)$_4$**	(92 %, method **a**)
**U(NPC$^{1\text{-}pip}$)$_4$:**	R^1=^{t}Bu, R^2=pip	**UO-(NPC$^{1\text{-}pip}$)$_4$**	(44 %, method **b**)

Figure S1: General synthesis of **UO-(NP)$_4$** complexes.[4, 5]

All described uranium mono-oxo complexes were synthesized as summarized in **Figure S1**.

3 NMR Spectroscopy

3.1 1D and 2D NMR spectra

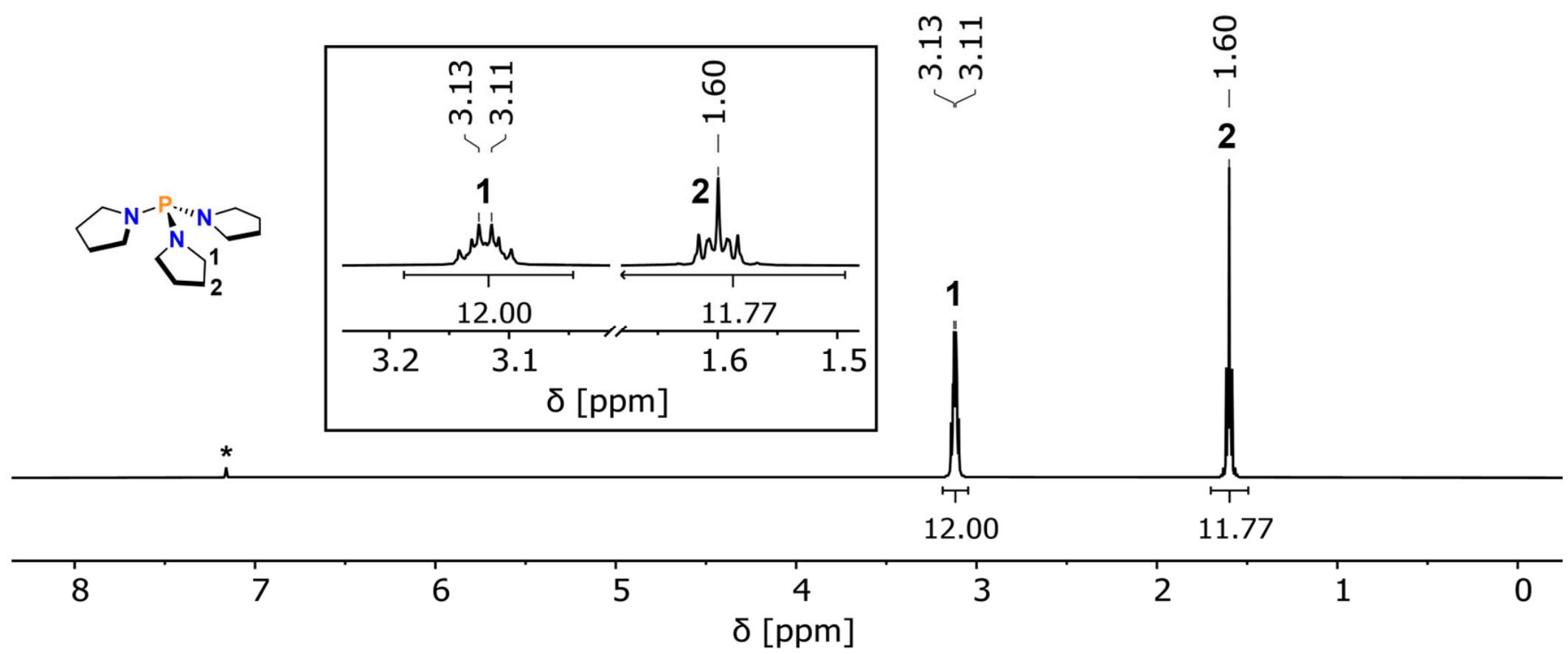

Figure S2: ^{1}H-NMR spectrum of **P^{pyrr}** in C_6D_6. Residual solvent signal is marked with *.

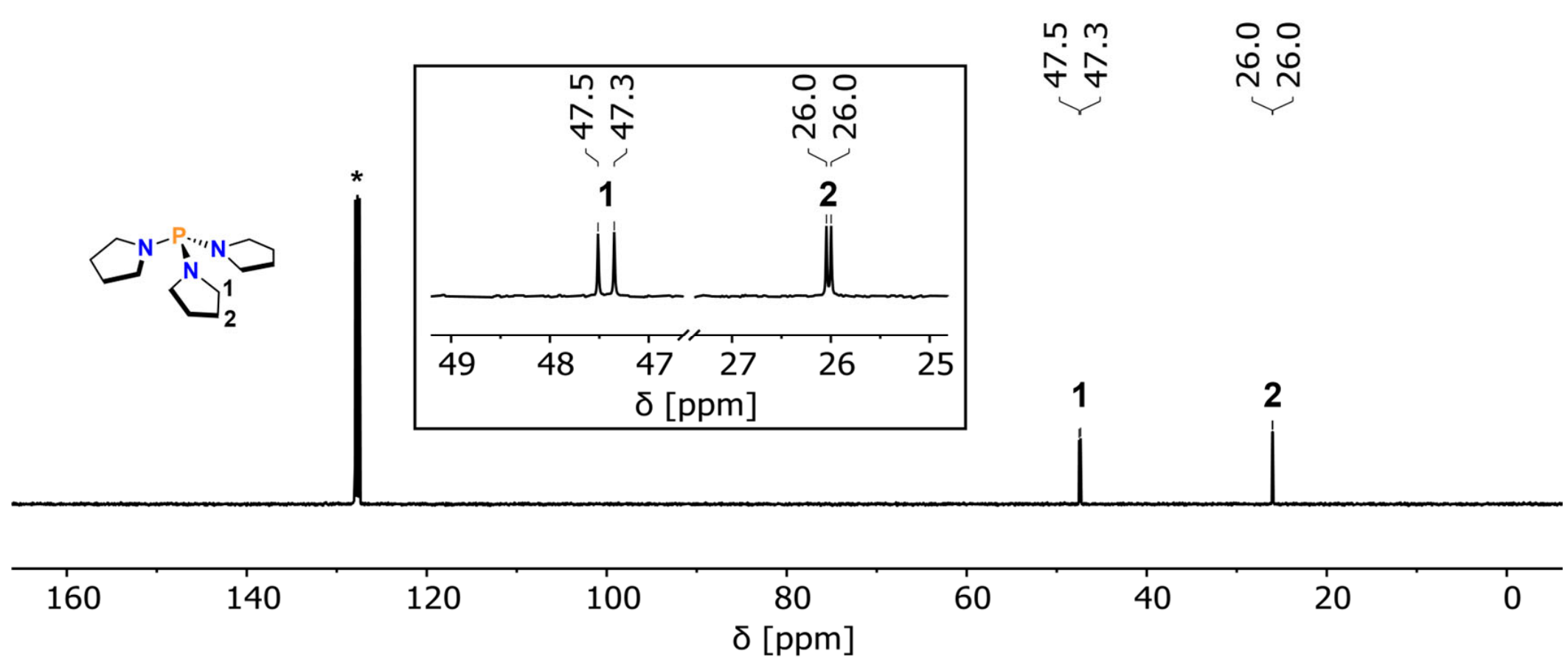

Figure S3: $^{13}C\{^{1}H\}$-NMR spectrum of **P^{pyrr}** in C_6D_6. Residual solvent signal is marked with *.

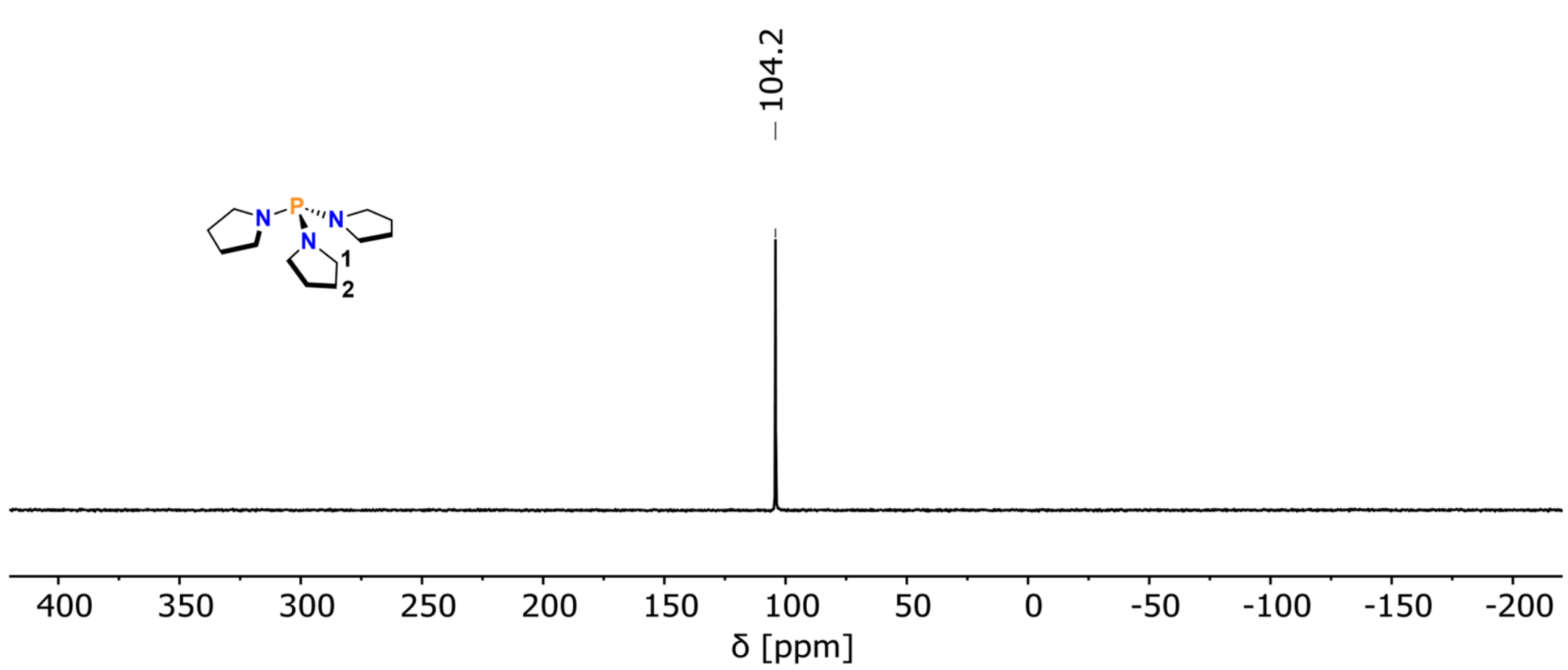

Figure S4: $^{31}P\{^{1}H\}$-NMR spectrum of **P^{pyrr}** in C_6D_6.

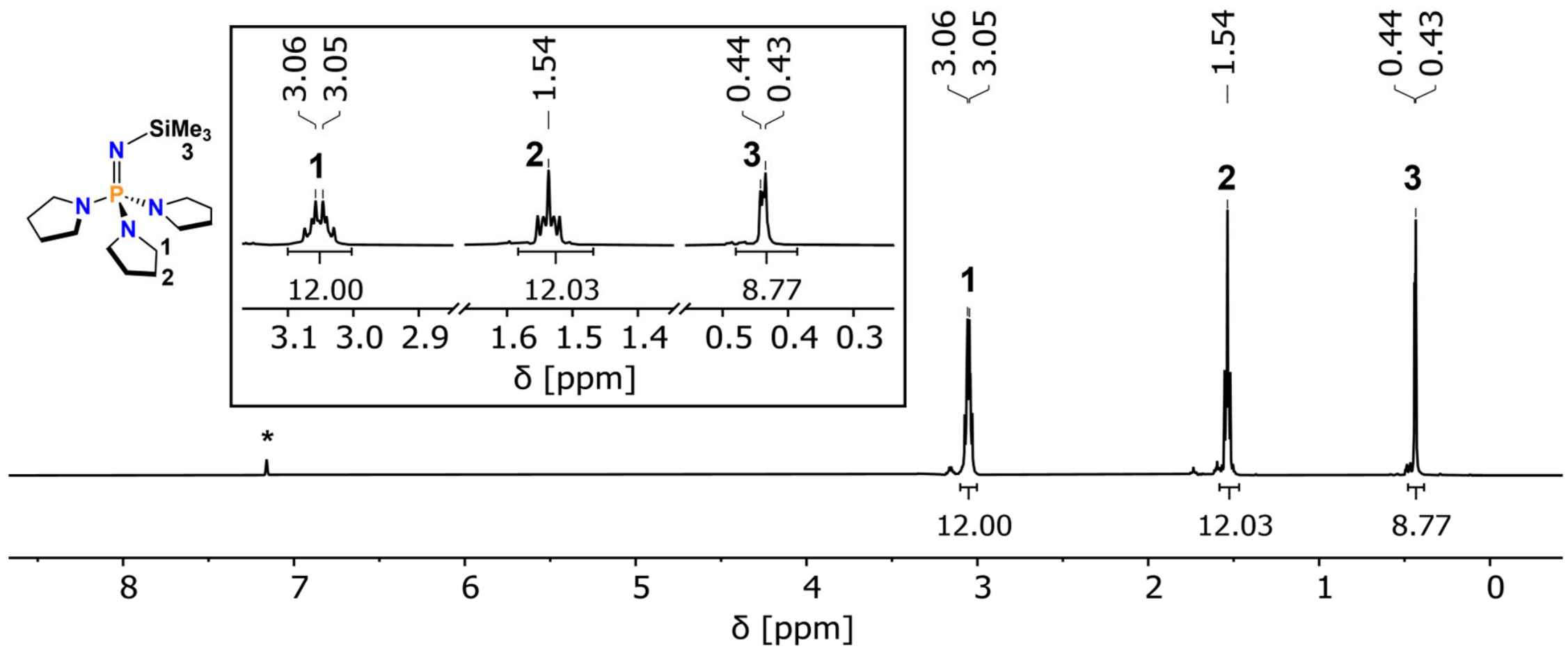

Figure S5: ^{1}H-NMR spectrum of crude **$Me_3SiNP(pyrr)_3$** in C_6D_6. Residual solvent signal is marked with *.

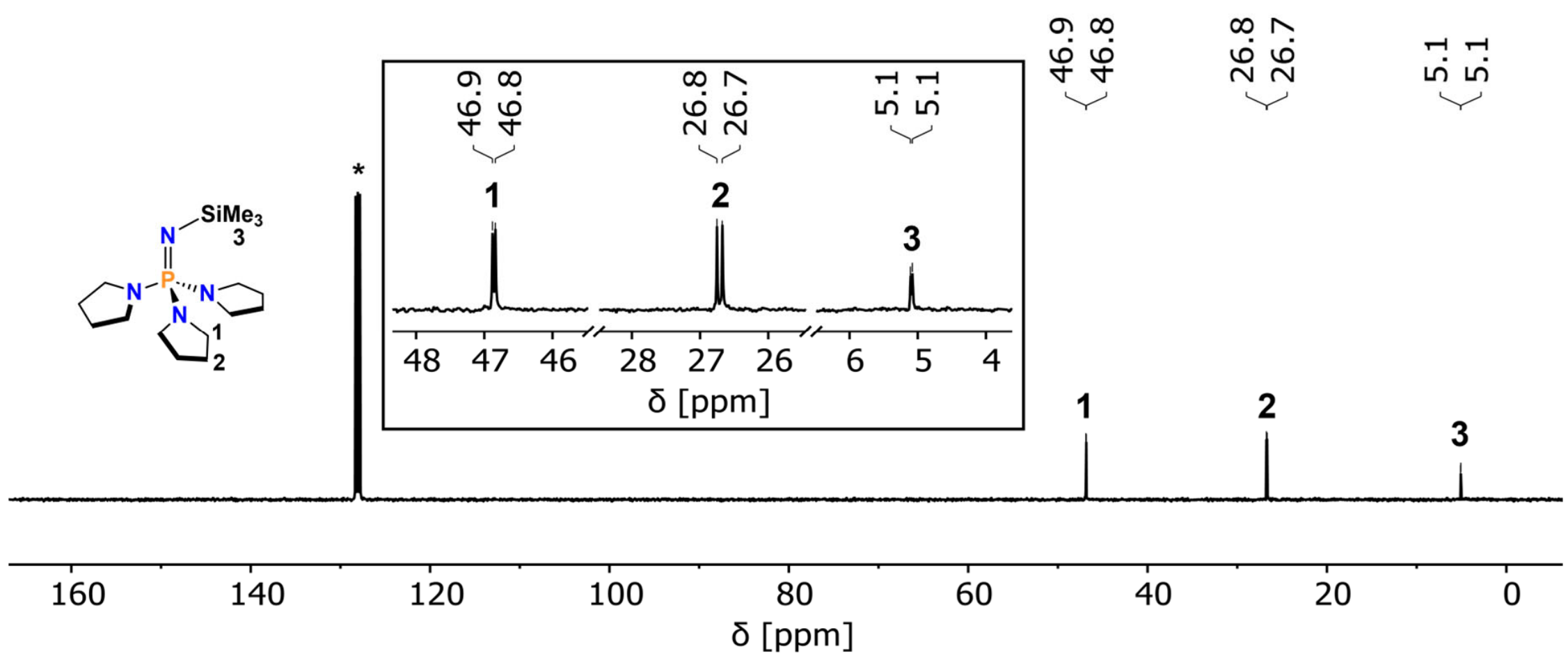

Figure S6: $^{13}C\{^{1}H\}$-NMR spectrum of crude **$Me_3SiNP(pyrr)_3$** in C_6D_6. Residual solvent signal is marked with *.

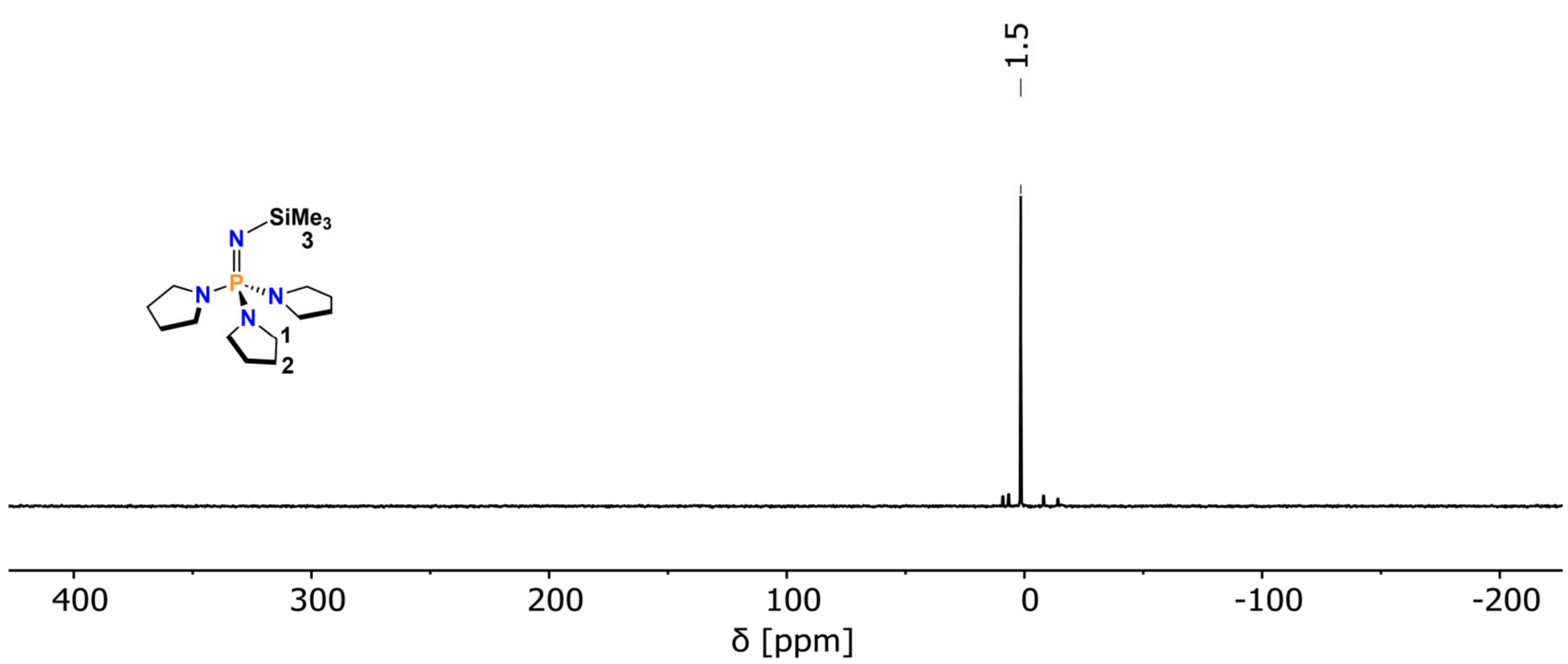

Figure S7: $^{31}P\{^{1}H\}$-NMR spectrum of crude **$Me_3SiNP(pyrr)_3$** in C_6D_6.

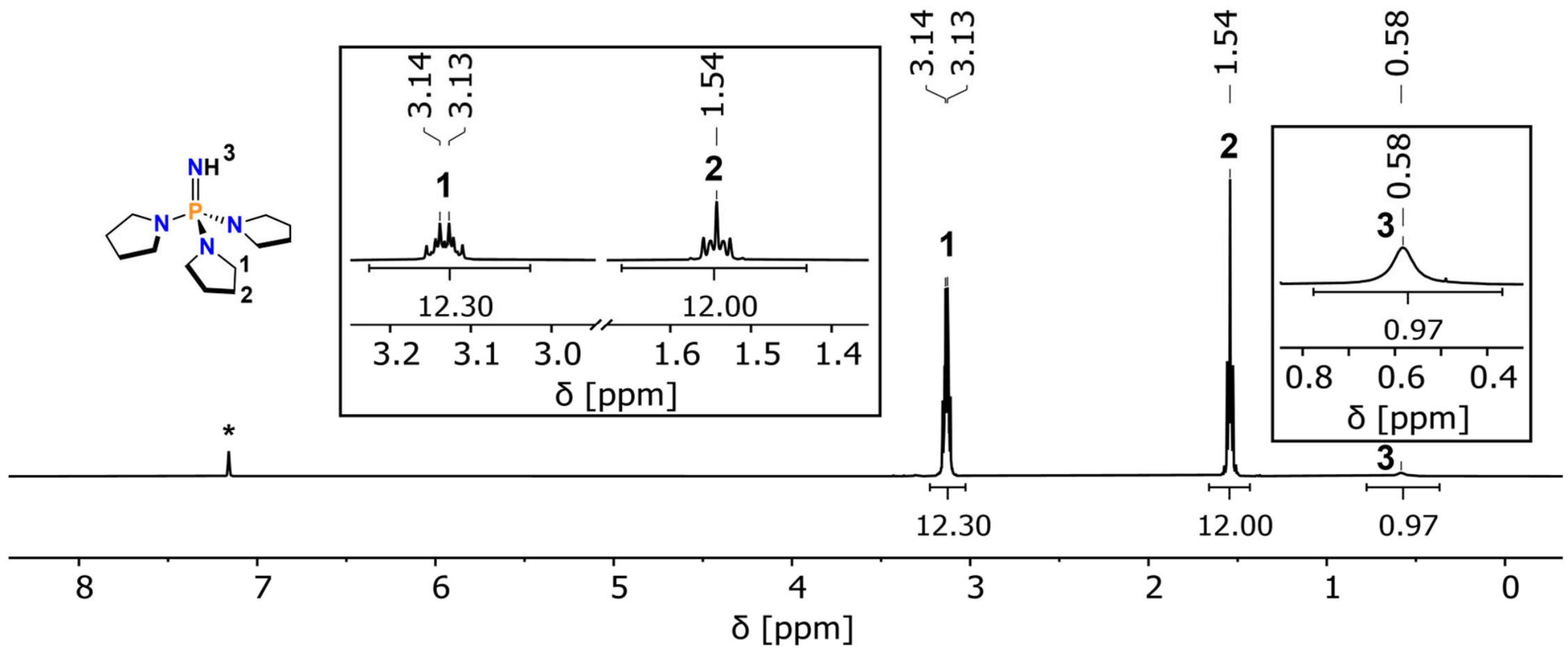

Figure S8: ^{1}H-NMR spectrum of **HNP^{pyrr}** in C_6D_6. Residual solvent signal is marked with *.

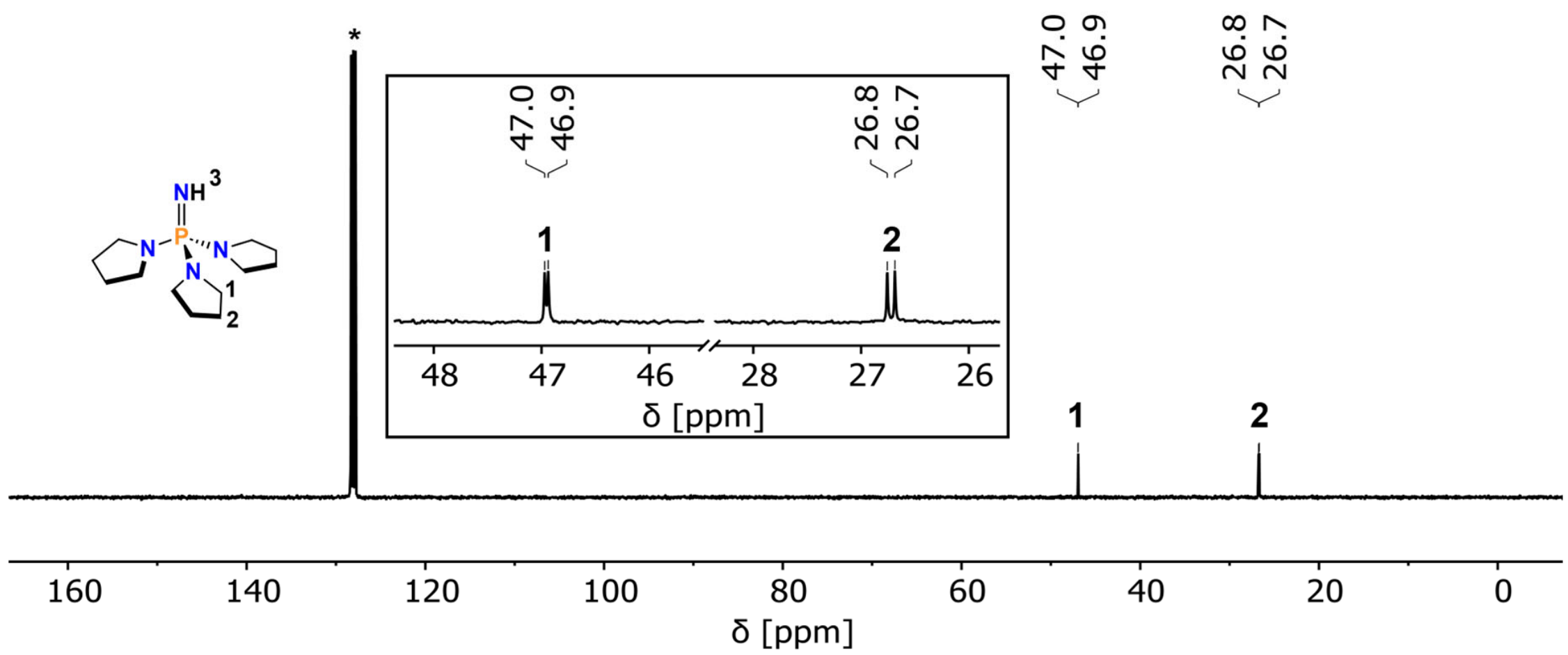

Figure S9: ^{13}C{^{1}H}-NMR spectrum of **HNP^{pyrr}** in C_6D_6. Residual solvent signal is marked with *.

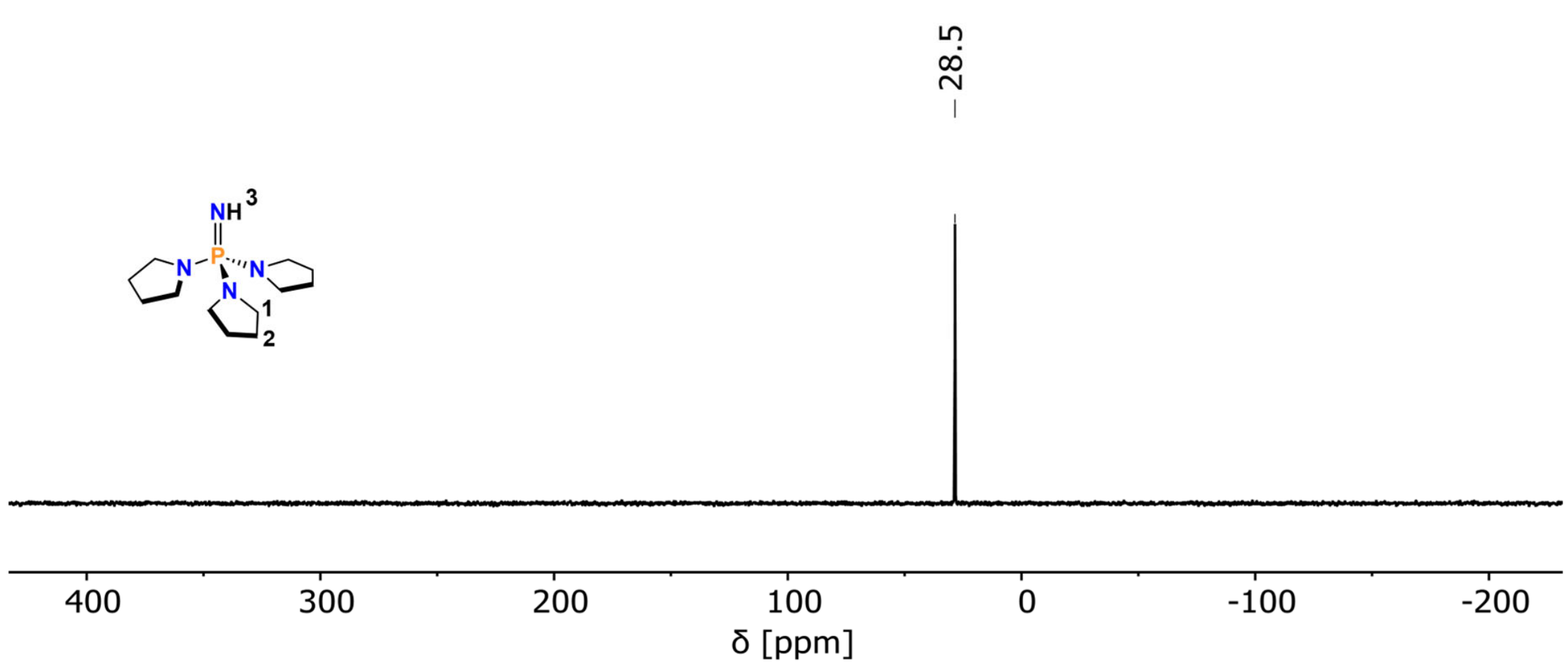

Figure S10: ^{31}P{^{1}H}-NMR spectrum of **HNP^{pyrr}** in C_6D_6.

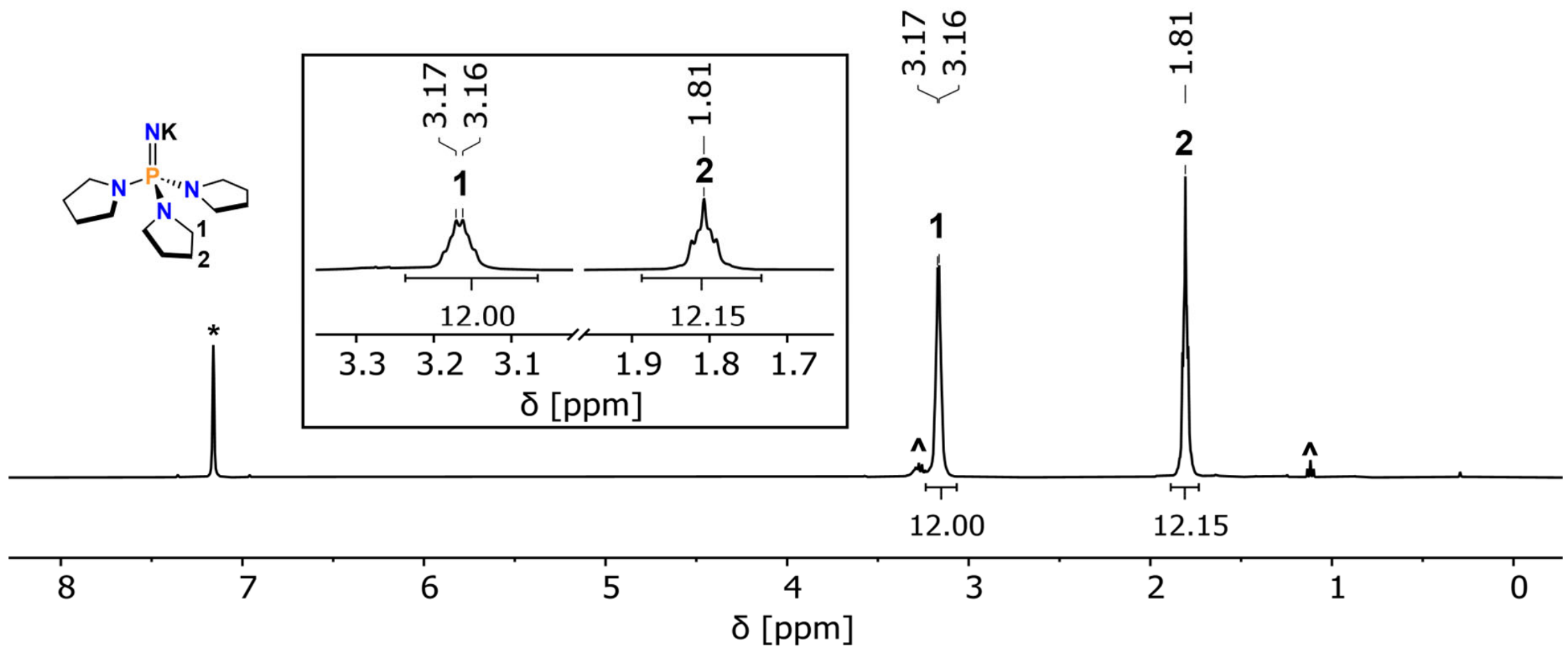

Figure S11: ^{1}H-NMR spectrum of **KNP^{pyrr}** in C_6D_6. C_6D_5H is marked with * and residual diethyl ether with ^.

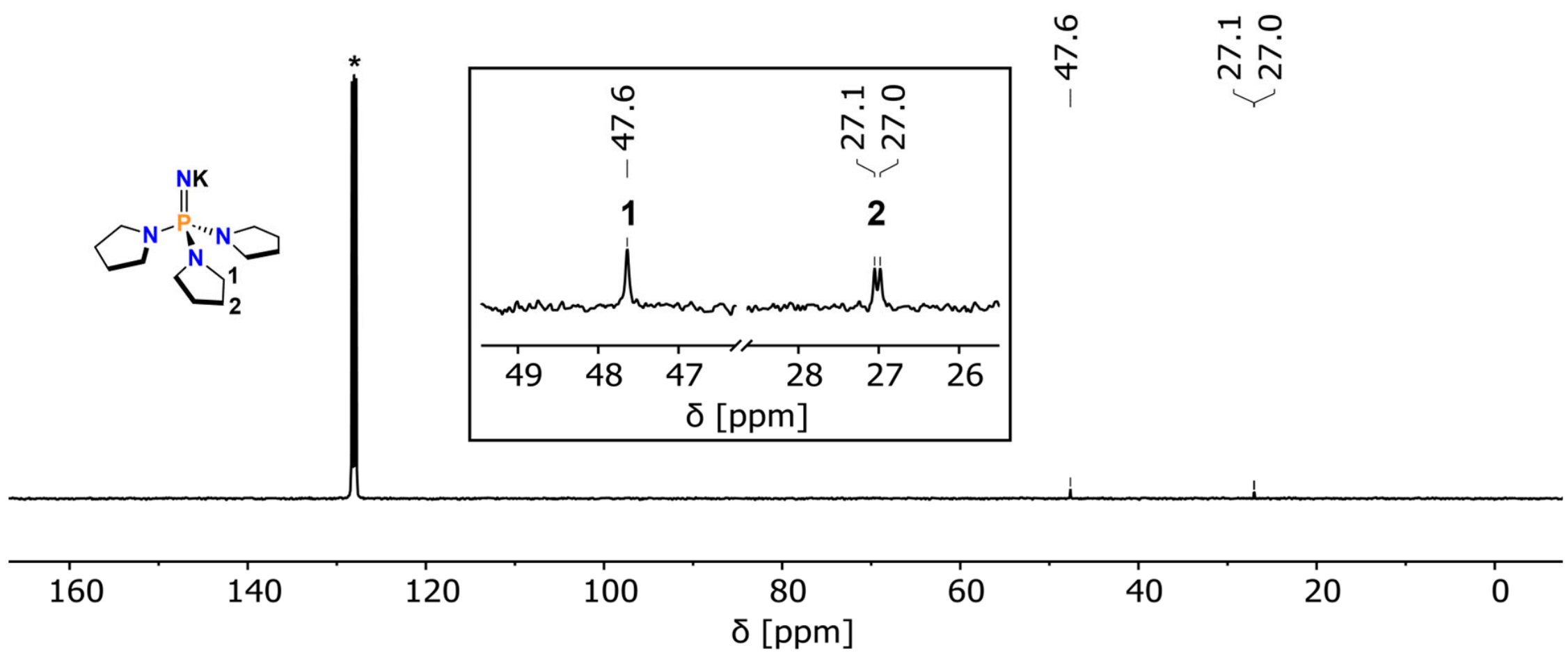

Figure S12: ^{13}C{^{1}H}-NMR spectrum of **KNP^{pyrr}** in C_6D_6. Residual solvent signal is marked with *.

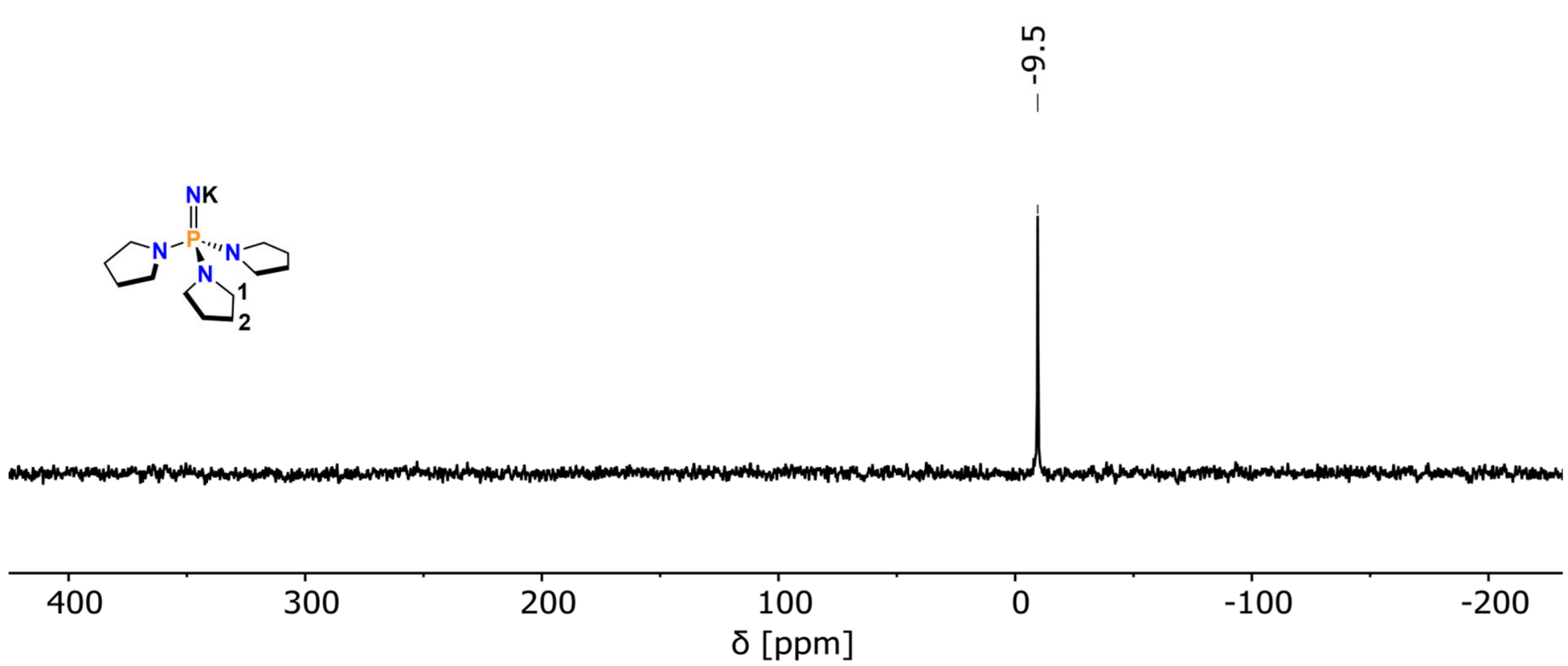

Figure S13: ^{31}P{^{1}H}-NMR spectrum of **KNP^{pyrr}** in C_6D_6.

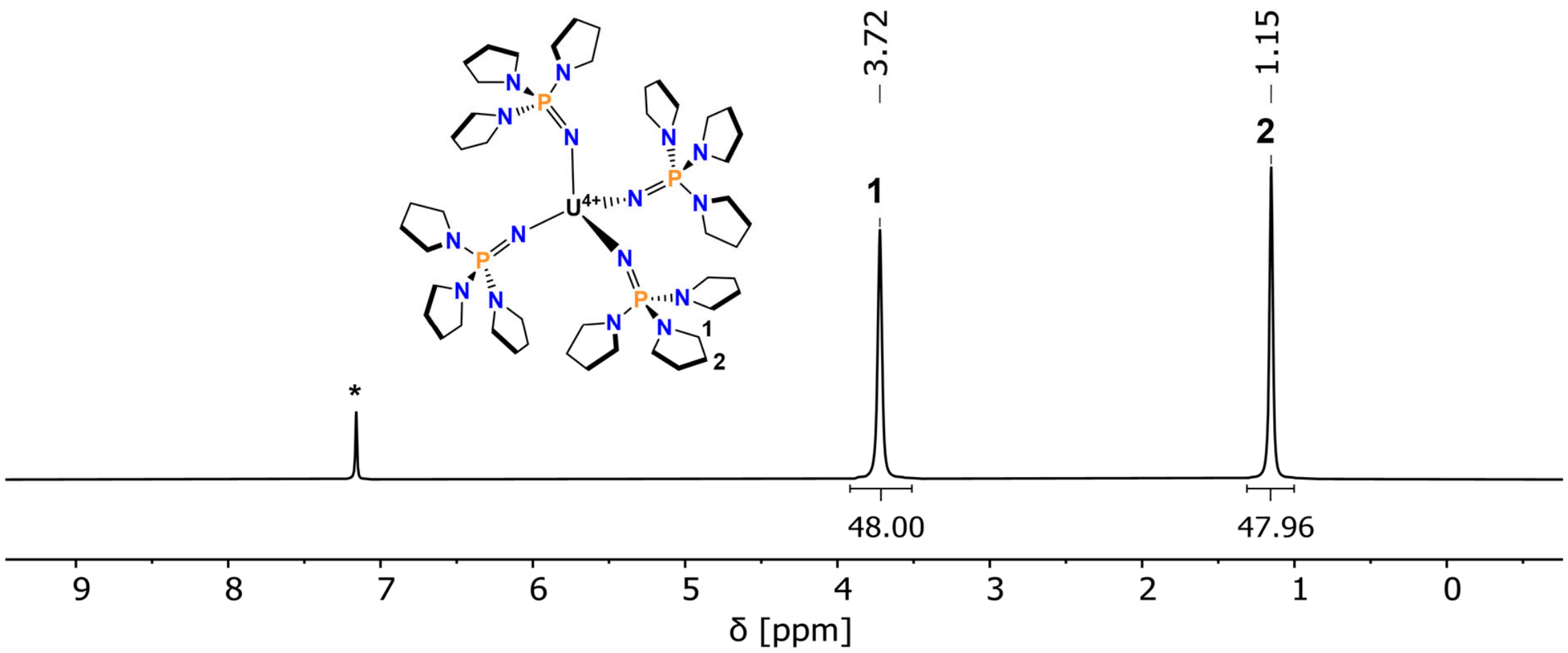

Figure S14: ^{1}H-NMR spectrum of **U(NP^{pyrr})$_4$** in C_6D_6. Residual solvent signal is marked with *.

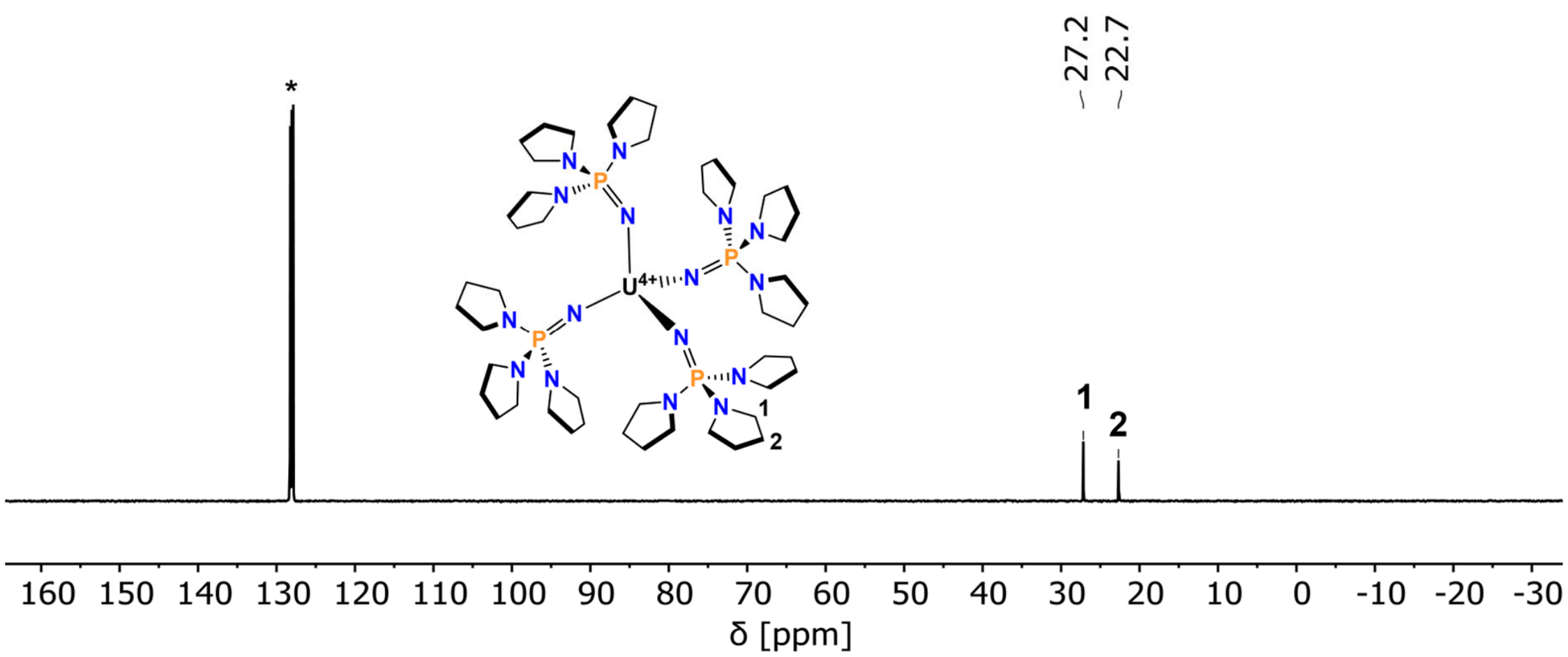

Figure S15: $^{13}C\{^{1}H\}$-NMR spectrum of **U(NP^{pyrr})$_4$** in C_6D_6. Residual solvent signal is marked with *.

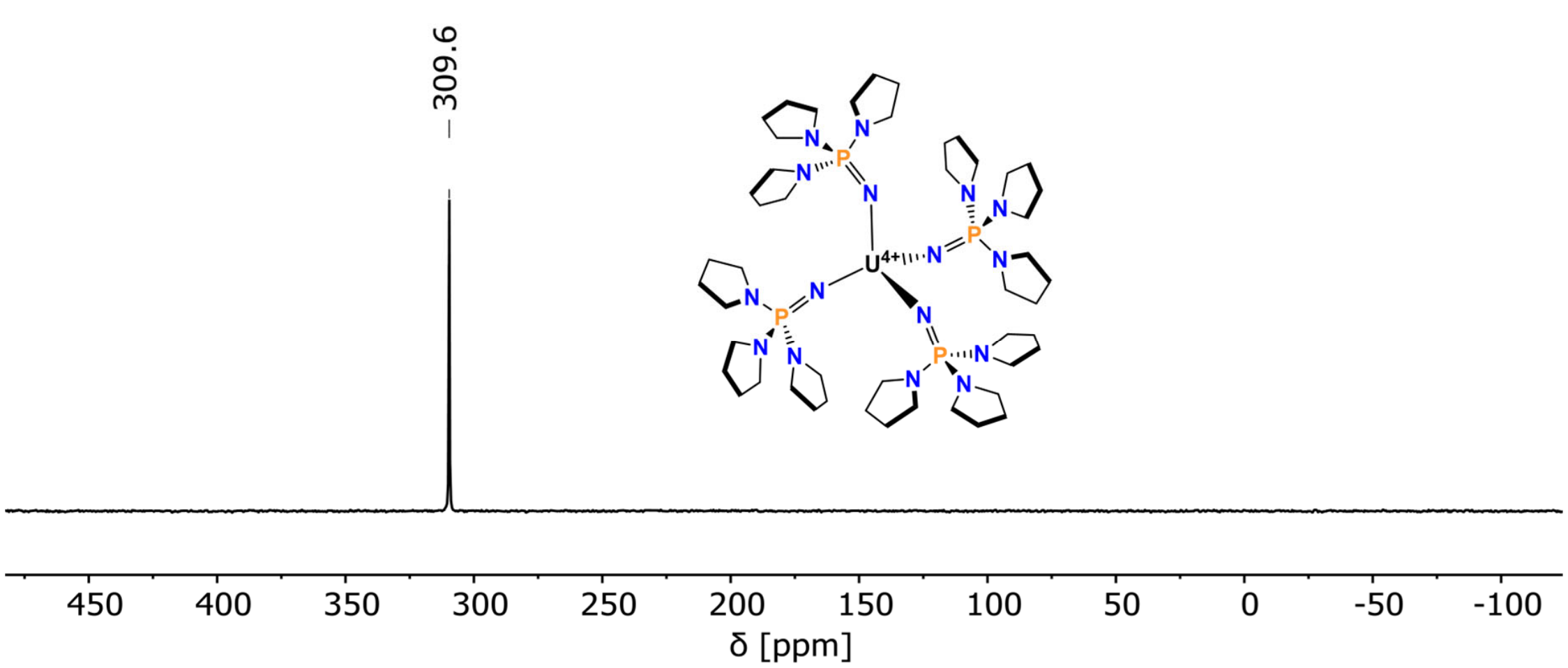

Figure S16: $^{31}P\{^{1}H\}$-NMR spectrum of **U(NP^{pyrr})$_4$** in C_6D_6.

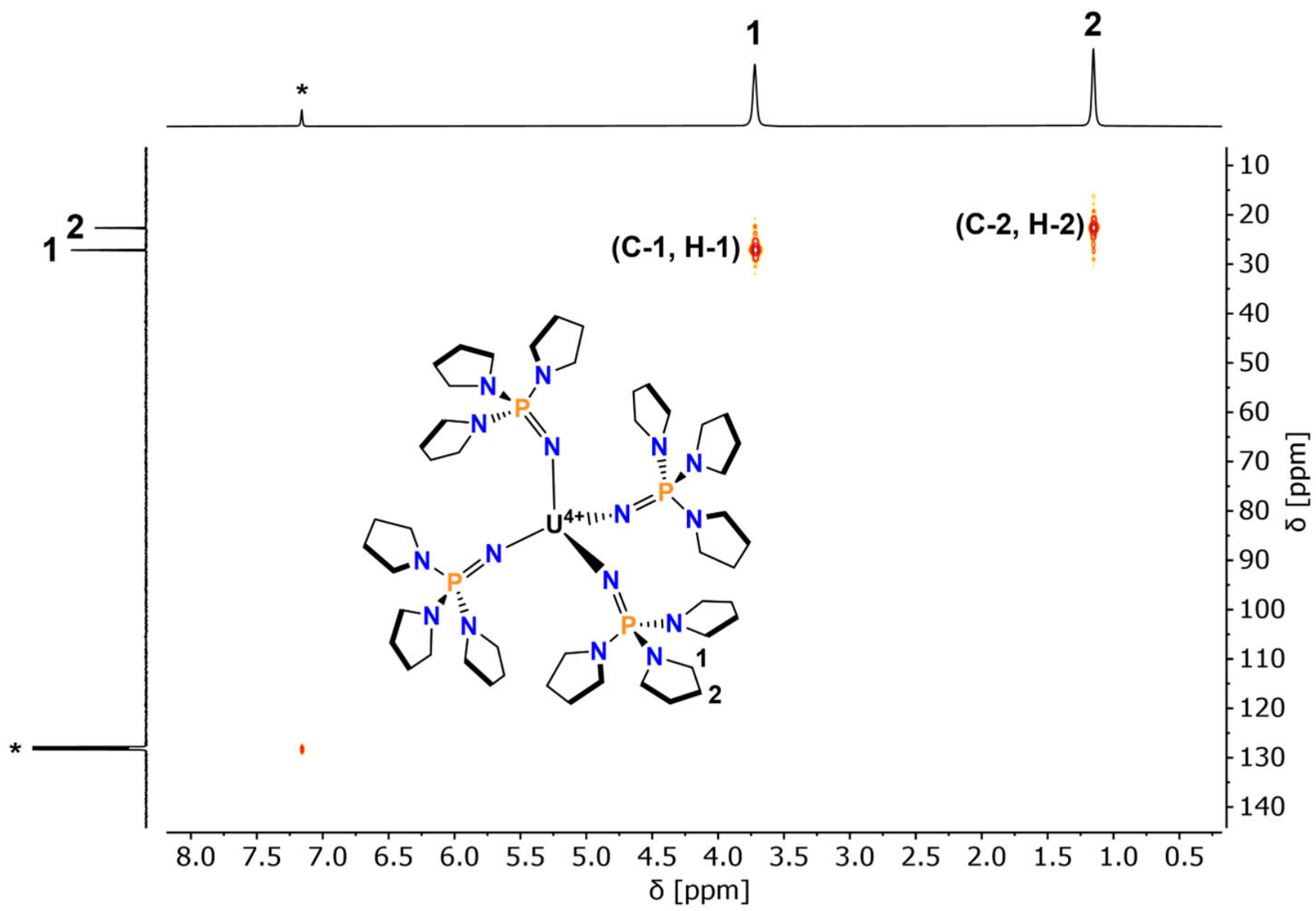

Figure S17: ^{1}H-^{13}C-HSQC-NMR-spectrum of **U(NP^{pyrr})$_4$** in C_6D_6. Residual solvent signals are marked with *.

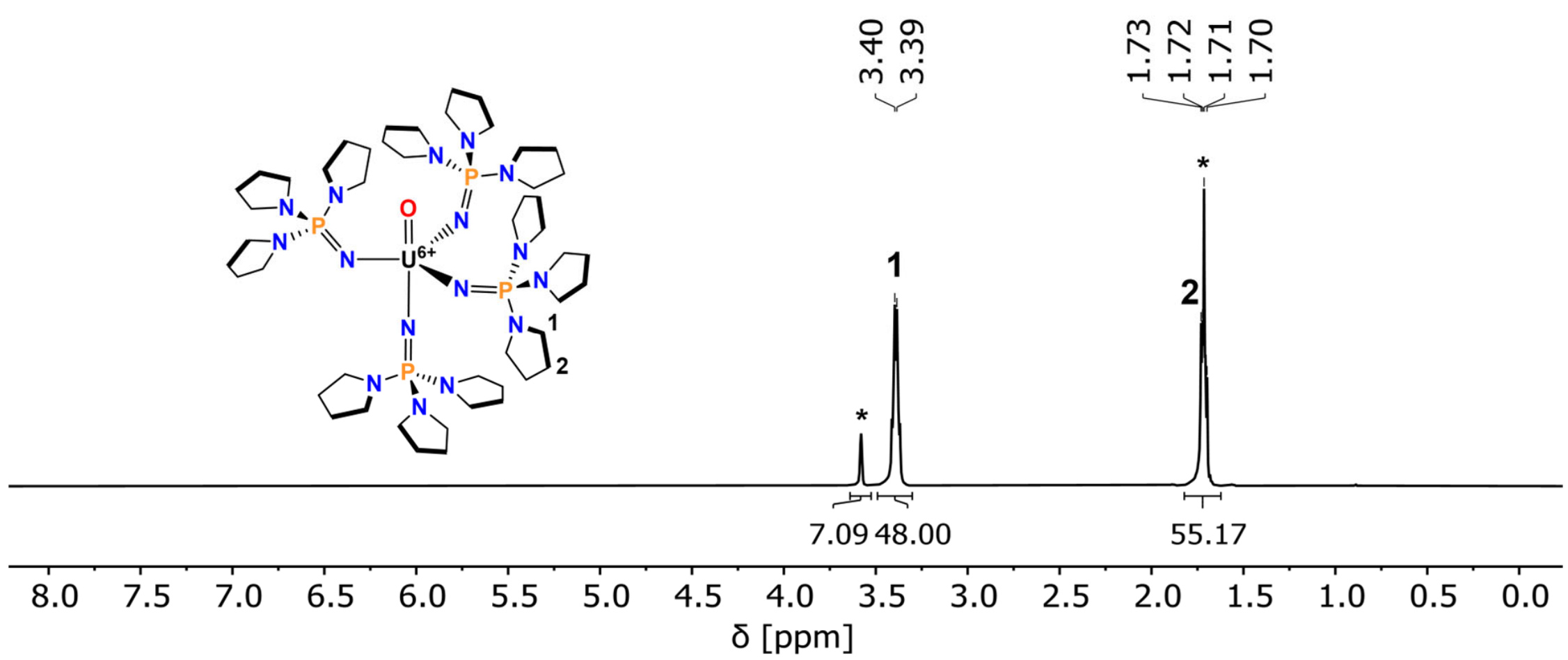

Figure S18: ^{1}H-NMR spectrum of **UO-(NP^{pyrr})$_4$** in THF-d_8. Residual solvent signals are marked with *.

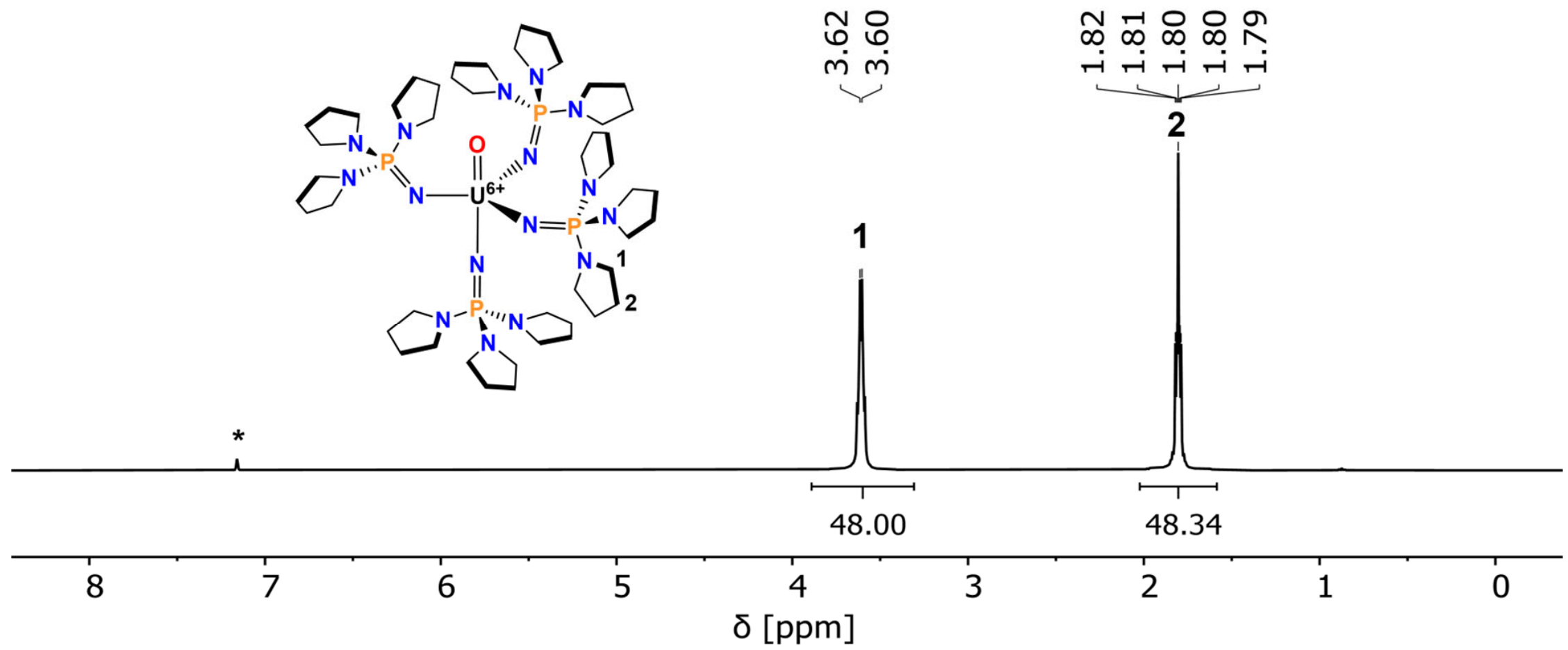

Figure S19: ^{1}H-NMR spectrum of **UO-(NP^{pyrr})$_4$** in C_6D_6. Residual solvent signal is marked with *.

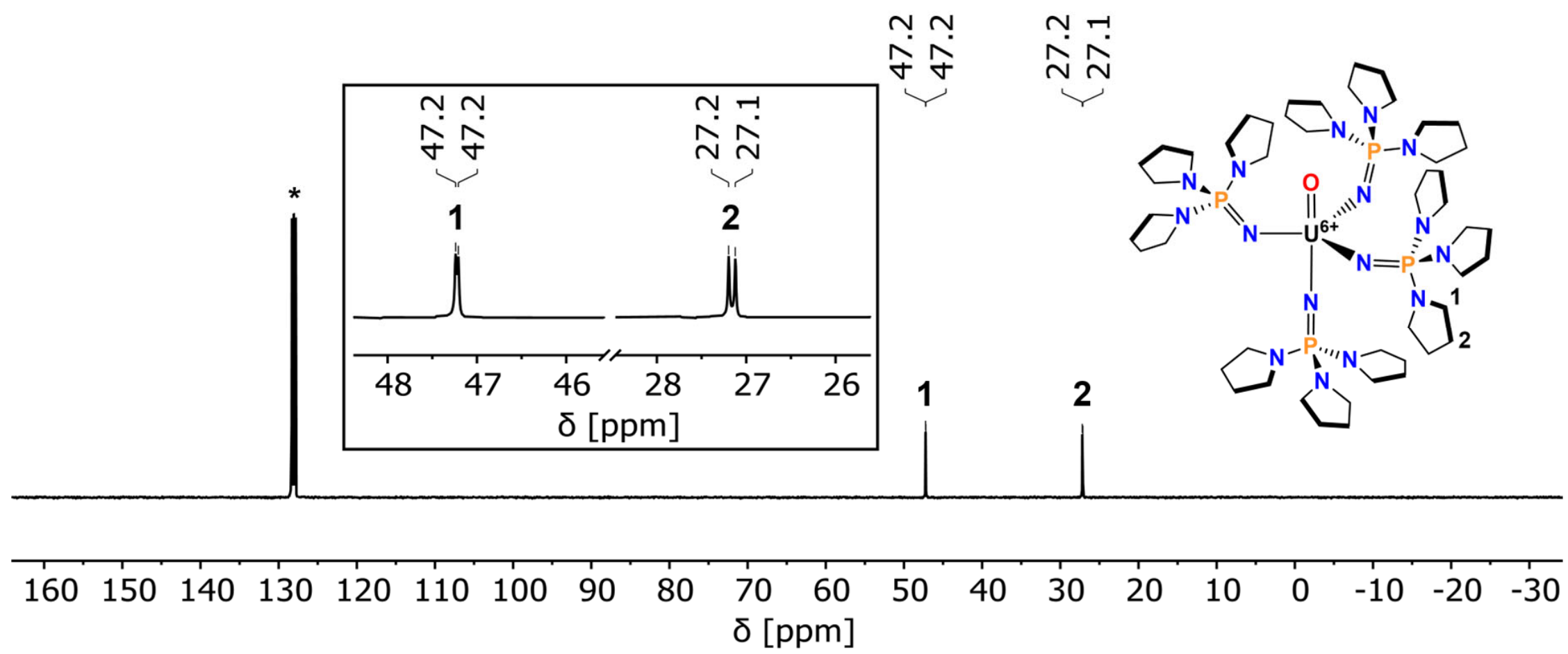

Figure S20: ^{13}C{^{1}H}-NMR spectrum of **UO-(NP^{pyrr})$_4$** in C_6D_6. Residual solvent signal is marked with *.

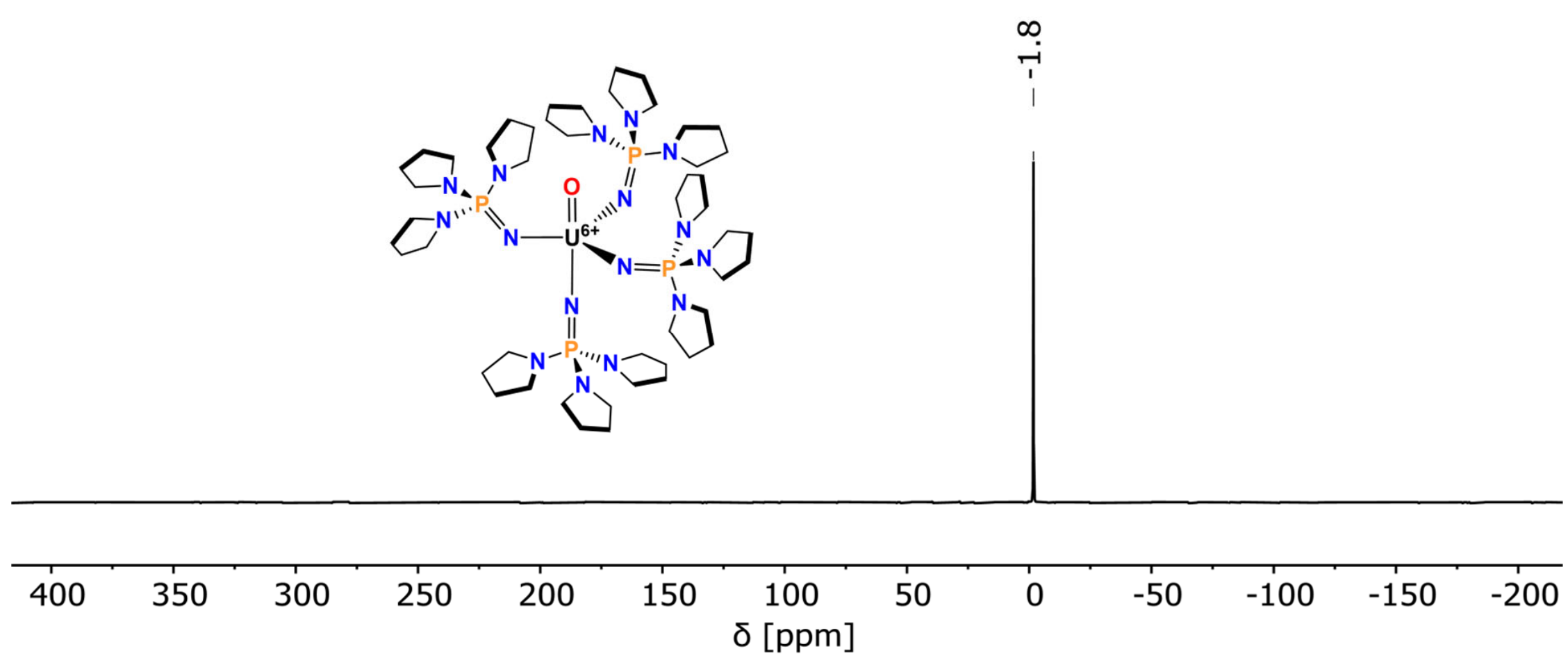

Figure S21: ^{31}P{^{1}H}-NMR spectrum of **UO-(NP^{pyrr})$_4$** in C_6D_6.

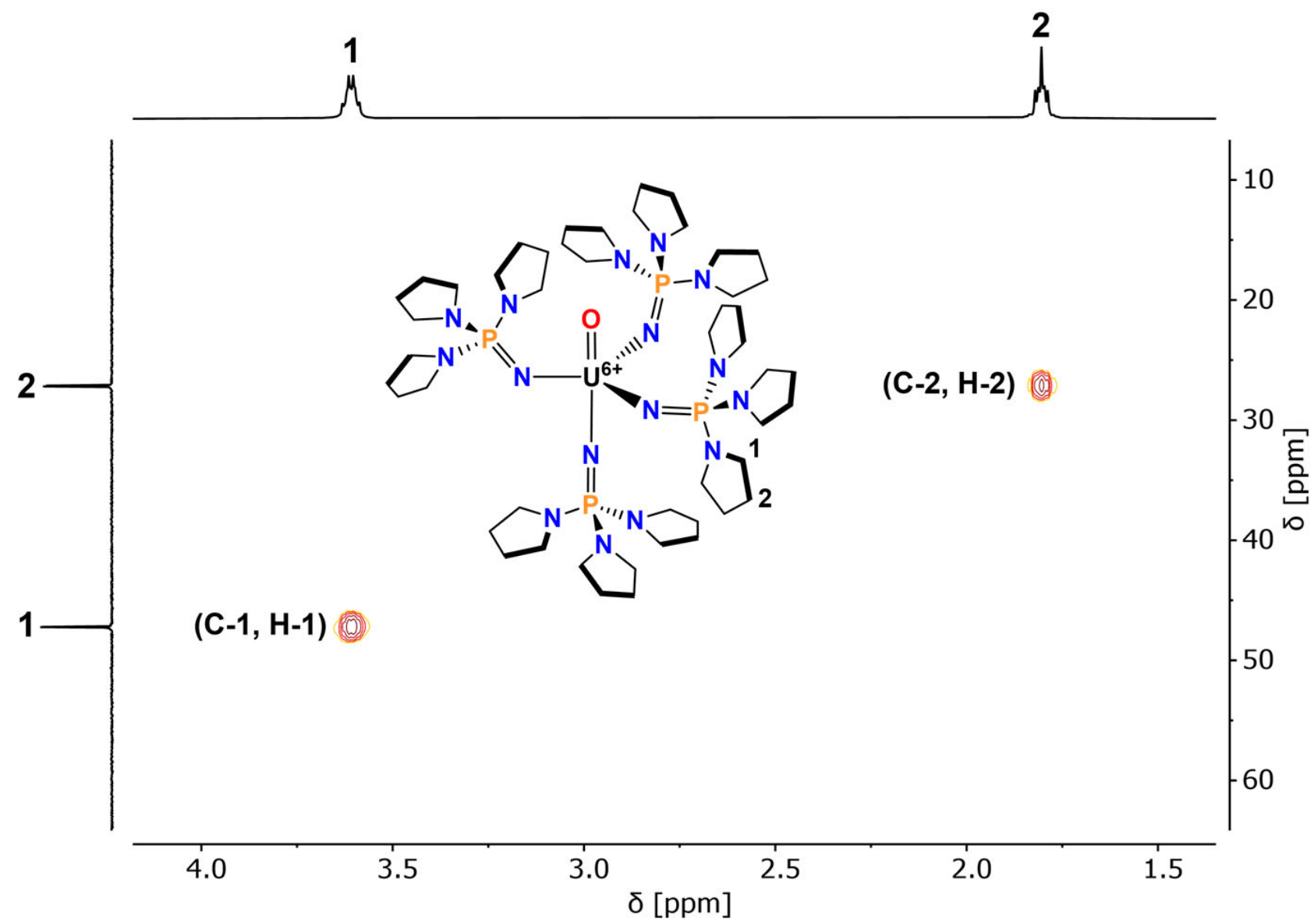

Figure S22: Expansion of ^{1}H-^{13}C-HSQC-NMR-spectrum of **UO-(NP^{pyrr})$_4$** in C_6D_6.

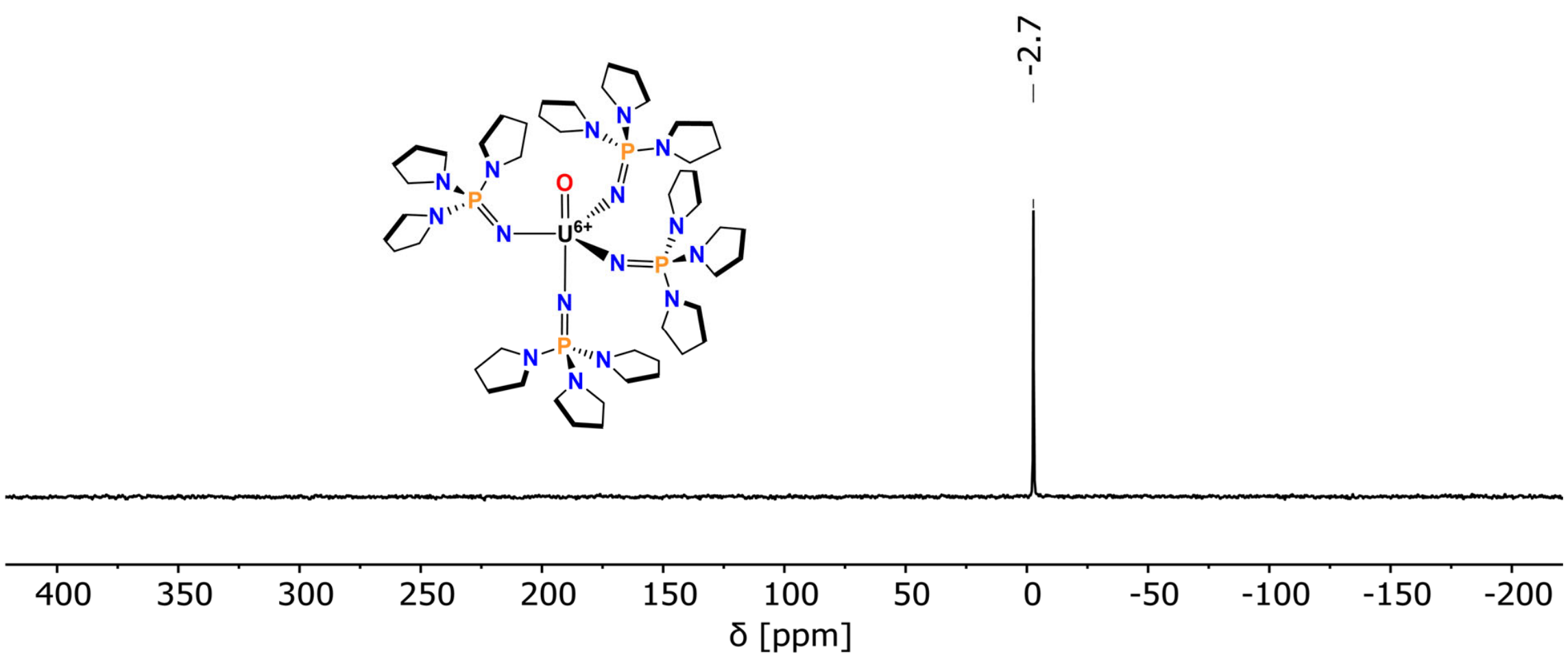

Figure S23: $^{31}P\{^{1}H\}$-NMR spectrum of **UO-(NP^{pyrr})$_4$** in THF-d_8.

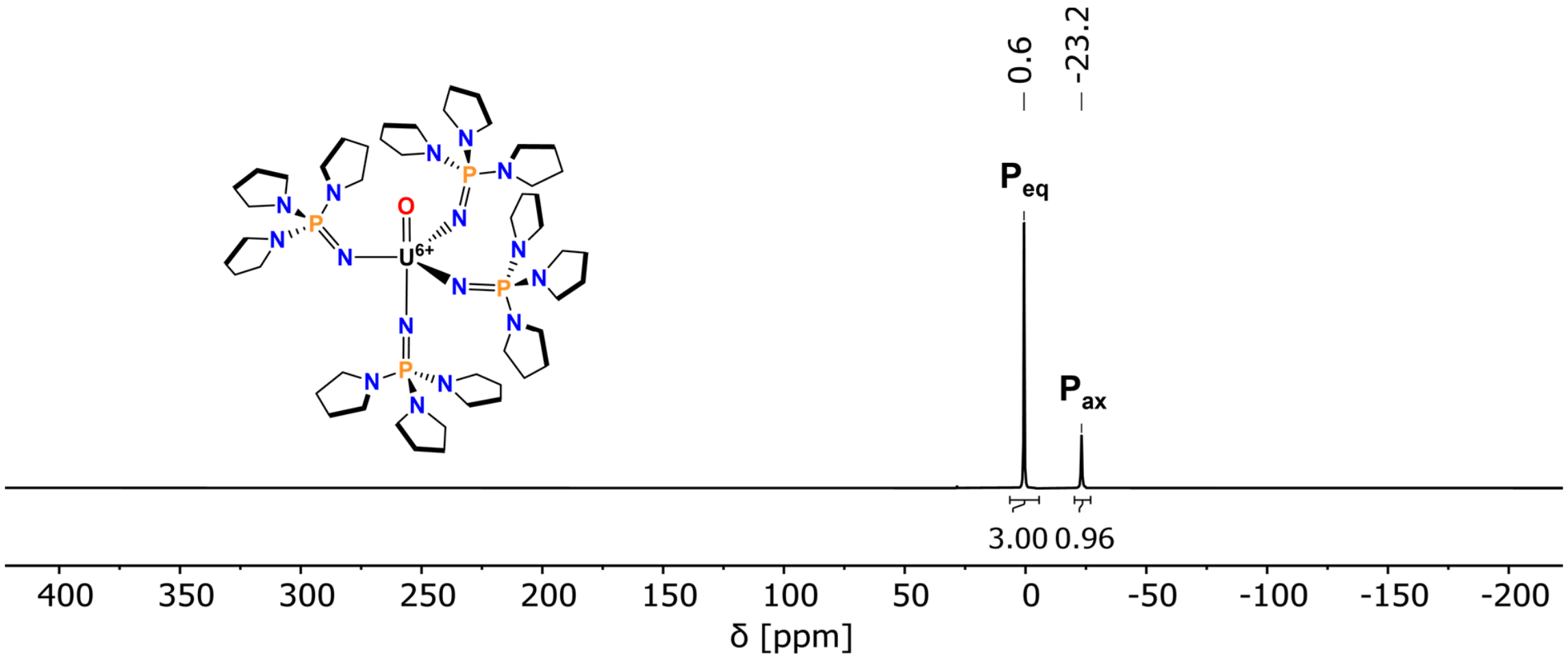

Figure S24: ^{31}P{^{1}H}-NMR spectrum of **UO-(NP^{pyrr})$_4$** in THF-d_8 at –95 °C.

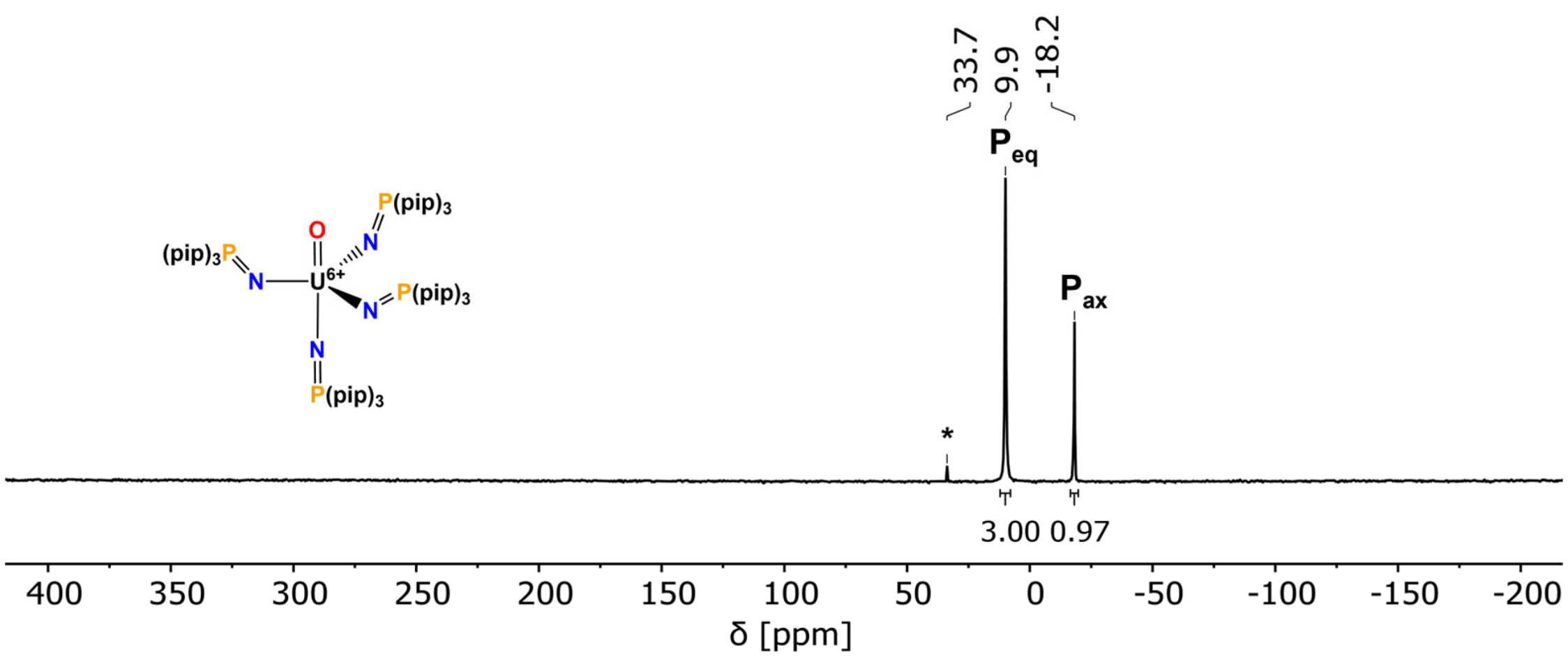

Figure S25: ^{31}P{^{1}H}-NMR spectrum of **UO-(NP^{pip})$_4$** in THF-d_8 at –95 °C. Residual **HNP^{pip}** is marked with *.

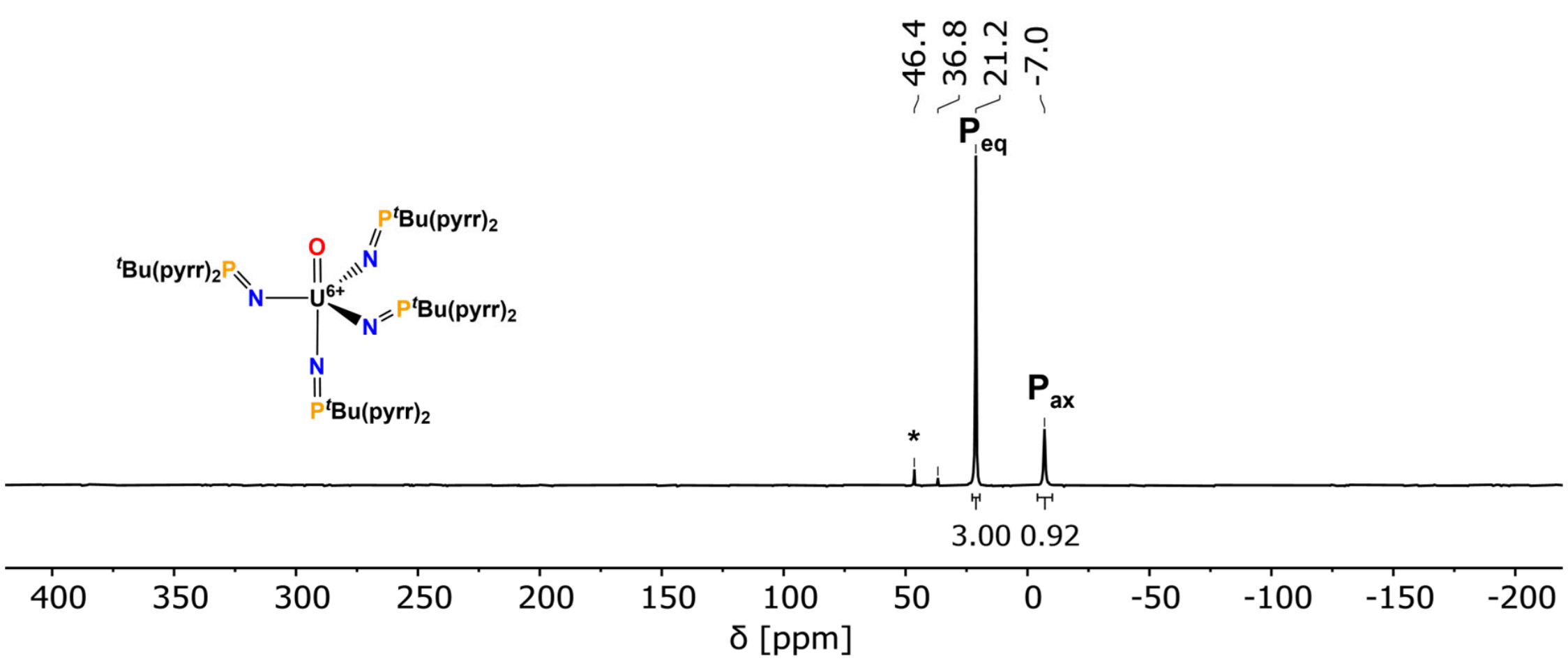

Figure S26: ^{31}P{^{1}H}-NMR spectrum of **UO-(NPC^{1})$_4$** in THF-d_8 at –95 °C. Residual **HNPC^{1}** is marked with *.

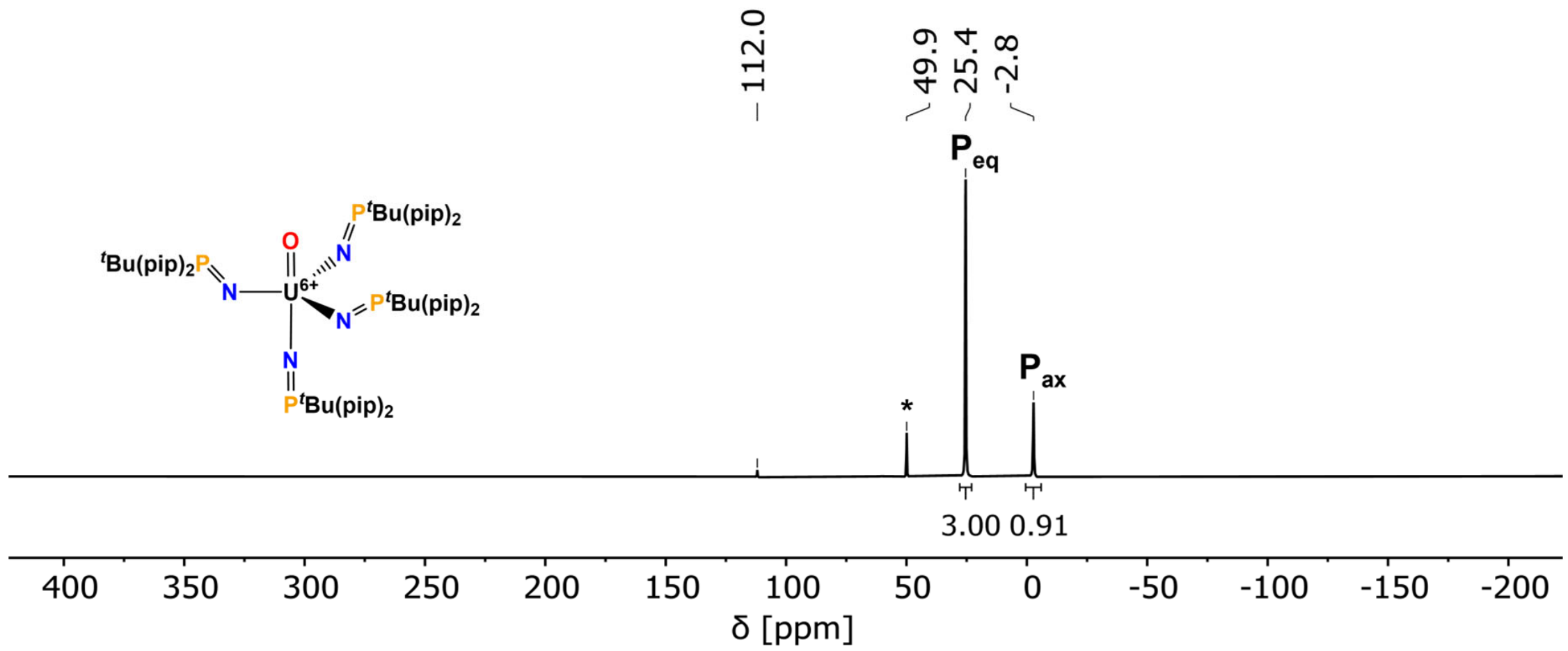

Figure S27: $^{31}P\{^{1}H\}$-NMR spectrum of **UO-(NPC$^{1\text{-pip}}$)$_4$** in THF-d_8 at –95 °C. Residual **HNPC$^{1\text{-pip}}$** is marked with *.

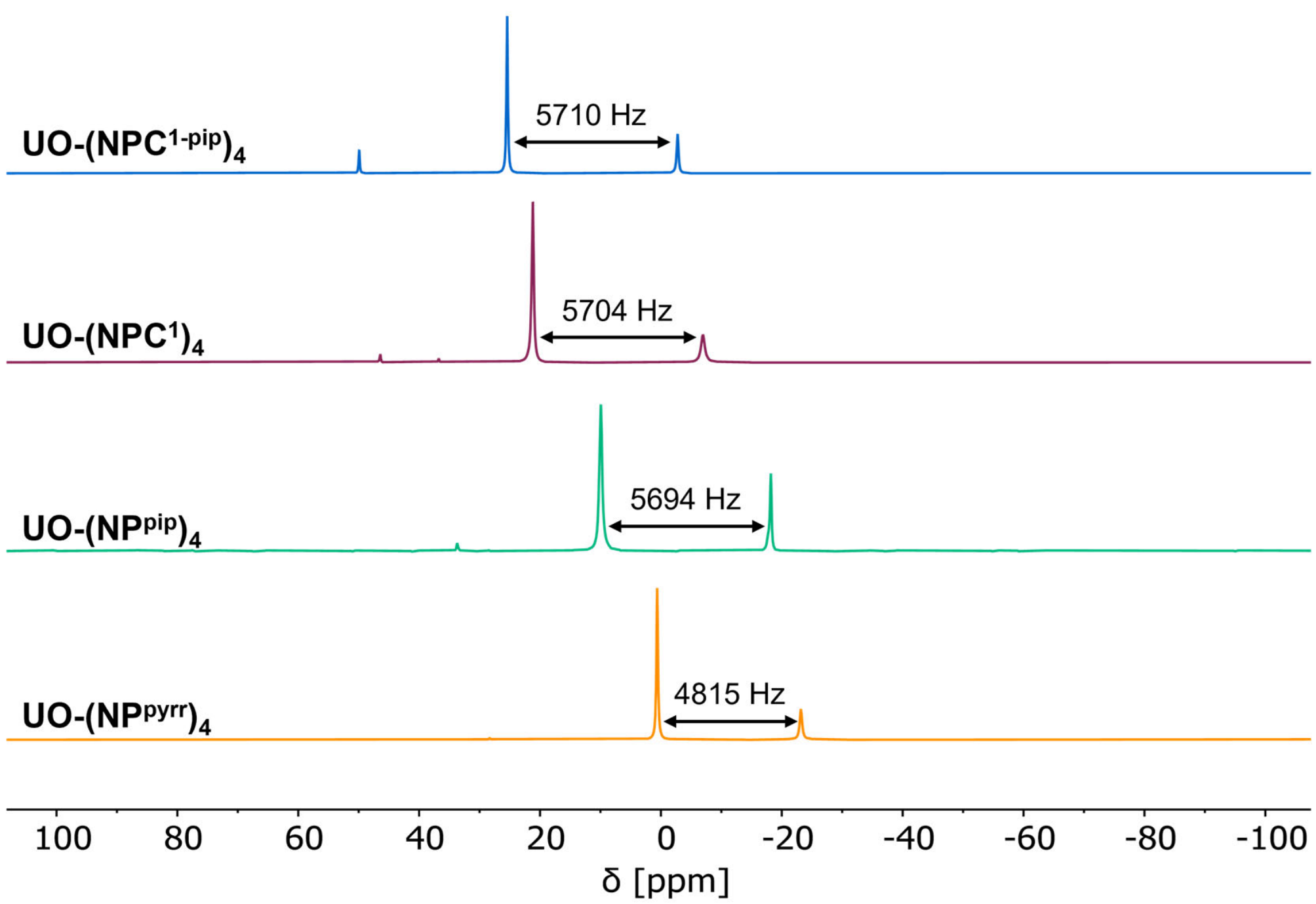

Figure S28: Stack plot of $^{31}P\{^{1}H\}$-NMR spectra of **UO-(NP^{pyrr})$_4$**, **UO-(NP^{pip})$_4$**, **UO-(NPC^{1})$_4$** and **UO-(NPC$^{1\text{-pip}}$)$_4$** at –95 °C in THF-d_8.

3.2 VT and EXSY NMR Analysis

All variable temperature (VT) and exchange spectroscopy (EXSY) NMR spectra were recorded on a 500 MHz Bruker Avance III HD 500 instrument. Temperatures were calibrated with a 100% MeOH standard solution and using the formula:[14]

$$T(\mathrm{K}) = 403 - 29.53\Delta\delta - 23.87(\Delta\delta)^2 \quad (1)$$

describing the relation between the temperature T in K and the shift difference $\Delta\delta$ in ppm between the CH_3 and OH signals. Using the latter method the instrument was calibrated once in 10 K steps from room temperature to –90 °C. Towards lower temperatures higher N_2 gas flows are required to reach the respective temperatures. Using the same gas flows for the different temperatures for all samples allowed to use the same temperature calibration for all measurements without recalibrating the instrument each time. The temperatures for the coalescence temperature and the EXSY spectra were calibrated new for each sample to get most accurate values for the $\Delta G^\ddagger$ calculations. Despite all efforts some uncertainty in the temperature remains, largely due to instrument limited fluctuations of ± 1 K around the respective target temperatures.

All spectra were recorded with a sample concentration of 15.3 mmol/l in THF-d_8.

Variable temperature (VT)-NMR:

The exchange barriers were calculated with an equation from Shanan-Atidi and Bar-Eli:[15]

$$\Delta G^\ddagger = RT_c \ln\left[\frac{kT_c}{h\pi\delta_\nu}\left(\frac{X}{1+\Delta P}\right)\right] \quad (2)$$

where R is the ideal gas constant (8.314472 $\frac{J}{Kmol}$), k is the Boltzmann constant (1.380649 · 10^{-23} $\frac{J}{K}$), T_c is the coalescence temperature (T in K at which multiple peak features are no longer observable), h is Planck's constant (6.62607 · 10^{-34} Js), δ_ν is the peak separation (Hz) at the low-temperature limit, and ΔP is corresponding to the difference in the relative concentration of the two exchanging sites (for a 3:1 ratio $\Delta P = 0.75 - 0.25 = 0.5$). The values for X are dependent of ΔP and were tabulated by Egan.[16] For $\Delta P = 0.5$, $X = 2.3008$. T_c can be obtained by collecting several spectra at different temperatures. At the temperature where the one signal split into two that is the coalescence temperature.

Spectra were recorded in 10 K steps starting at –30 °C decreasing to –95 °C and in 1-2 K steps around the respective coalescence temperature. To minimize potential decomposition of the complexes in THF-d_8, spectra at temperatures above –30 °C were measured after the low temperature data had been collected. δ_ν values were measured from spectra collected at –95 °C, the lowest achievable temperature with that instrument and setup.

EXSY-NMR:

T_1 relaxation times (necessary for delay time determination and $\Delta G^\ddagger$ calculations) for the ^{31}P-NMR signals of **UO-(NP^{pyrr})$_4$** at –95 °C were determined with T_1 inversion recovery experiments using a series of τ values. The signals were integrated and normalized to the maximum value.

Table S1: Integrations and normalized integrations of the P_{eq} (0.6 ppm) and P_{ax} (–23.2 ppm) signals of **UO-(NP^{pyrr})$_4$** at –95 °C in ^{31}P T_1 inversion recovery experiments for a series of τ values.

τ [s]	integral for P_{eq} (0.6 ppm) [a.u.]	integral for P_{ax} (–23.2 ppm) [a.u.]	normalized integral P_{eq} (0.6 ppm) [a.u.]	normalized integral P_{ax} (–23.2 ppm) [a.u.]
**0.001**	–1.2377E9	–4.1021E8	–0.87955	–0.88734
**0.050**	–1.0743E9	–3.4729E8	–0.76343	–0.75124
**0.100**	–9.1167E8	–3.1314E8	–0.64786	–0.67737
**0.200**	–6.2055E8	–2.0306E8	–0.44098	–0.43925
**0.300**	–3.6504E8	–1.3265E8	–0.25941	–0.28694
**0.400**	–1.4859E8	–5.2678E7	–0.10559	–0.11395
**0.500**	4.9363E7	1.2562E7	0.03508	0.02717
**0.750**	4.0669E8	1.4896E8	0.28901	0.32222
**1.000**	7.1472E8	2.3050E8	0.50790	0.49860
**1.250**	9.0867E8	3.0139E8	0.64573	0.65195
**1.500**	1.0506E9	3.4770E8	0.74659	0.75213
**2.000**	1.2162E9	4.0090E8	0.86427	0.86720
**2.500**	1.3113E9	4.3952E8	0.93185	0.95075
**3.000**	1.3638E9	4.6229E8	0.96916	1.00000
**4.000**	1.3962E9	4.5563E8	0.99218	0.98559
**5.000**	1.4072E9	4.4790E8	1.00000	0.96887

The integrations were fit using the following formula (as implemented in Topspin 4.4.0):

$$I(\tau) = I_0 + P \cdot e^{\frac{-\tau}{T_1}} \quad (3)$$

with the Levenberg-Marquardt algorithm in OriginPro 2024[17] resulting in T_1 times of 754(4) ms (0.6 ppm) and 747(14) ms (–23.2 ppm) respectively. Fitting results are represented below in **Figure S29** and **Table S2**.

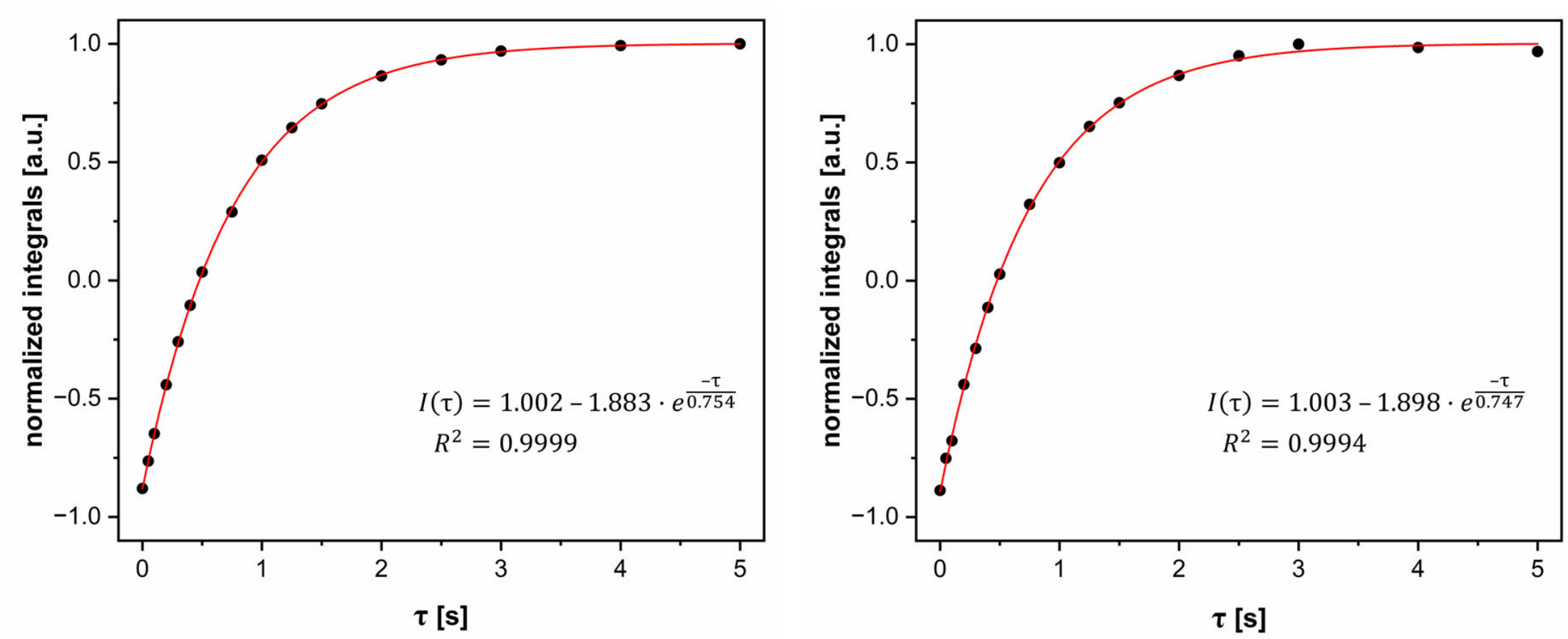

Figure S29: Plots of normalized integrations in dependence of recovery time τ for ^{31}P T_1 inversion recovery experiments of **UO-(NP^{pyrr})$_4$** at –95 °C in THF-d_8. Left: Signal for equatorial P (0.6 ppm) Right: Signal for axial P (–23.2 ppm).

Table S2: Fitting parameters for normalized integrations of the P_{eq} (0.6 ppm) and P_{ax} (–23.2 ppm) signals of **UO-(NP^{pyrr})$_4$** at –95 °C in ^{31}P T_1 inversion recovery experiments in dependence of different τ values.

Fit parameter	P_{eq} (0.6 ppm)	P_{ax} (–23.2 ppm)
T_1	754(4) ms	747(14) ms
I_0	1.002(3)	1.003(9)
P	–1.883(4)	–1.898(13)
R^2	0.9999	0.9994

^{31}P-^{31}P-EXSY-NMR spectra were recorded at –95 °C using the Bruker noesyph pulse sequence in the phase sensitive states-TPPI mode. Per time increment 8 scans per FID of 1024 data points were collected. A total of 128 time increments were collected. Delay times were set to 3.75 s which is ca. 5 times the T_1 relaxation time of the two signals resulting in typical measurement times of ca. 70 min per EXSY experiment. 11 EXSY measurements were repeated with different mixing times (τ_m = 0.3 ms, 1 ms, 3 ms, 5 ms, 7 ms, 10 ms, 30 ms, 50 ms, 100 ms, 200 ms and 500 ms).

The spectra were processed with Topspin 4.4.0. They were manually phase corrected along both dimensions and zero filled to 1024 data points along F1. An exponential window function with 30 Hz line broadening was applied along F2 and a sine bell window function with a sine bell shift of 2 was applied along F1. Signal intensities were obtained by integration in Topspin 4.4.0 and listed in **Table S3**.

Table S3: Integrated intensities (in arbitrary units) of correlation signals in ^{31}P-^{31}P-EXSY-NMR spectra (THF-d_8, –95 °C) of **UO-(NP^{pyrr})$_4$** for different mixing times. AA and BB refer to diagonal peaks and BA and AB to cross peaks. I_c/I_d refers to the ratio of cross and diagonal peak intensity. For I_c only I_{BA} (twice) was used.

τ_m [ms]	I_{AA} [a.u.]	I_{BB} [a.u.]	I_{AB} [a.u.]	I_{BA} [a.u.]	I_c/I_d $I_c = 2I_{BA}$ $I_d = I_{AA}+I_{BB}$	I_{AB}/I_{BA}
**0.3**	145030000	44900000	0	210410	0.002	0.000
**1**	136170000	38746000	1829500	3850900	0.044	0.475
**3**	126550000	28502000	11411000	13789000	0.178	0.828
**5**	115840000	19602000	18604000	21431000	0.316	0.868
**7**	114850000	16897000	22432000	24572000	0.373	0.913
**10**	108680000	13845000	24789000	27474000	0.448	0.902
**30**	103760000	9123000	29322000	31370000	0.556	0.935
**50**	100460000	8889600	28506000	30197000	0.552	0.944
**100**	92754000	8277000	26143000	28089000	0.556	0.931
**200**	81292000	7014800	23316000	24629000	0.558	0.947
**500**	53710000	4845000	14926000	16380000	0.559	0.911

Normalization was achieved by using a ratio of cross peak to diagonal peak intensities (I_c/I_d) instead of individual intensities. Theoretically both cross peaks should have the same intensity fulfilling the criterion $I_{AB}/I_{BA} = 1$. For mixing times of 7 ms and longer this criterion is relatively well met but for shorter mixing times it is no longer fulfilled (**Table S3**). The deviation from the ideal case can be ascribed to artefacts deriving from truncated FID's. These artefacts (expressing as ripples mainly along the F1 dimension) are significantly stronger for the AA than for the BB signal (based on higher intensity of AA). Consequently, ripples along the F1 direction for these signals have a bigger impact on AB than on BA ($I_{AB} < I_{BA}$). At small mixing times, due to the inherently smaller cross peak intensities, I_{AB} and I_{BA} are much stronger affected by these truncation artefacts than at longer mixing times. To mitigate that problem, for the internal normalization instead of adding the intensity of both the AB and BA cross signal, I_{AB} was neglected and I_{BA} was added twice, since I_{BA} should represent a more realistic value for the cross peak intensity.

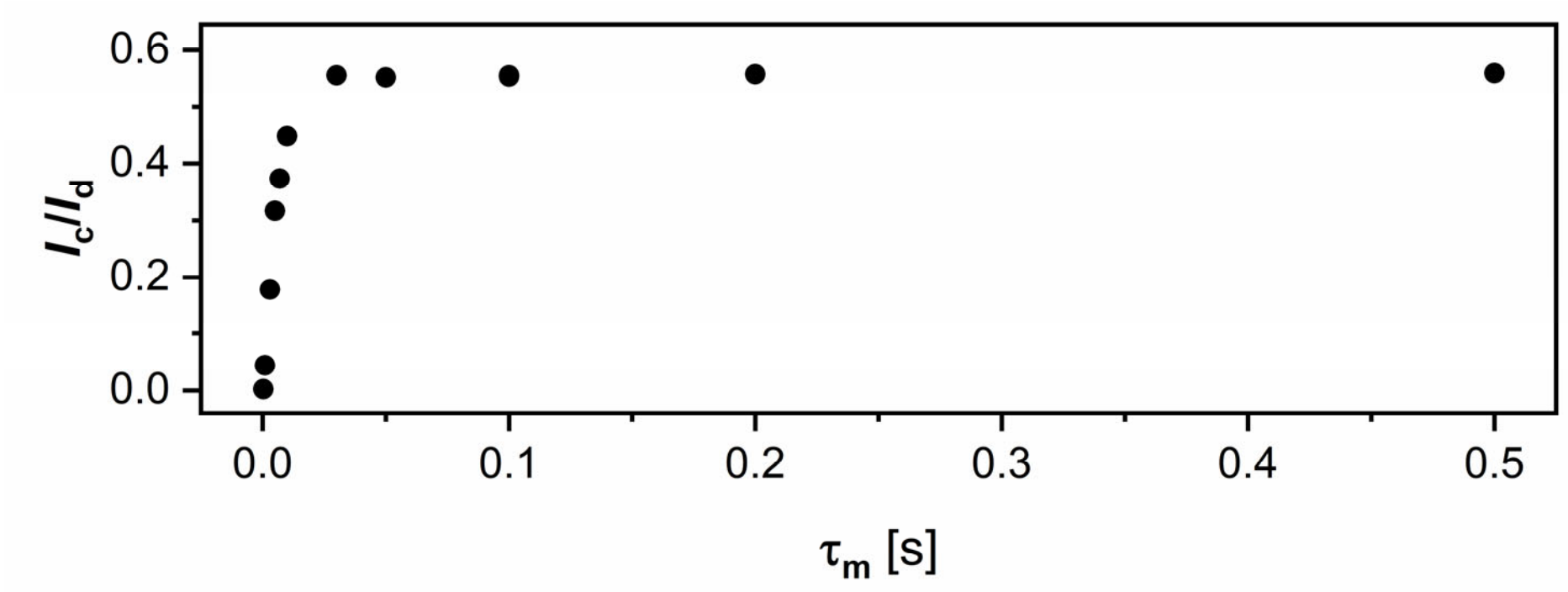

Figure S30: I_c/I_d vs different mixing times (τ_m) of ^{31}P-^{31}P-EXSY-NMR spectra of **UO-(NP^{pyrr})$_4$** at –95 °C in THF-d_8.

The intensity of the correlation signals is dependent on the mixing time τ_m, the exchange rate constant k and relaxation rates R. This relation is described by equations from Jeener et al:[18]

$$I_{AA}(\tau_m) = \chi_A e^{-\sigma\tau_m}\left[\cosh(D\tau_m) - \frac{\delta}{D}\sinh(D\tau_m)\right] \tag{4}$$

$$I_{BB}(\tau_m) = \chi_B e^{-\sigma\tau_m}\left[\cosh(D\tau_m) + \frac{\delta}{D}\sinh(D\tau_m)\right] \tag{5}$$

$$I_{AB}(\tau_m) = I_{BA}(\tau_m) = -\chi_A\chi_B\frac{R_c}{D}e^{-\sigma\tau_m}\sinh(D\tau_m) \tag{6}$$

$$\sigma = \frac{1}{2}(R_{AA} + R_{BB}) \tag{7}$$

$$\delta = \frac{1}{2}(R_{AA} - R_{BB}) \tag{8}$$

$$D = \sqrt{\delta^2 + \chi_A\chi_B{R_c}^2} \tag{9}$$

$$R_{AA} = R_{1A} + 2\chi_B R_D + \chi_B k \tag{10}$$

$$R_{BB} = R_{1B} + 2\chi_A R_D + \chi_A k \tag{11}$$

$$R_c = R_D - k \tag{12}$$

$$R_{1A} = \frac{1}{T_{1A}} \tag{13}$$

$$R_{1B} = \frac{1}{T_{1B}} \tag{14}$$

$$k = k_{AB}\chi_A = k_{BA}\chi_B \tag{15}$$

For a 3:1 ratio ($\chi_A = 0.75, \chi_B = 0.25$), neglecting dipolar cross relaxation ($R_D = 0$) and under the assumption that the T_1 relaxation for both signals is identical ($T_{1A} = T_{1B}$), I_c/I_d described by the equations from Jeener et al. simplifies to the following expression:

$$\frac{I_c}{I_d} = \frac{I_{BA} + I_{BA}}{I_{AA} + I_{BB}} = \frac{3\ \sinh\left(\frac{1}{2}k\tau_m\right)}{4\cosh\left(\frac{1}{2}k\tau_m\right) + \sinh\left(\frac{1}{2}k\tau_m\right)} \tag{16}$$

The experimental I_c/I_d values were modeled with **Equation (16)** with the Levenberg-Marquardt algorithm in the program Origin 2024[17] to give an exchange rate constant k of 183(8) s^{-1}.

3.3 Variable Temperature NMR spectra

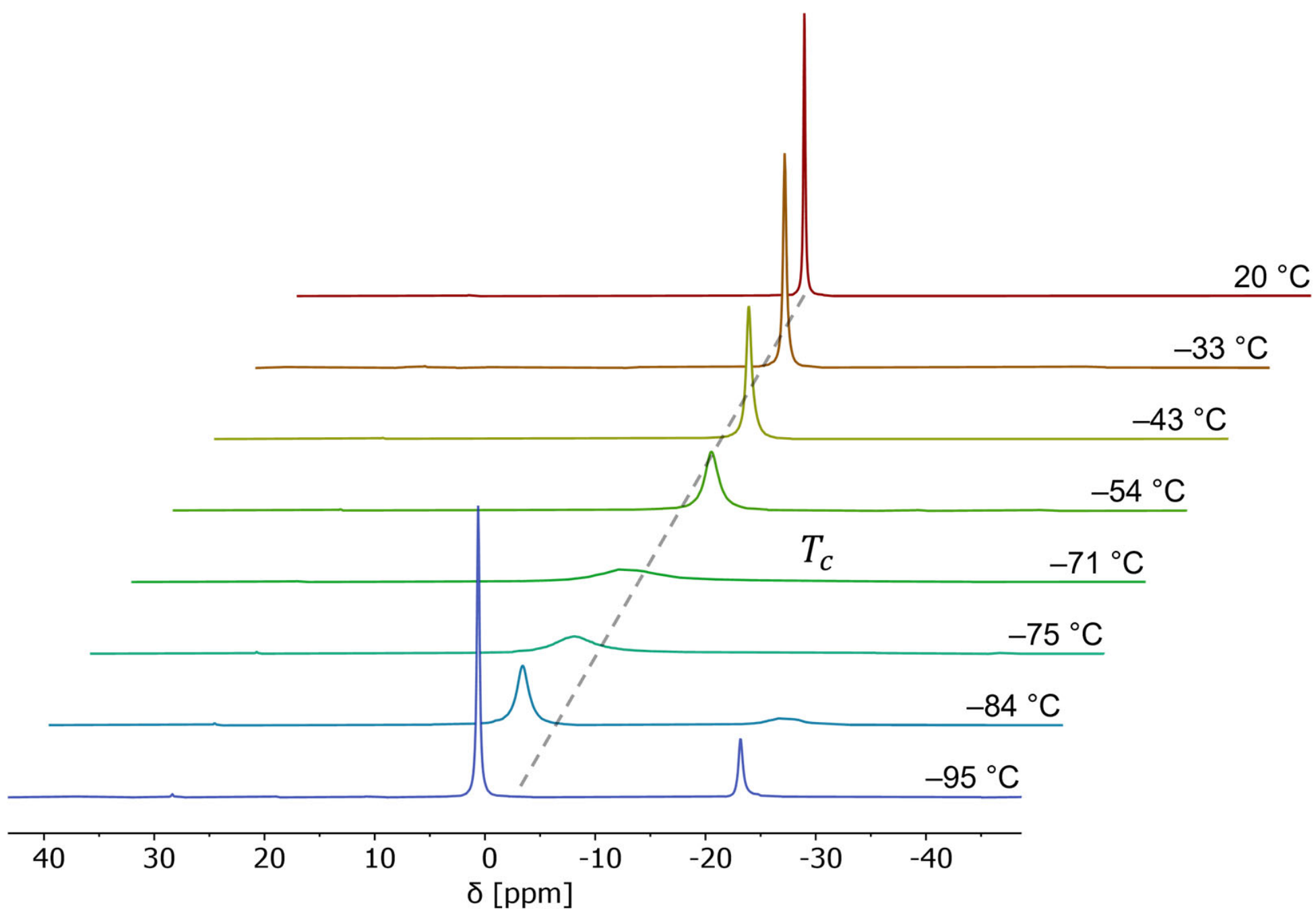

Figure S31: Stack plot (along 30° vector) of VT-^{31}P{^{1}H}-NMR spectra of **UO-(NP^{pyrr})$_4$** between –95 °C (bottom) and 20 °C (top) in THF-*d*$_8$. Depicted is range from 40 to –45 ppm.

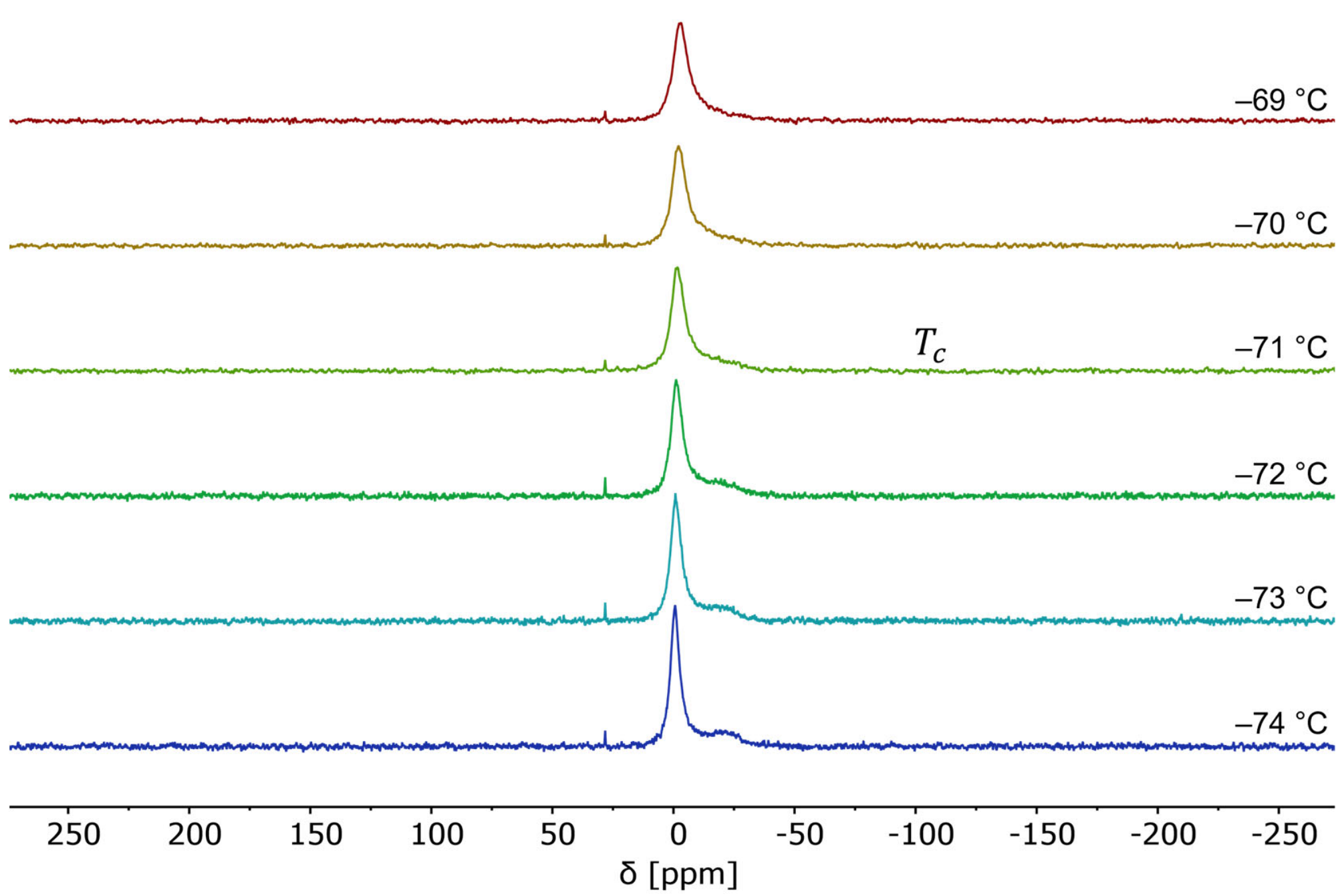

Figure S32: Stack plot of VT-^{31}P{^{1}H}-NMR spectra of **UO-(NP^{pyrr})$_4$** around the coalescence temperature (–71 °C) between –74 °C (bottom) and –69 °C (top) in THF-*d*$_8$. Depicted is range from 250 to –250 ppm. Signal at 28 ppm corresponds to protonated ligand **HNP^{pyrr}**.

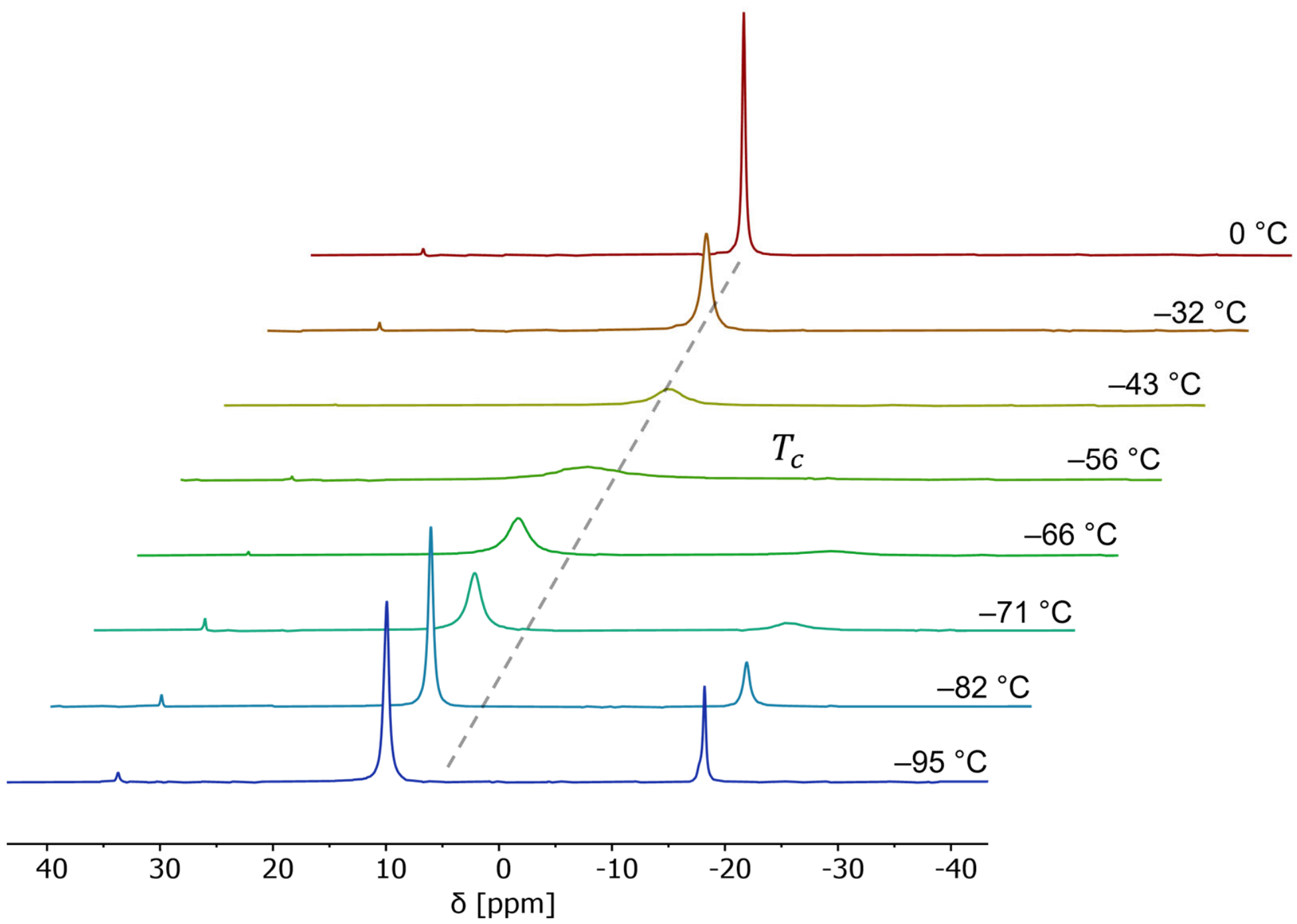

Figure S33: Stack plot (along 30° vector) of VT-$^{31}P\{^1H\}$-NMR spectra of **UO-(NP^{pip})$_4$** between –95 °C (bottom) and 0 °C (top) in THF-d_8. Depicted is range from 40 to –40 ppm. Signal at 33 ppm corresponds to protonated ligand **HNP^{pip}**.

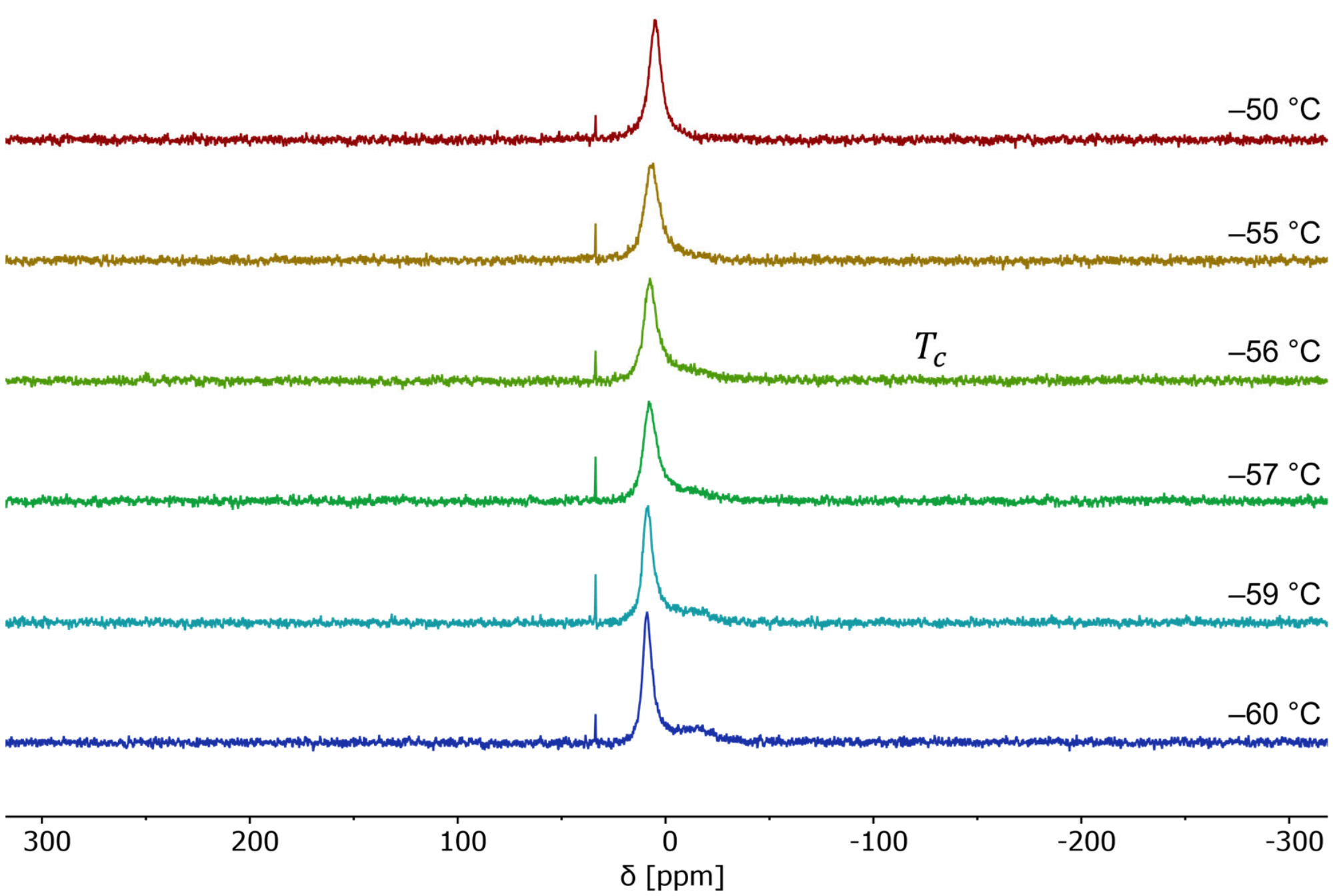

Figure S34: Stack plot of VT-$^{31}P\{^1H\}$-NMR spectra of **UO-(NP^{pip})$_4$** around the coalescence temperature (–56 °C) between –60 °C (bottom) and –50 °C (top) in THF-d_8. Depicted is range from 300 to –300 ppm. Signal at 33 ppm corresponds to protonated ligand **HNP^{pip}**.

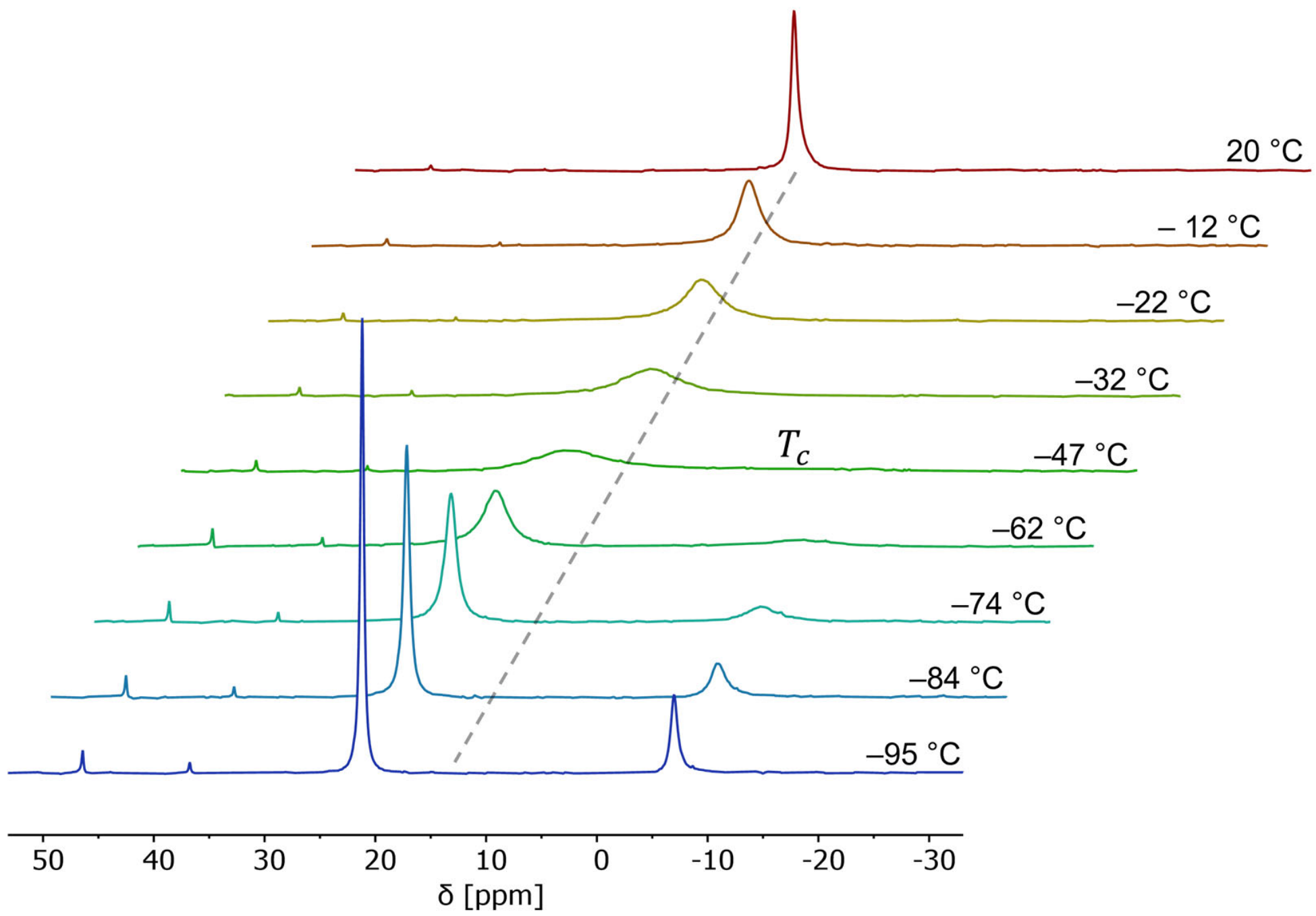

Figure S35: Stack plot (along 30° vector) of VT-^{31}P{^{1}H}-NMR spectra of **UO-(NPC^{1})$_4$** between –95 °C (bottom) and 20 °C (top) in THF-d_8. Depicted is range from 50 to –30 ppm. Signal at 47 ppm corresponds to protonated ligand **HNPC^{1}**, and the signal at 37 ppm was not able to be assigned.

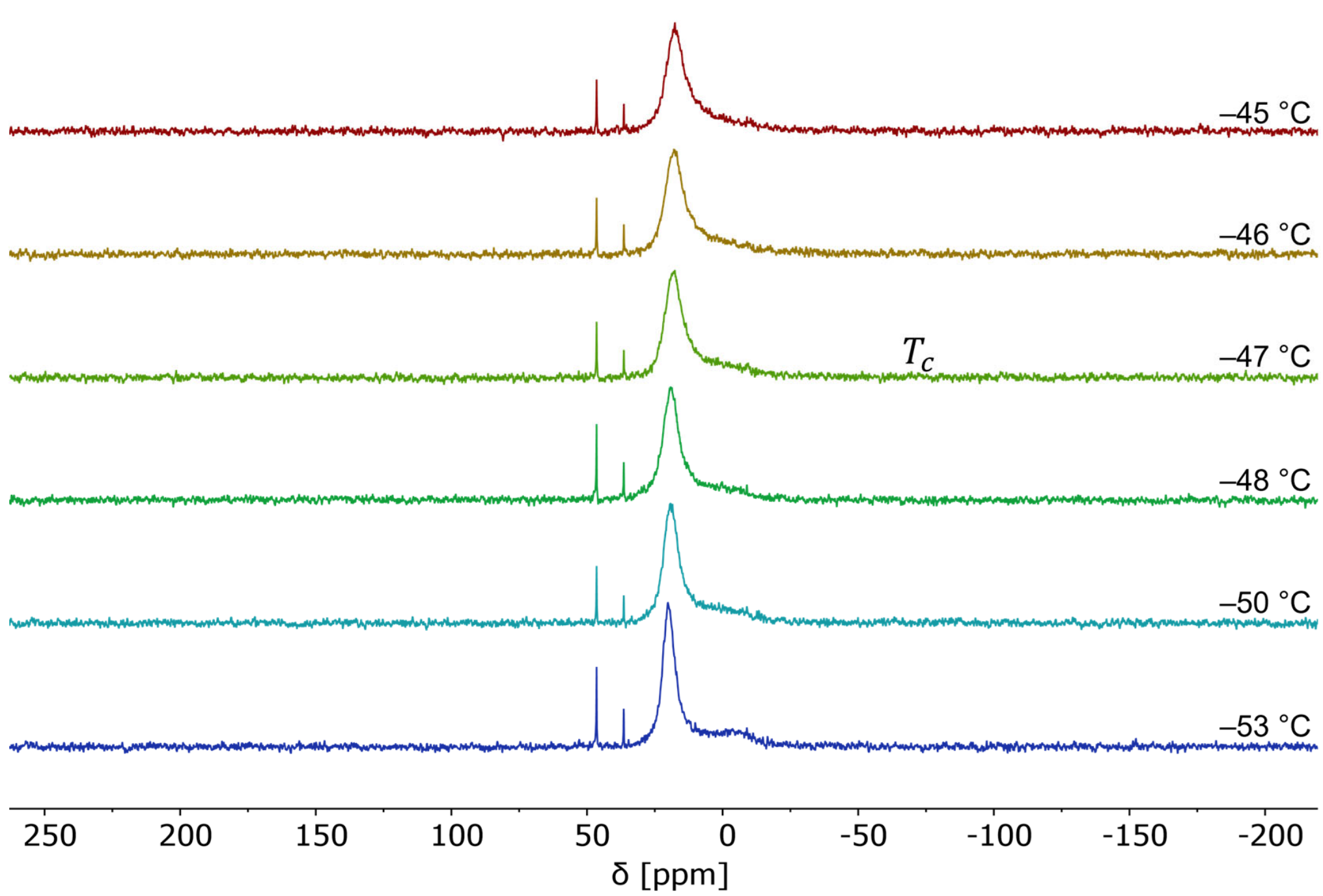

Figure S36: Stack plot of VT-^{31}P{^{1}H}-NMR spectra of **UO-(NPC^{1})$_4$** around the coalescence temperature (–47 °C) between –53 °C (bottom) and –45 °C (top) in THF-d_8. Depicted is range from 250 to –200 ppm. Signal at 47 ppm corresponds to protonated ligand **HNPC^{1}**, and the signal at 37 ppm was not able to be assigned.

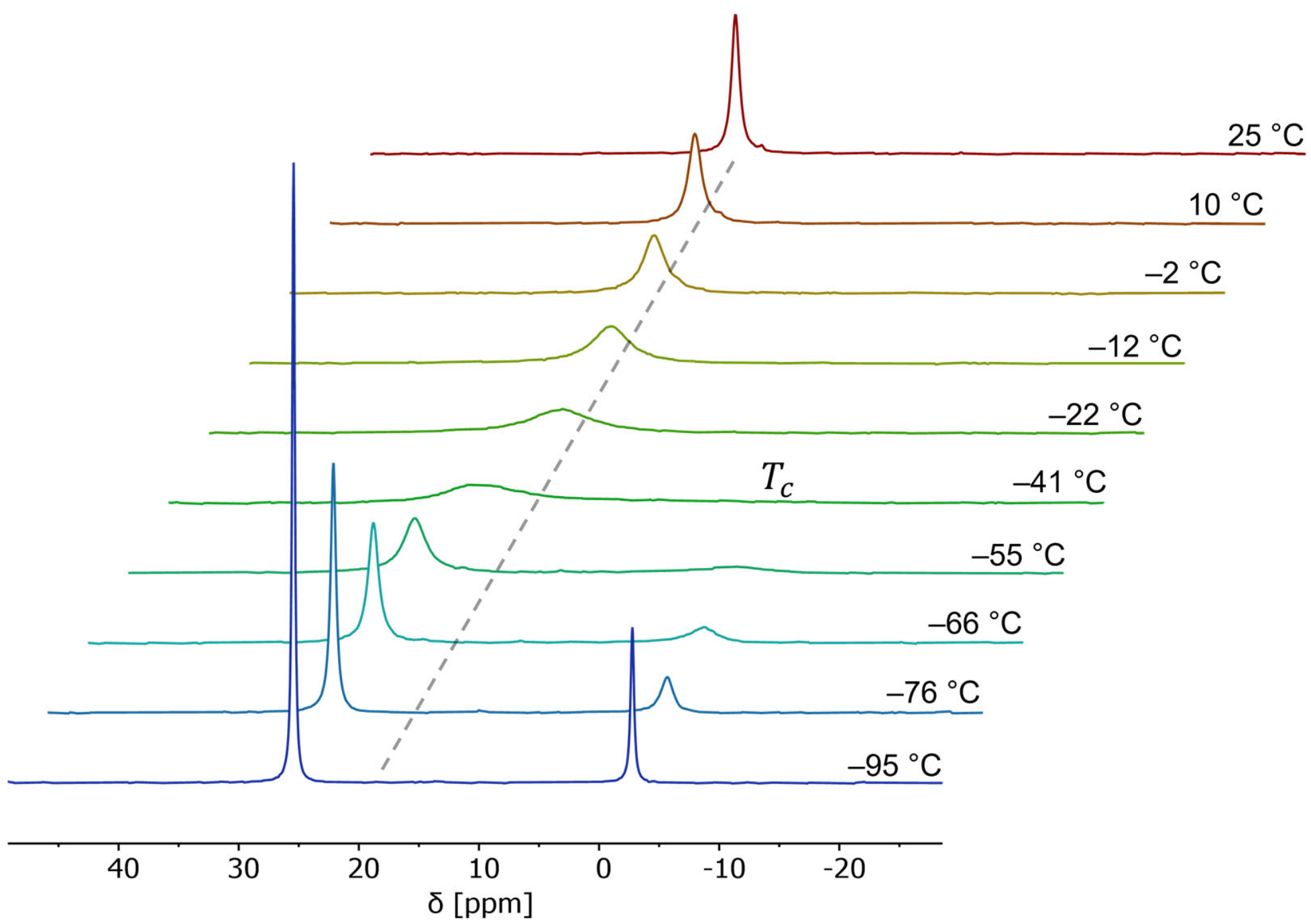

Figure S37: Stack plot (along 30° vector) of VT-$^{31}P\{^1H\}$-NMR spectra of **UO-(NPC^{1-pip})$_4$** between –95 °C (bottom) and 25 °C (top) in THF-d_8. Depicted is range from 45 to –25 ppm.

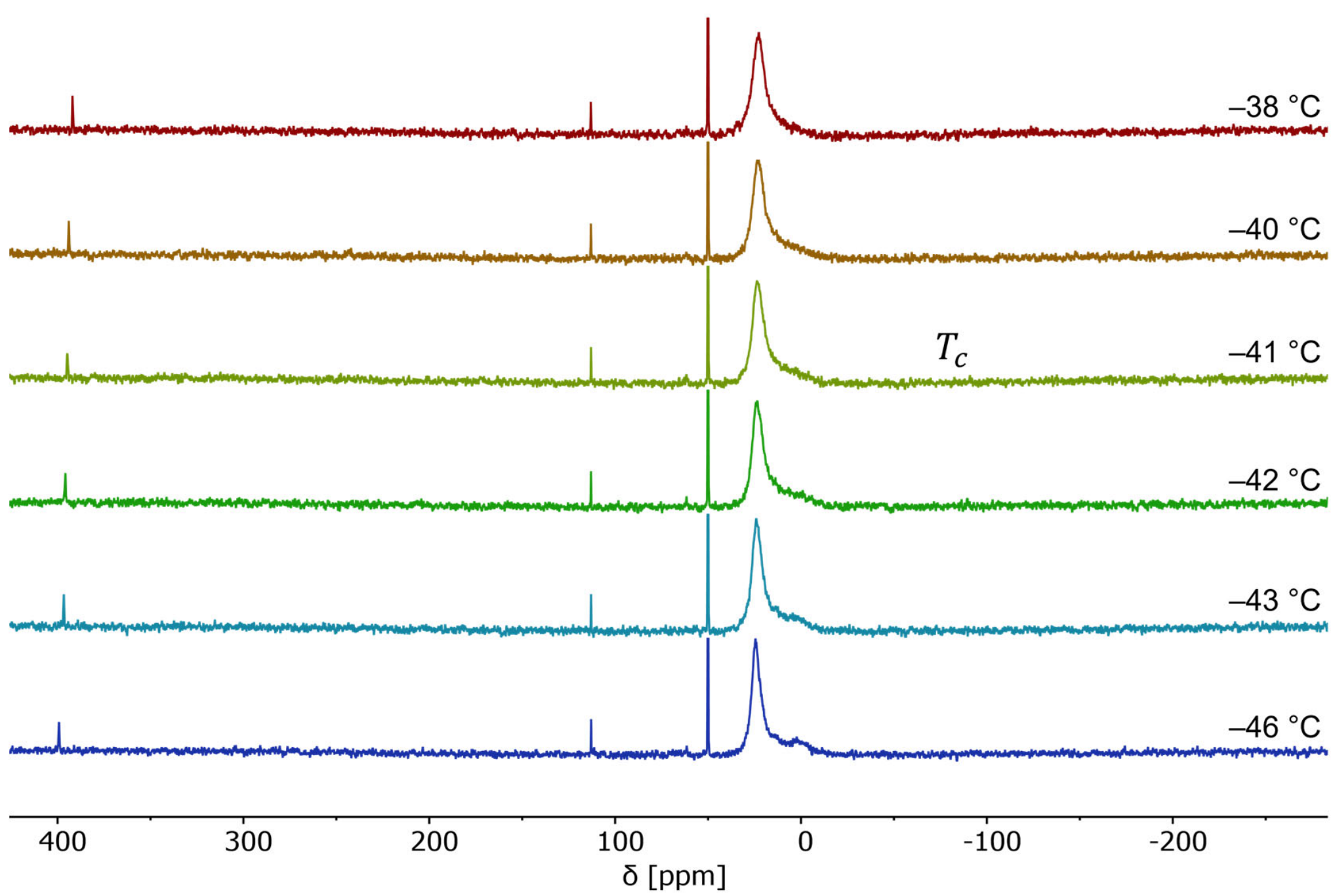

Figure S38: Stack plot of VT-$^{31}P\{^1H\}$-NMR spectra of **UO-(NPC^{1-pip})$_4$** around the coalescence temperature (–41 °C) between –46 °C (bottom) and –38 °C (top) in THF-d_8. Depicted is range from 400 to –250 ppm. Signal at 51 ppm (clipped vertically) corresponds to protonated ligand **HNPC^{1-pip}**, the signal at 394 ppm to **U(NPC^{1-pip})$_4$**, and the signal at 113 ppm was not able to be assigned.

3.4 Exchange Spectroscopy (EXSY) NMR spectra

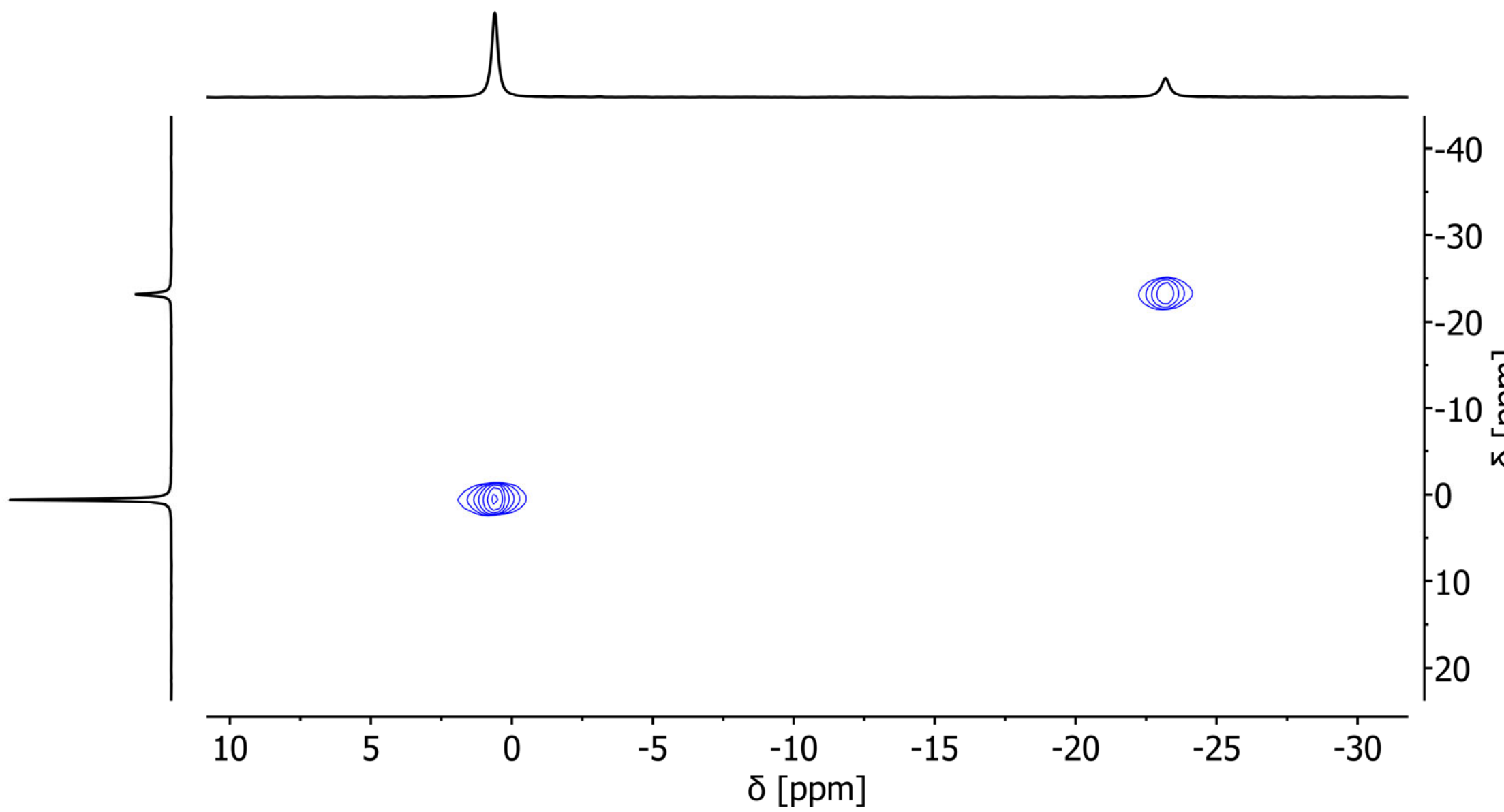

Figure S39: ^{31}P-^{31}P-EXSY-NMR spectrum of **UO-(NP^{pyrr})$_4$** in THF-d_8 at -95 °C with mixing time of 0.3 ms.

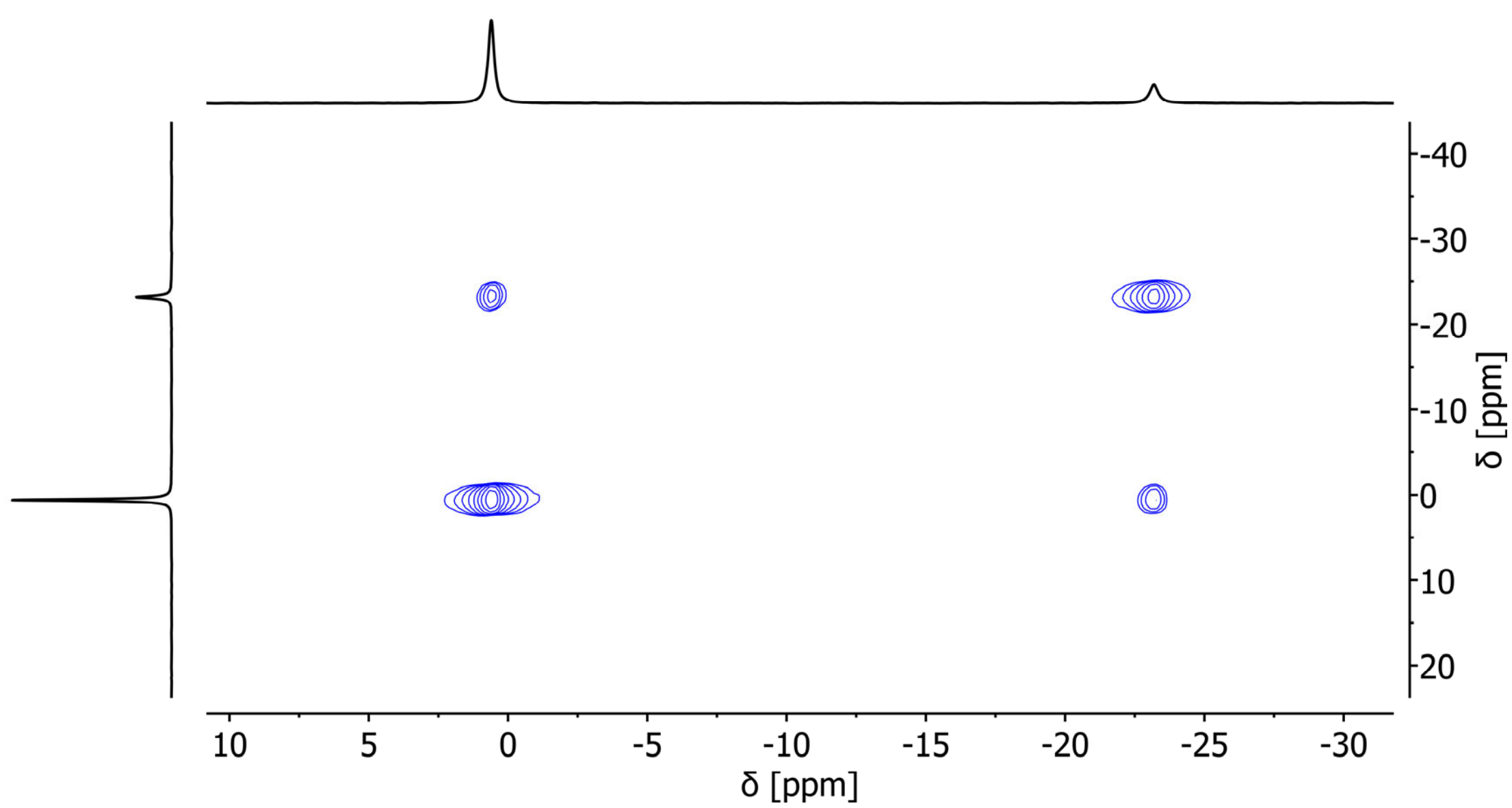

Figure S40: ^{31}P-^{31}P-EXSY-NMR spectrum of **UO-(NP^{pyrr})$_4$** in THF-d_8 at -95 °C with mixing time of 1 ms.

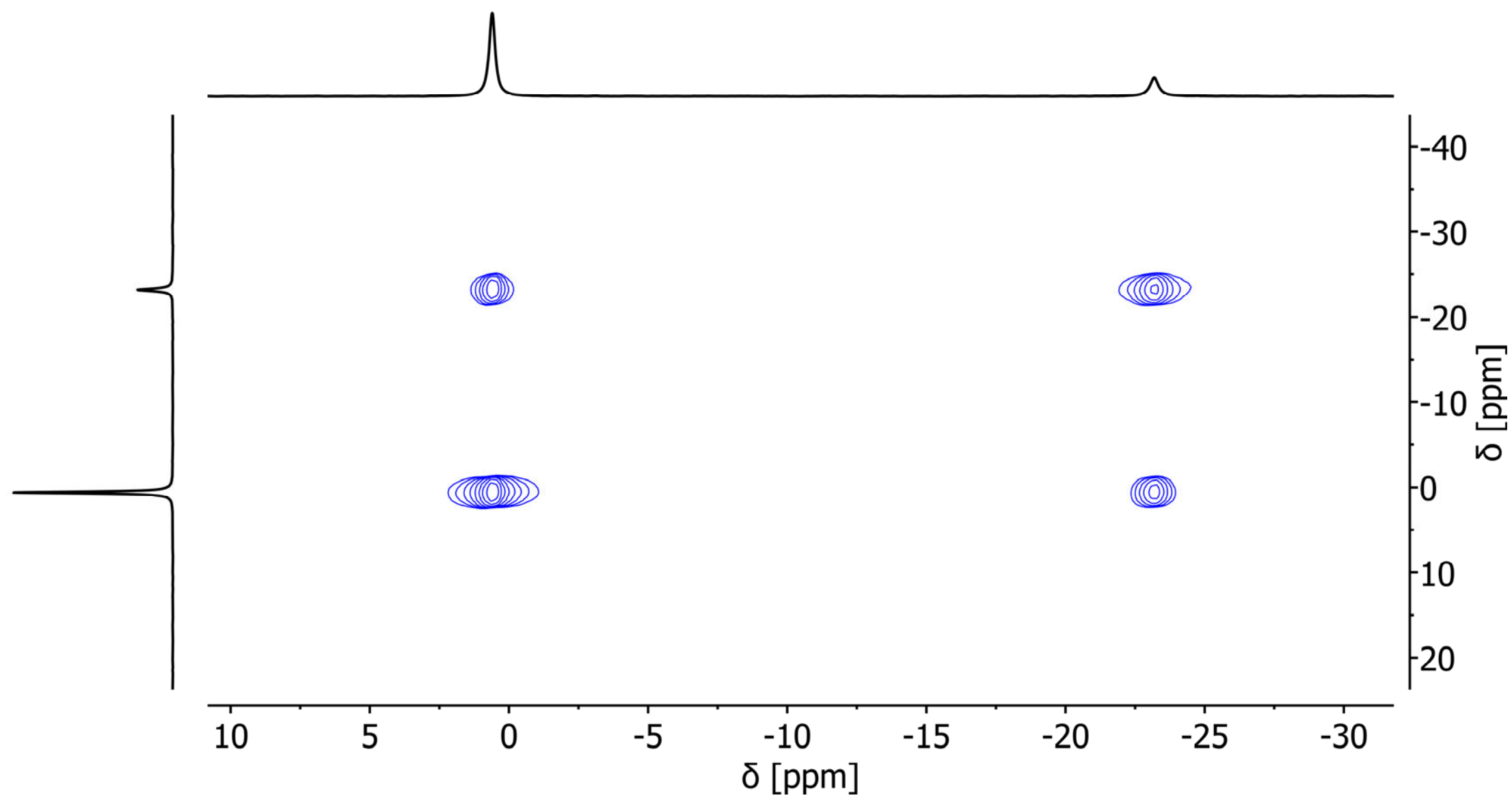

Figure S41: ^{31}P-^{31}P-EXSY-NMR spectrum of **UO-(NP^{pyrr})$_4$** in THF-d_8 at -95 °C with mixing time of 3 ms.

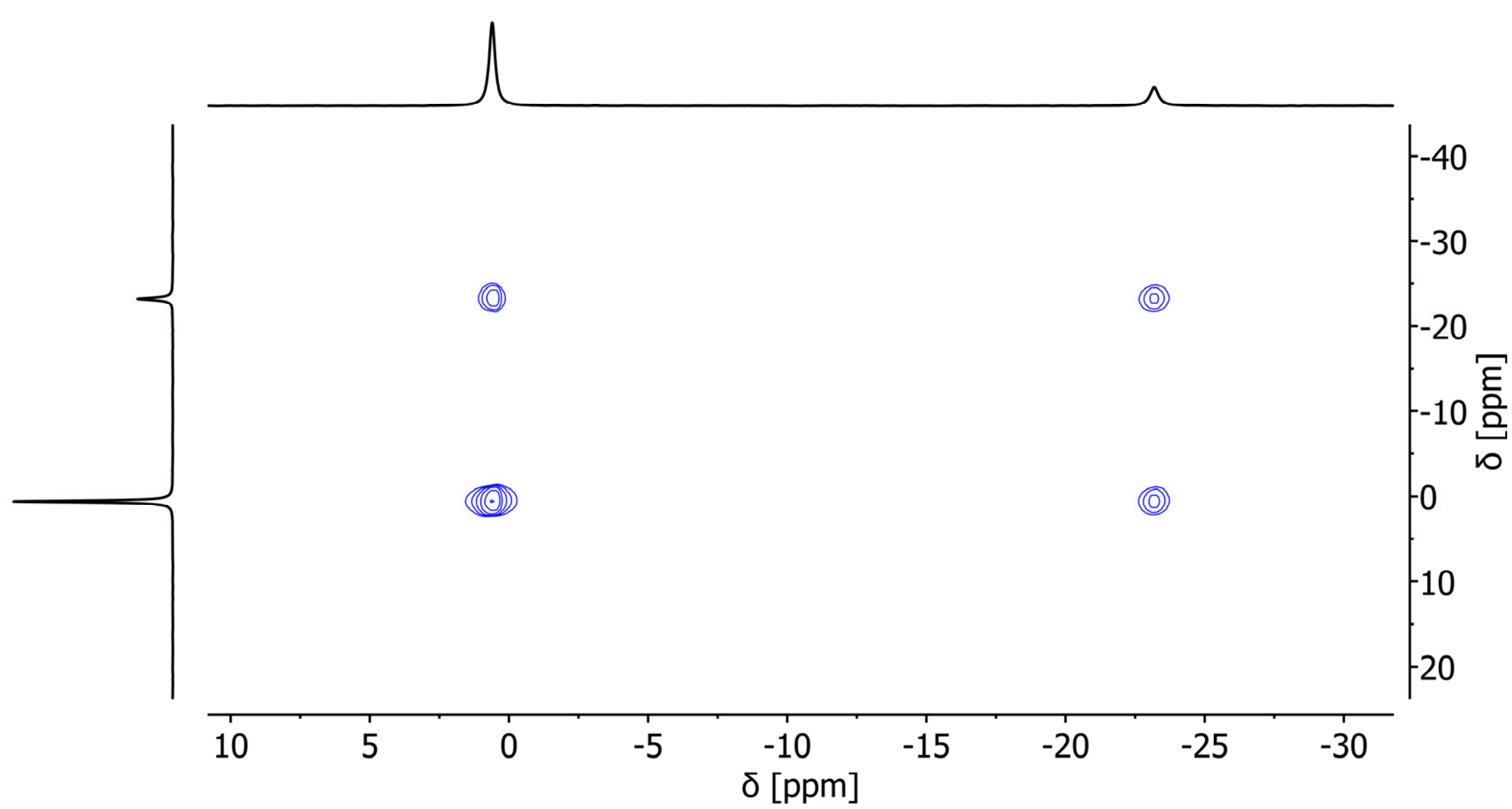

Figure S42: ^{31}P-^{31}P-EXSY-NMR spectrum of **UO-(NP^{pyrr})$_4$** in THF-d_8 at -95 °C with mixing time of 5 ms.

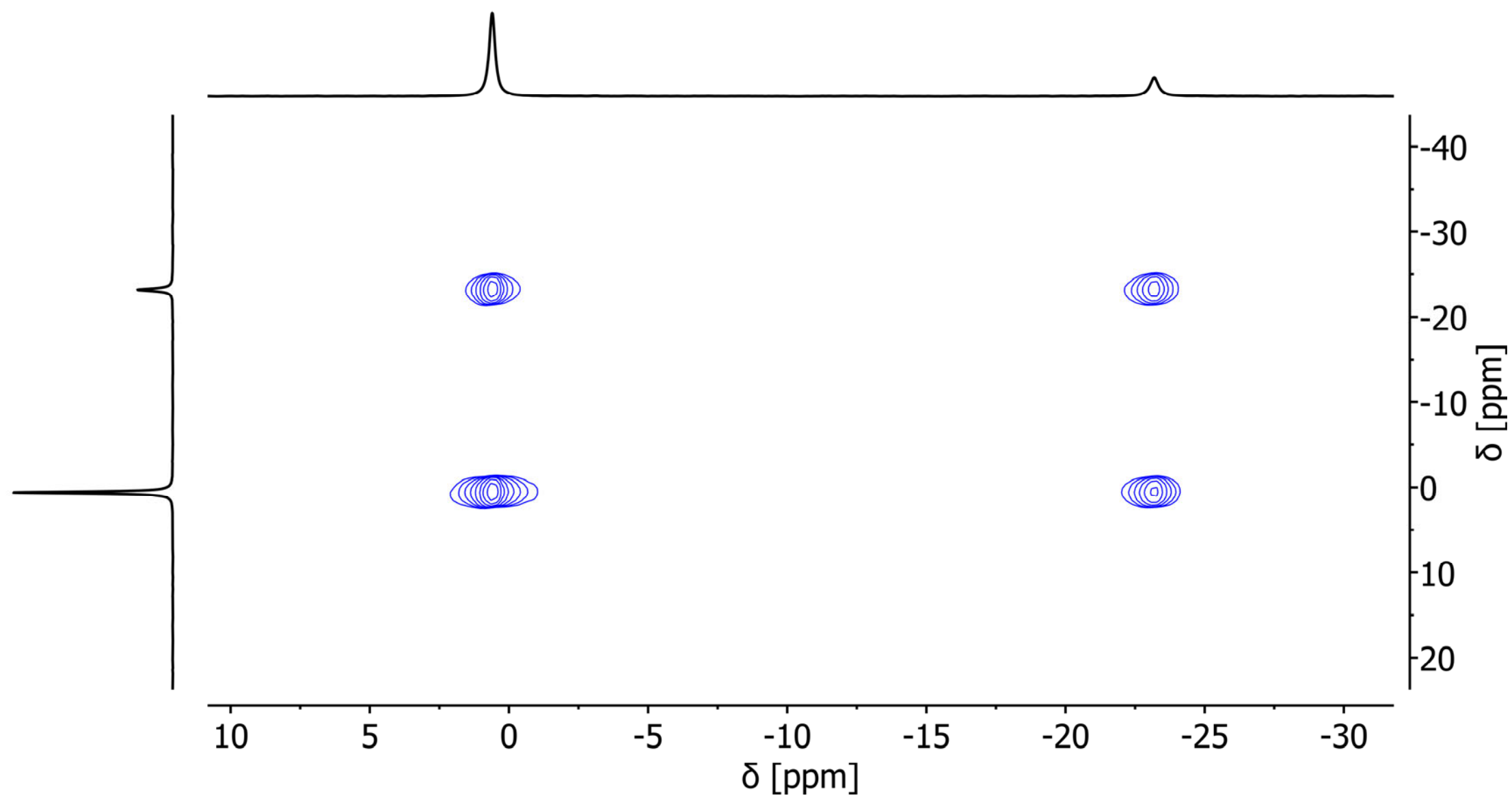

Figure S43: ^{31}P-^{31}P-EXSY-NMR spectrum of **UO-(NP^{pyrr})$_4$** in THF-d_8 at -95 °C with mixing time of 7 ms.

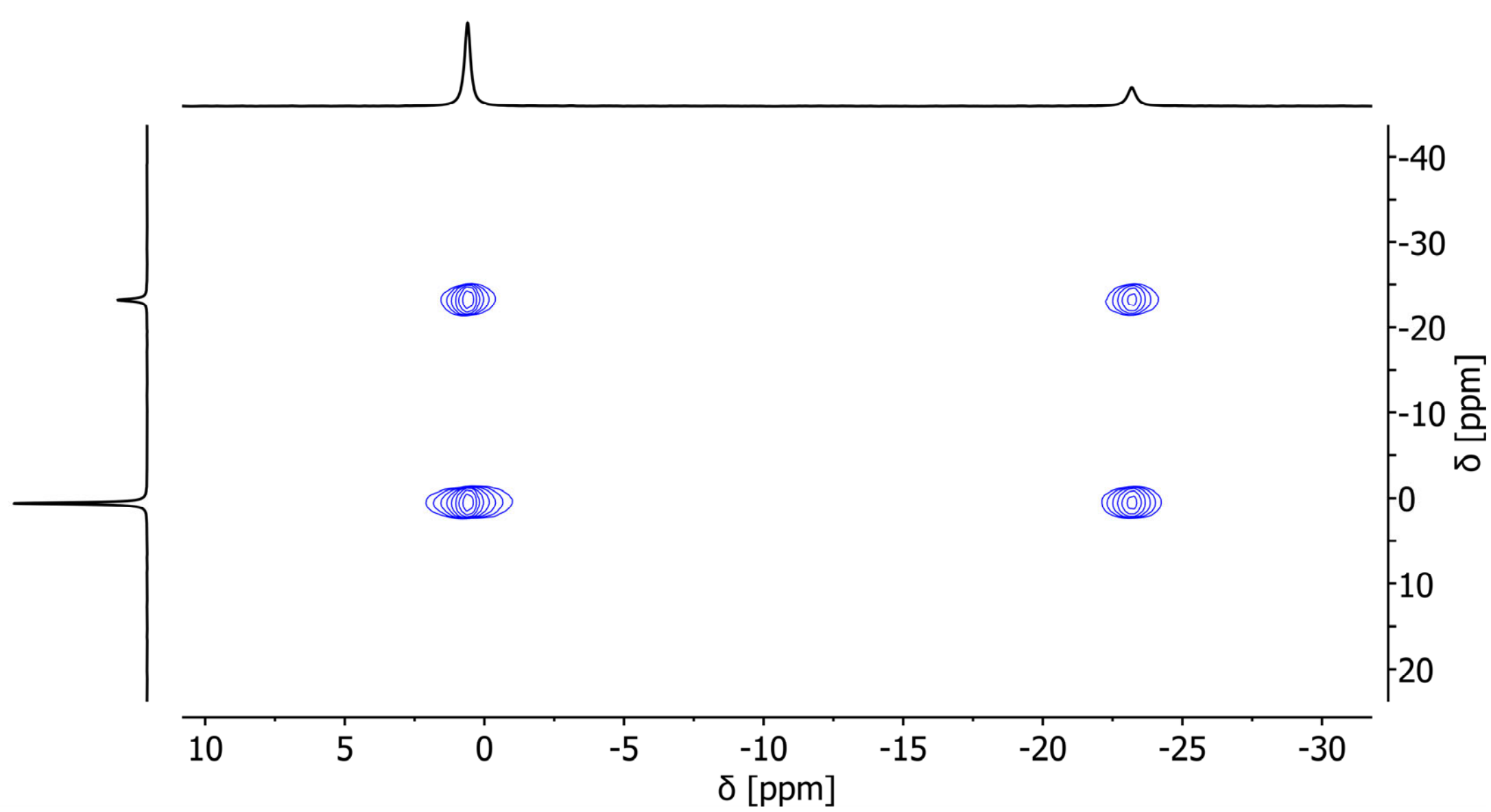

Figure S44: ^{31}P-^{31}P-EXSY-NMR spectrum of **UO-(NP^{pyrr})$_4$** in THF-d_8 at -95 °C with mixing time of 10 ms.

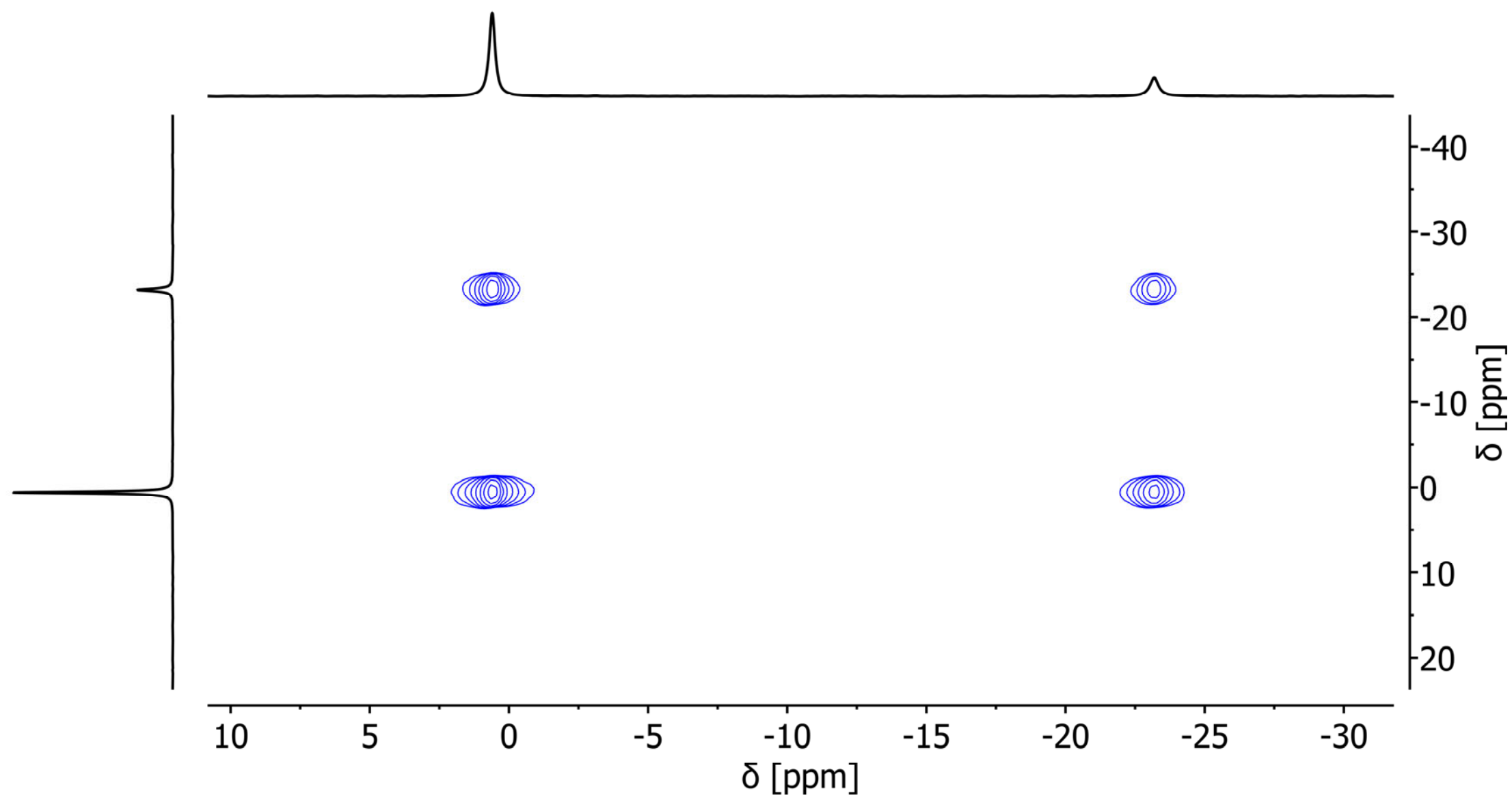

Figure S45: ^{31}P-^{31}P-EXSY-NMR spectrum of **UO-(NP^{pyrr})$_4$** in THF-d_8 at -95 °C with mixing time of 30 ms.

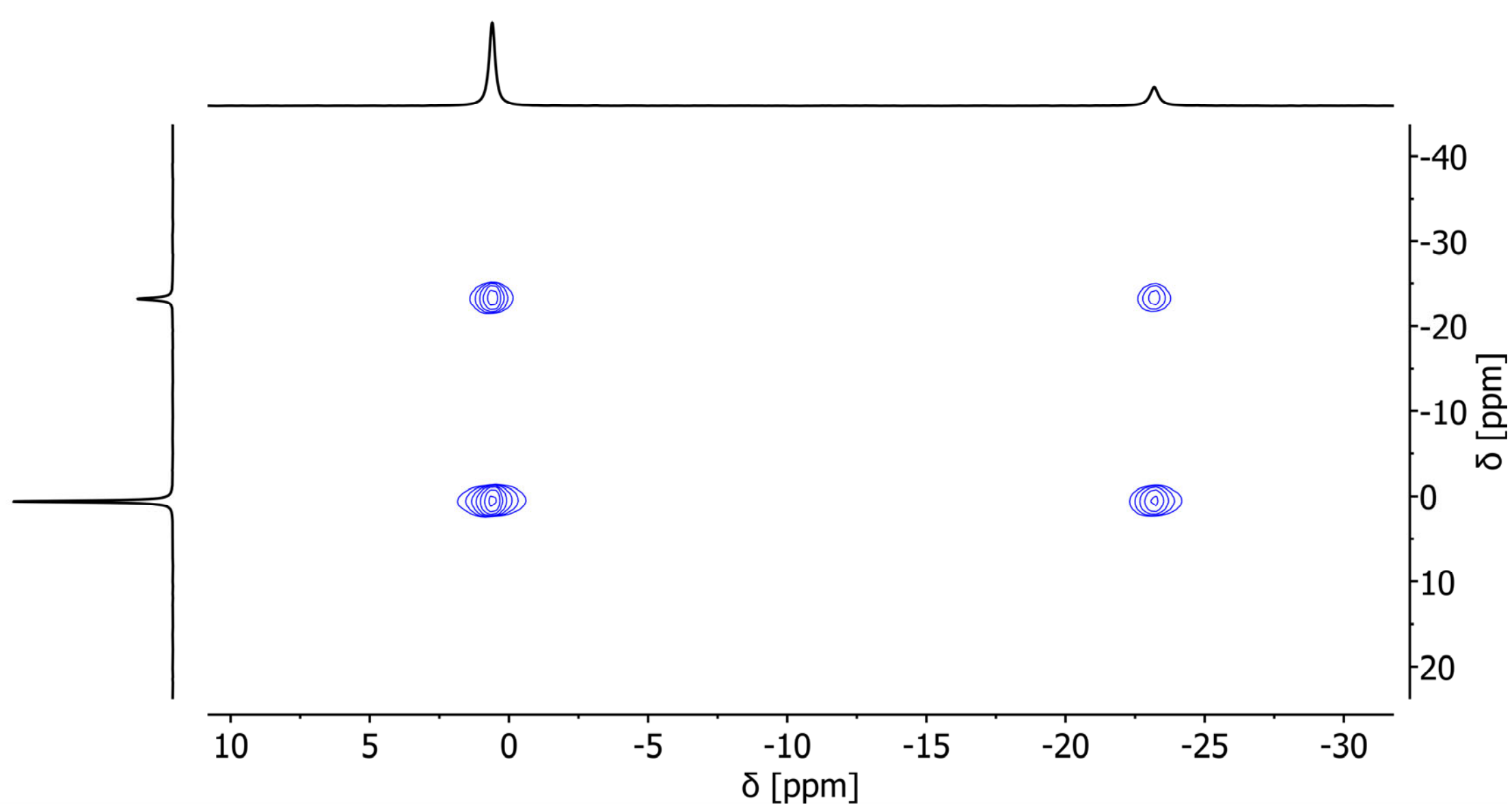

Figure S46: ^{31}P-^{31}P-EXSY-NMR spectrum of **UO-(NP^{pyrr})$_4$** in THF-d_8 at -95 °C with mixing time of 50 ms.

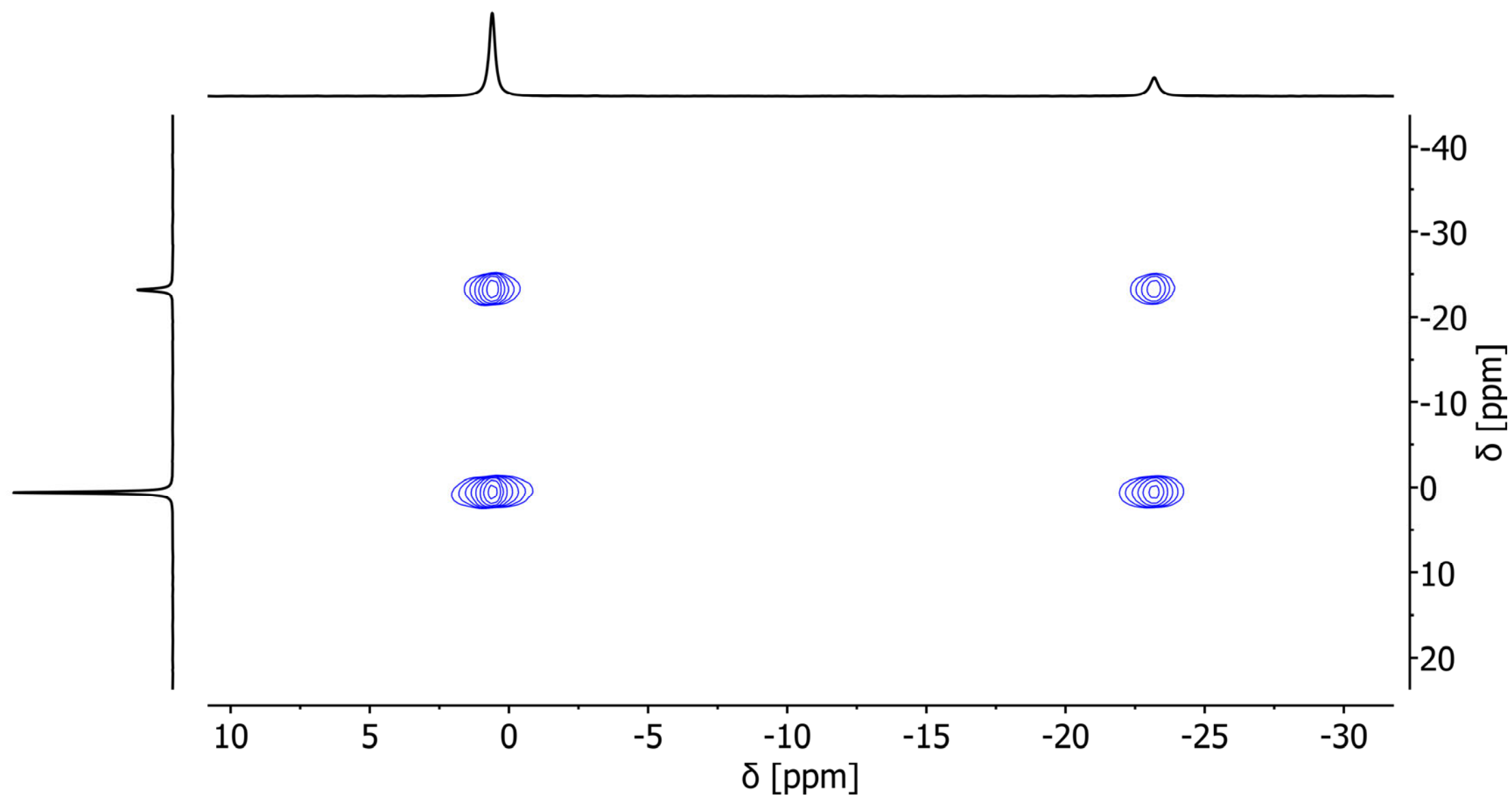

Figure S47: ^{31}P-^{31}P-EXSY-NMR spectrum of **UO-(NP^{pyrr})$_4$** in THF-d_8 at -95 °C with mixing time of 100 ms.

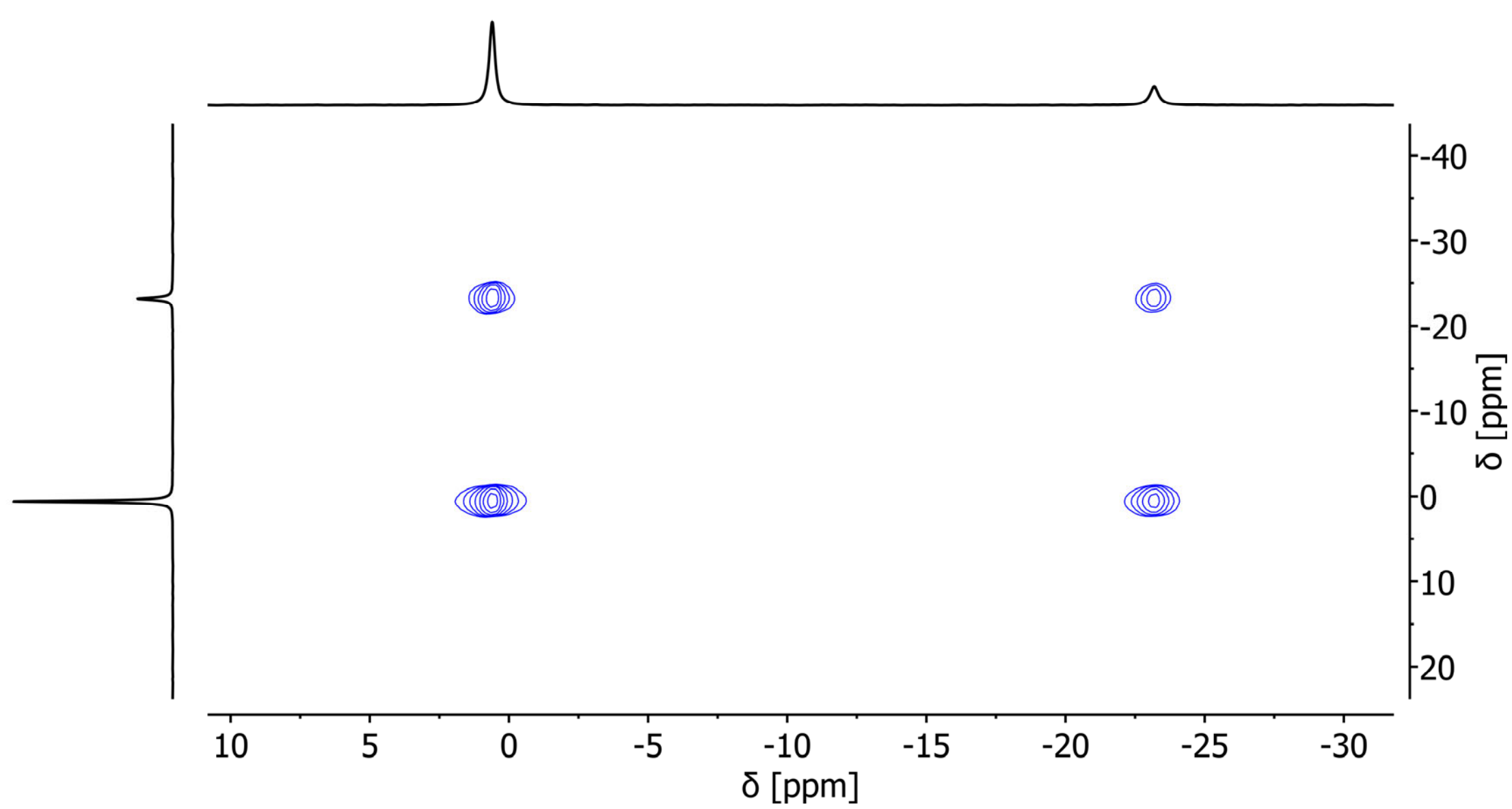

Figure S48: ^{31}P-^{31}P-EXSY-NMR spectrum of **UO-(NP^{pyrr})$_4$** in THF-d_8 at -95 °C with mixing time of 200 ms.

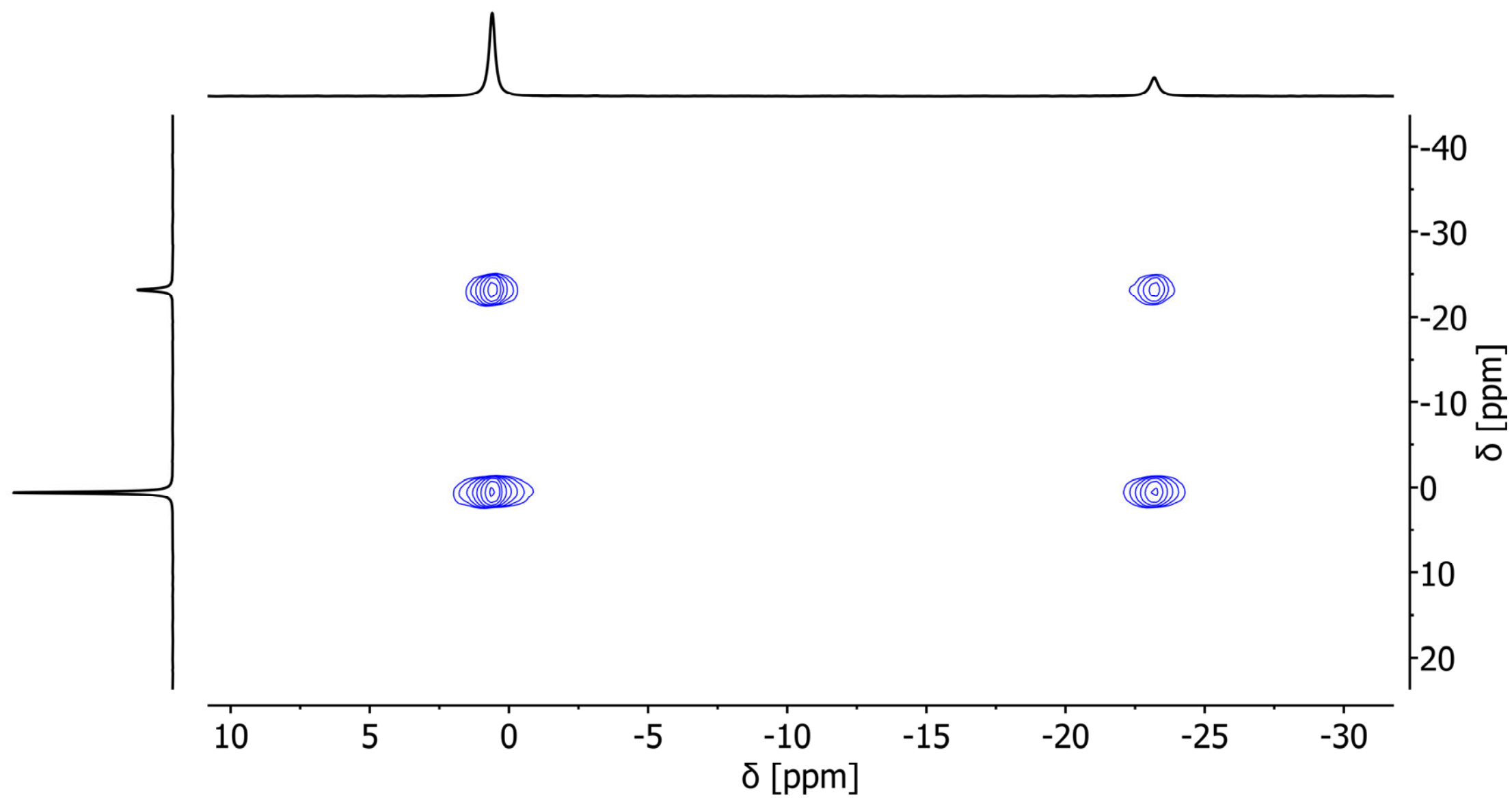

Figure S49: ${}^{31}P$-${}^{31}P$-EXSY-NMR spectrum of **UO-(NP^{pyrr})$_4$** in THF-d_8 at -95 °C with mixing time of 500 ms.

4 UV-vis-NIR Spectroscopy

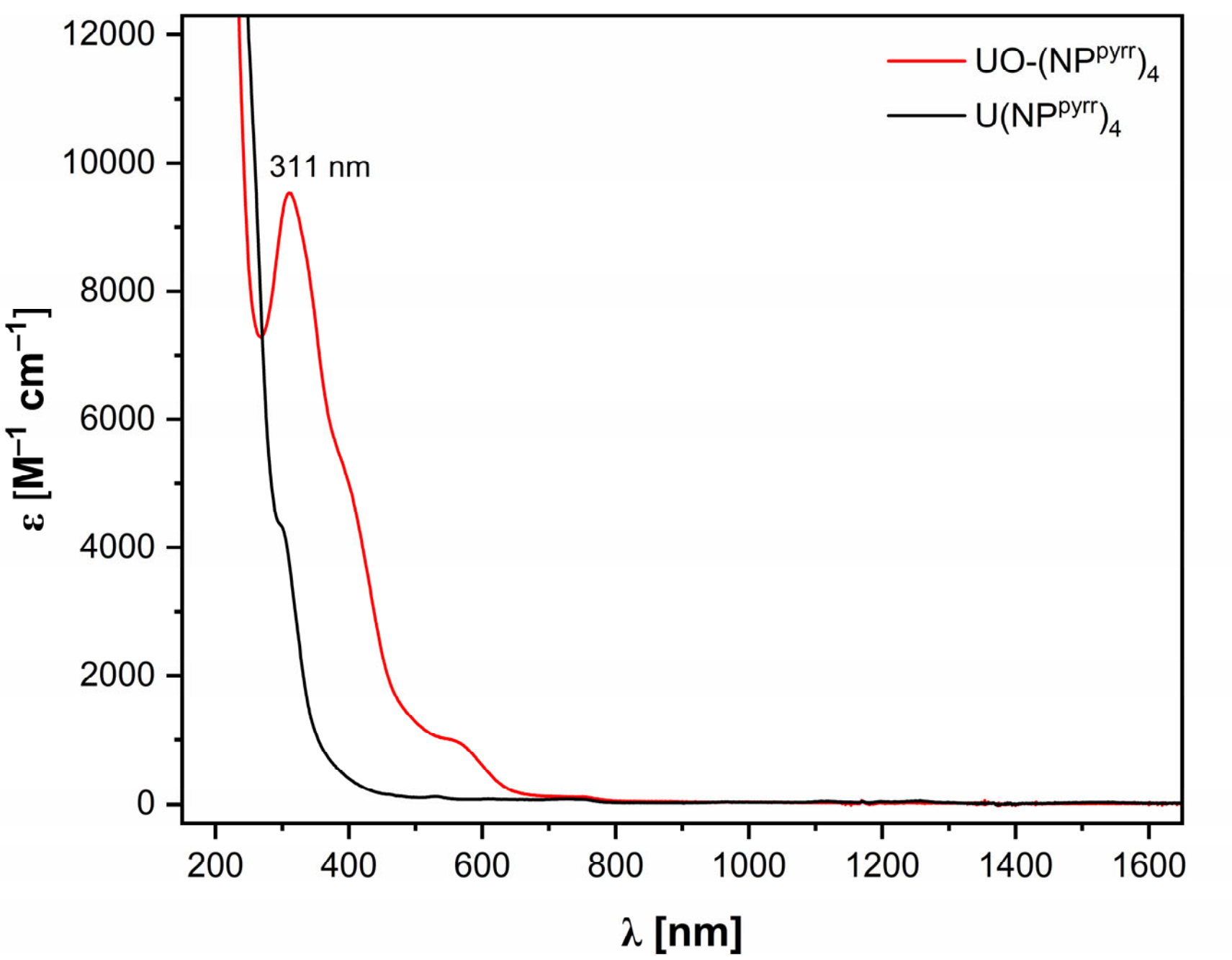

Figure S50: UV-vis-NIR absorption spectra of **U(NP^{pyrr})$_4$** (black; 301 µM, THF) and **UO-(NP^{pyrr})$_4$** (red; 203 µM, THF).

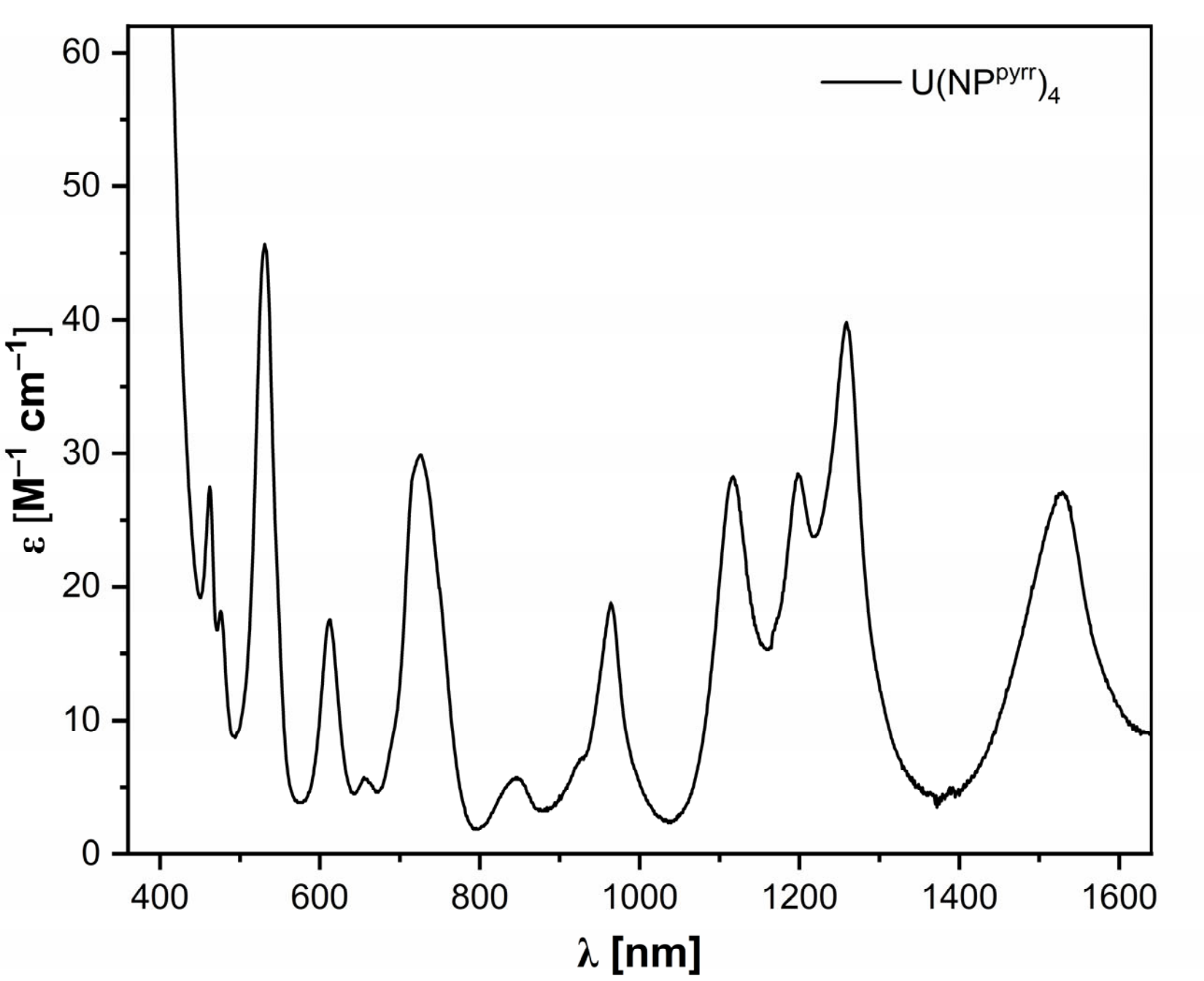

Figure S51: Expansion of UV-vis-NIR spectrum of **U(NP^{pyrr})$_4$** (8.04 mM, THF) between 400 nm and 1600 nm to highlight *f*-*f* transitions.

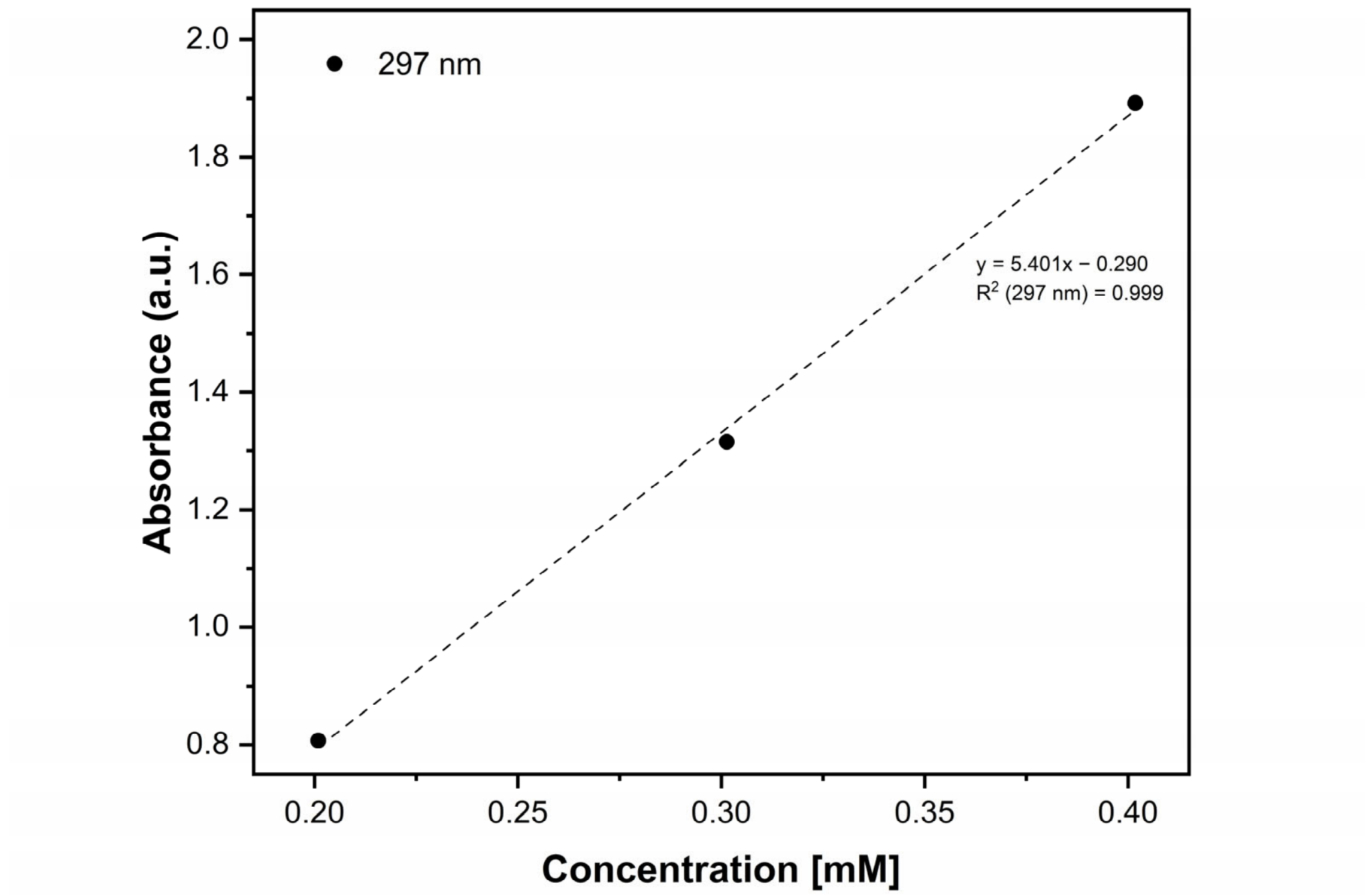

Figure S52: Linear regression of absorbance for shoulder at 297 nm in the UV-vis-NIR spectra of **U(NP^{pyrr})$_4$** over a range of concentrations.

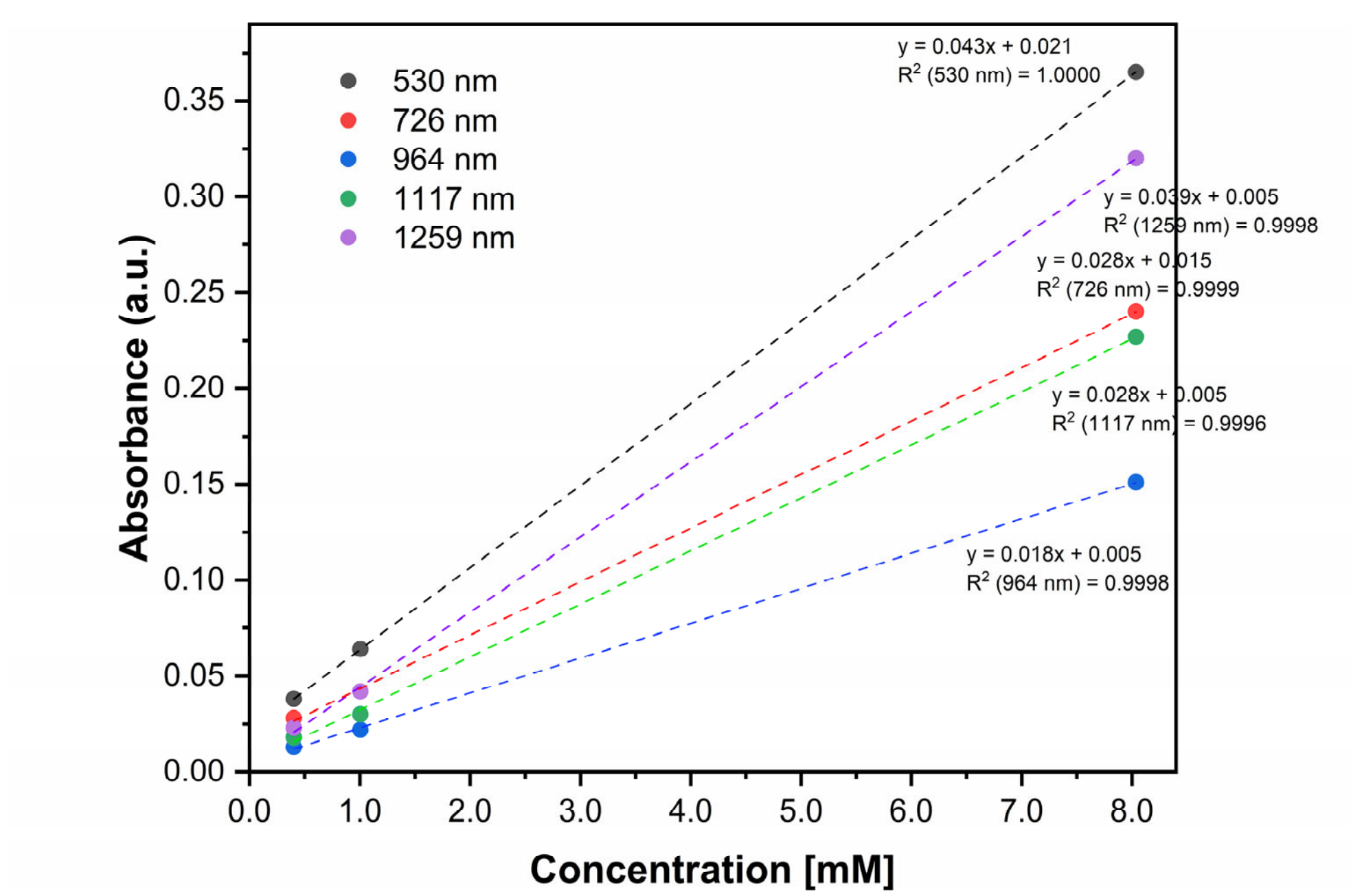

Figure S53: Linear regression of absorbance for selected peaks in the UV-vis-NIR spectra of **U(NP^{pyrr})$_4$** over a range of concentrations.

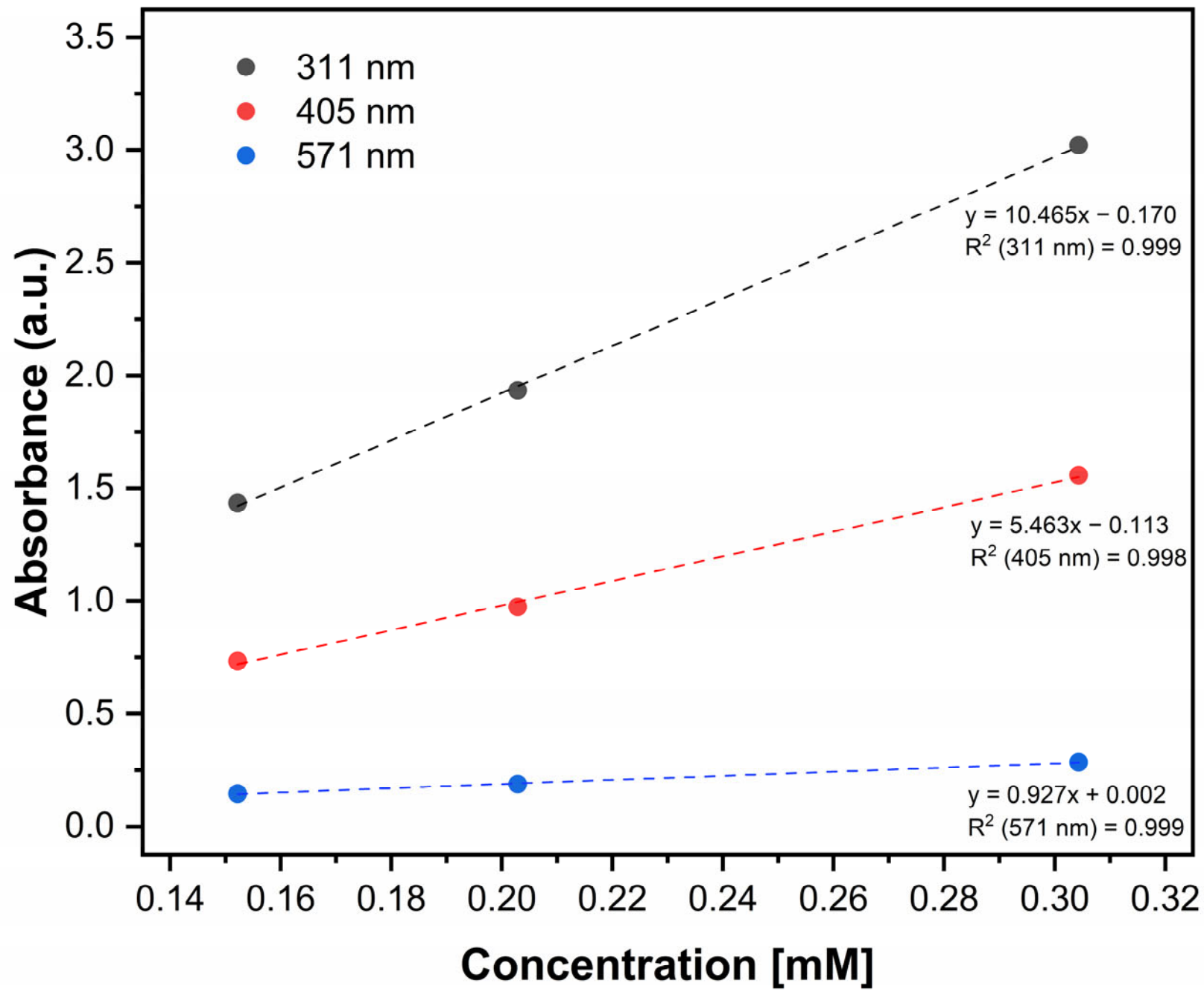

Figure S54: Linear regression of absorbance for selected peaks in the UV-vis-NIR spectra of **UO-(NP^{pyrr})$_4$** over a range of concentrations.

5 IR Spectroscopy

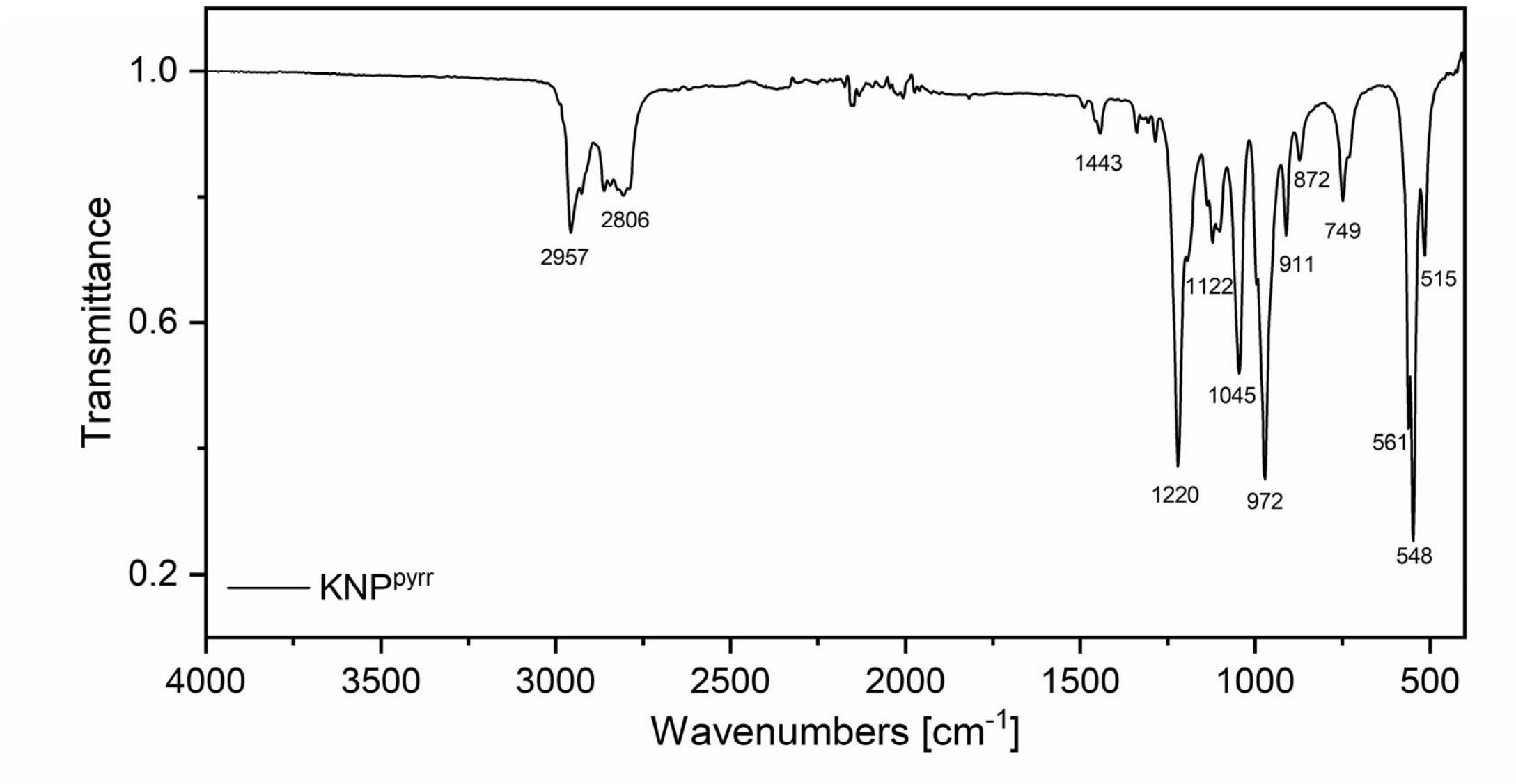

Figure S55: FT-IR spectrum of **KNP^{pyrr}**.

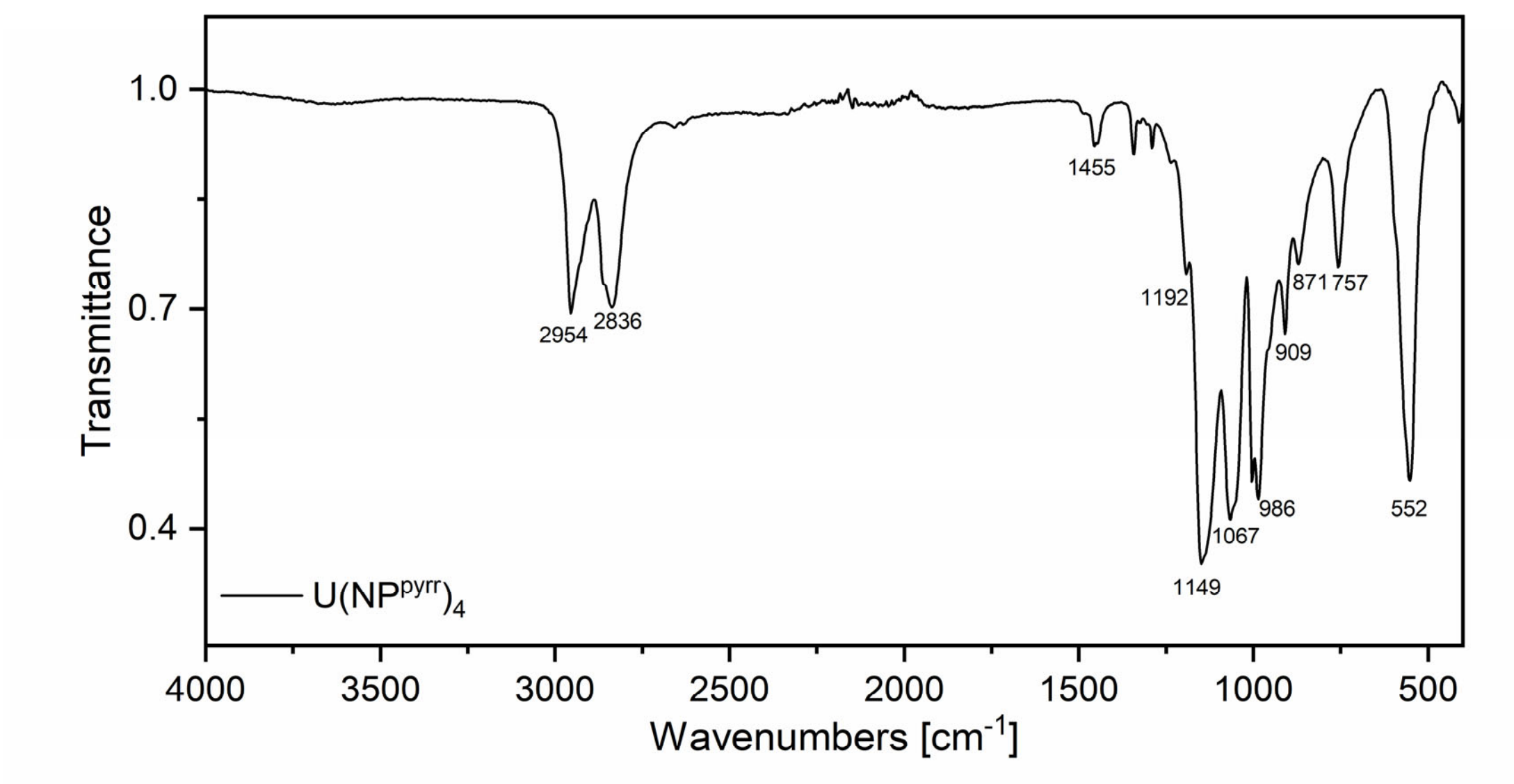

Figure S56: FT-IR spectrum of **U(NP^{pyrr})$_4$.**

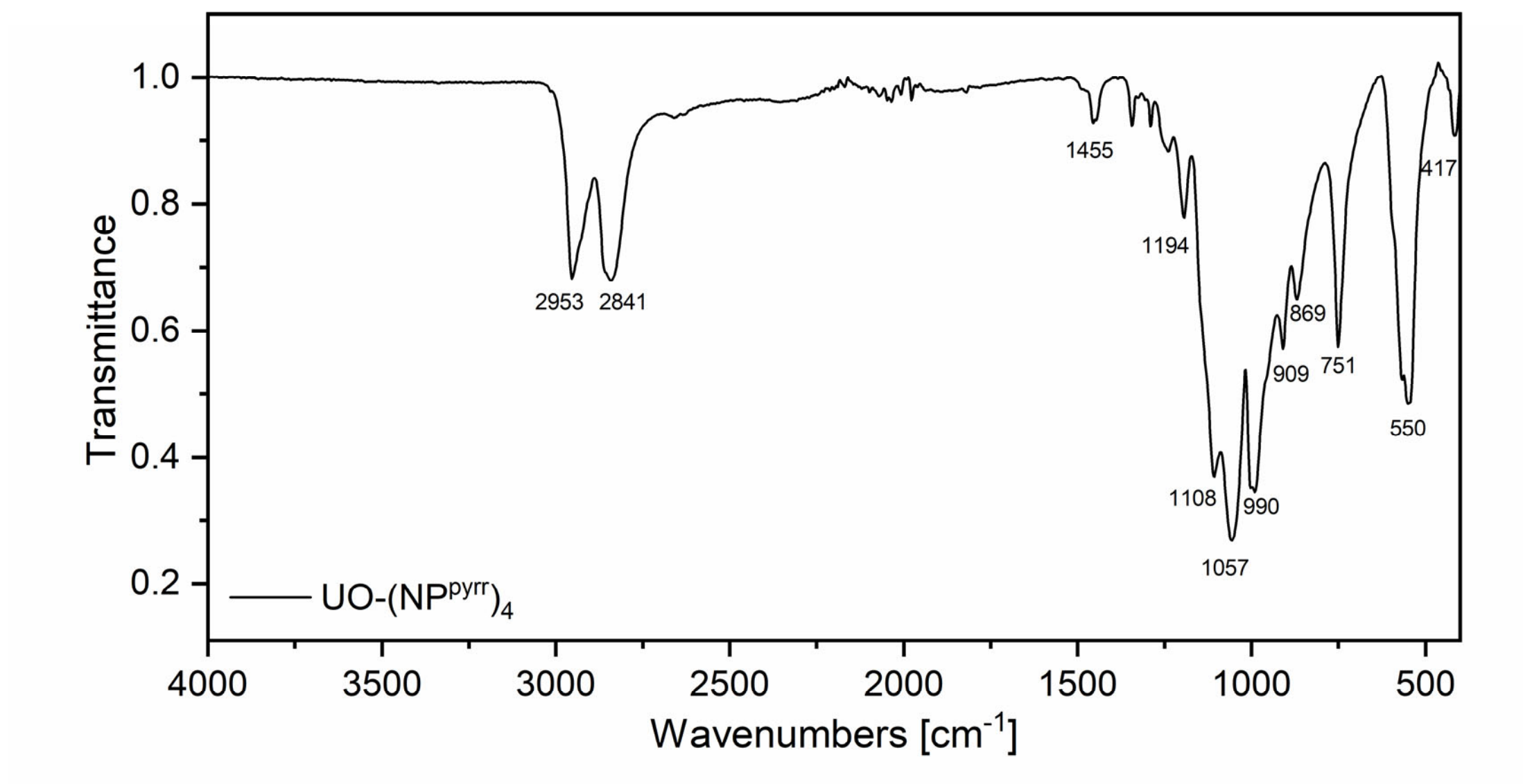

Figure S57: FT-IR spectrum of **UO-(NP^{pyrr})$_4$**.

6 SC-XRD

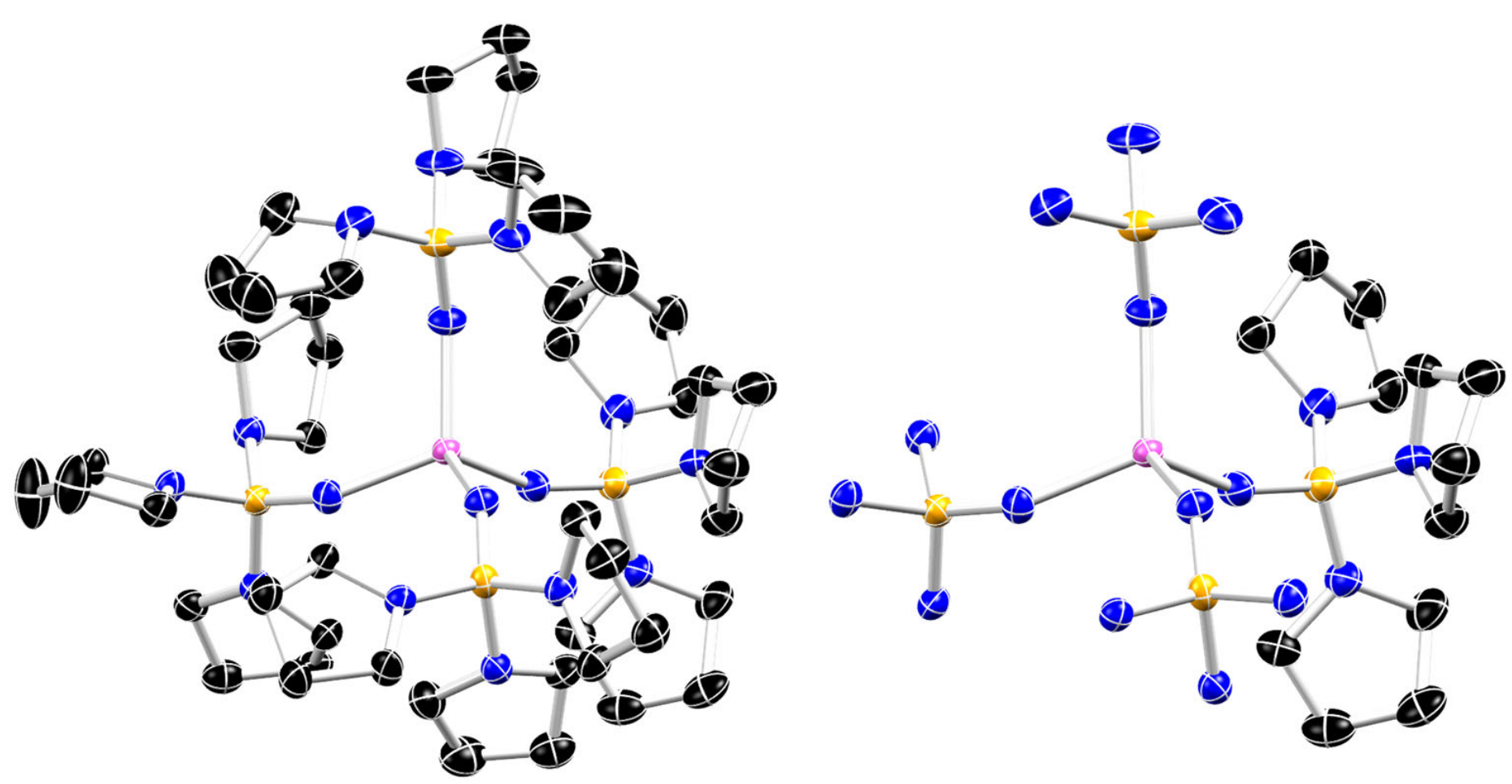

Figure S58: Molecular structure of **U(NP^{pyrr})$_4$**. Thermal ellipsoids at 50 % probability (C: black, N: blue, P: orange, U: pink). Hydrogen atoms and disorder are omitted for clarity. Only one of four molecules in asymmetric unit is shown. Left: Full molecule; Right: Truncated structure with only one ligand shown, the others are truncated to the pyrrolidine N-atom.

Table S4: Crystallographic data for **U(NP^{pyrr})$_4$.**

	**U(NP^{pyrr})$_4$**
**Empirical formula**	$C_{48}H_{96}N_{16}P_4U$
**Formula weight**	1259.31
**Temperature [K]**	100.00
**Crystal System**	triclinic
**Space Group**	$P\overline{1}$
**Resolution [Å]**	0.83
**I/σ**	10.7
***a* [Å]**	14.1222(9)
***b* [Å]**	21.2434(16)
***c* [Å]**	37.762(3)
**α [°]**	91.456(3)
**β [°]**	91.984(3)
**γ [°]**	92.933(3)
**Volume [Å^3]**	11302.9(13)
**Z**	8
**Z′**	4
**ρ_{calc} [g/cm^{-3}]**	1.480
**μ [mm^{-1}]**	3.034
***F*(000)**	5184
**Crystal size [mm^3]**	0.1×0.155×0.155
**Physical Description**	clear light pink blocks
**Radiation**	MoK_{α} (λ=0.71073 Å)
**2θ range for data collection [°]**	3.67 to 50.70
**Index ranges**	−17 ≤ h ≤ 17, −25 ≤ k ≤ 25, −45 ≤ l ≤ 45
**Reflections collected**	434547
**Independent reflections**	41355 (R_{int} = 0.1703 R_{sigma} = 0.0938)
**Data/Restraints/Parameters**	41355 / 2633 / 2841
**Goodness-of-fit on F^2**	1.038
**Final *R* indexes [I≥2σ(I)]**	R_1 = 0.0580 wR_2 = 0.0948
**Final *R* indexes [all data]**	R_1 = 0.1113 wR_2 = 0.1137
**Largest peak/hole [e Å^{-3}]**	1.94/−1.65
**Completeness to 2θ = 50.484°**	99.9 %
**CCDC number**	2546966

Initial indexing suggested two possible unit cells at 5653 Å^3 and 11303 Å^3. Harvesting and indexing the highest intensity reflections gave a unit cell with size 12.4507(8) Å x 13.0535(8) Å x 37.768(2) Å in $P\overline{1}$. When integrating all available reflections, this cell only accounts for 60 % of the selected reflections and 30 % of the overall reflections. After integration and scaling, the overall structure solution consists of two molecules in the asymmetric unit displaying whole-molecule disorder at 50 % occupancy.

The degree of disorder and omission of reflections prompted reindexing and reintegration. A unit cell with dimensions 14.1222(9) Å x 21.2434(16) Å x 37.762(3) Å in $P\overline{1}$ was found, which is related to the original cell by the transformation matrix [-1 1 0; -1 -1 0; 0 0 2]. This cell accounts for 86 % of the overall reflections used in indexing. After integration and scaling, the overall structure solution consists of four molecules in the asymmetric unit. Individual pyrrolidine groups display positional disorder with a range of refined fractional occupancies, but no modeling of disorder at metal centers was required and the overall degree of disorder has decreased. This solution is more representative of the collected data due to the decreased overall disorder and more complete account of weaker reflections.

UO-(NP^{pyrr})$_4$ crystallized in the space group $R\overline{3}$. Heavy disorder including whole molecule disorder on a crystallographic special position prevented satisfactory refinement of this structure. An xyz file was generated from the SC-XRD data and used as starting model for computational studies. The molecular structure based on that xyz file is represented as ball and stick model below.

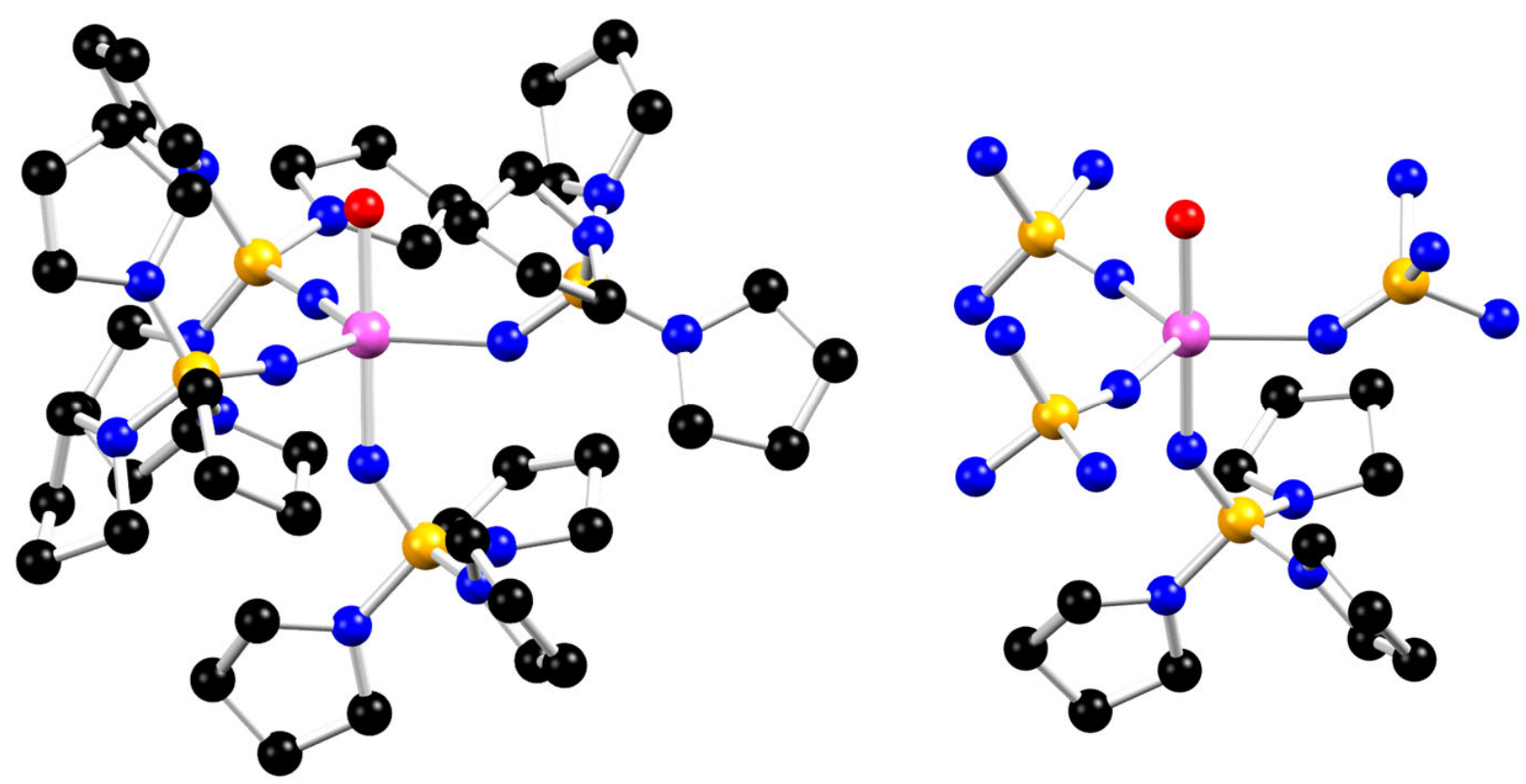

Figure S59: Ball and stick model of molecular structure of **UO-(NP^{pyrr})$_4$** with C: black, N: blue, P: orange, U: pink. Left: Full molecule; Right: Truncated structure with only one ligand shown, the others are truncated to the pyrrolidine N-atom. Hydrogen atoms are omitted for clarity.

7 Computational Supporting Data

7.1 DFT Geometry Optimizations

DFT geometries are optimized for four complexes given in **Figure S60**. Selected geometric parameters are included in **Table S5** and **S6**. Regardless of functional choice, geometries are in reasonable agreement with experiment, with the best agreement for systems where the solid-state characterization has the highest resolution. Since TPSS is the best performing of the two metaGGA functionals and performs similarly to PBE, further analysis is performed on those structures.

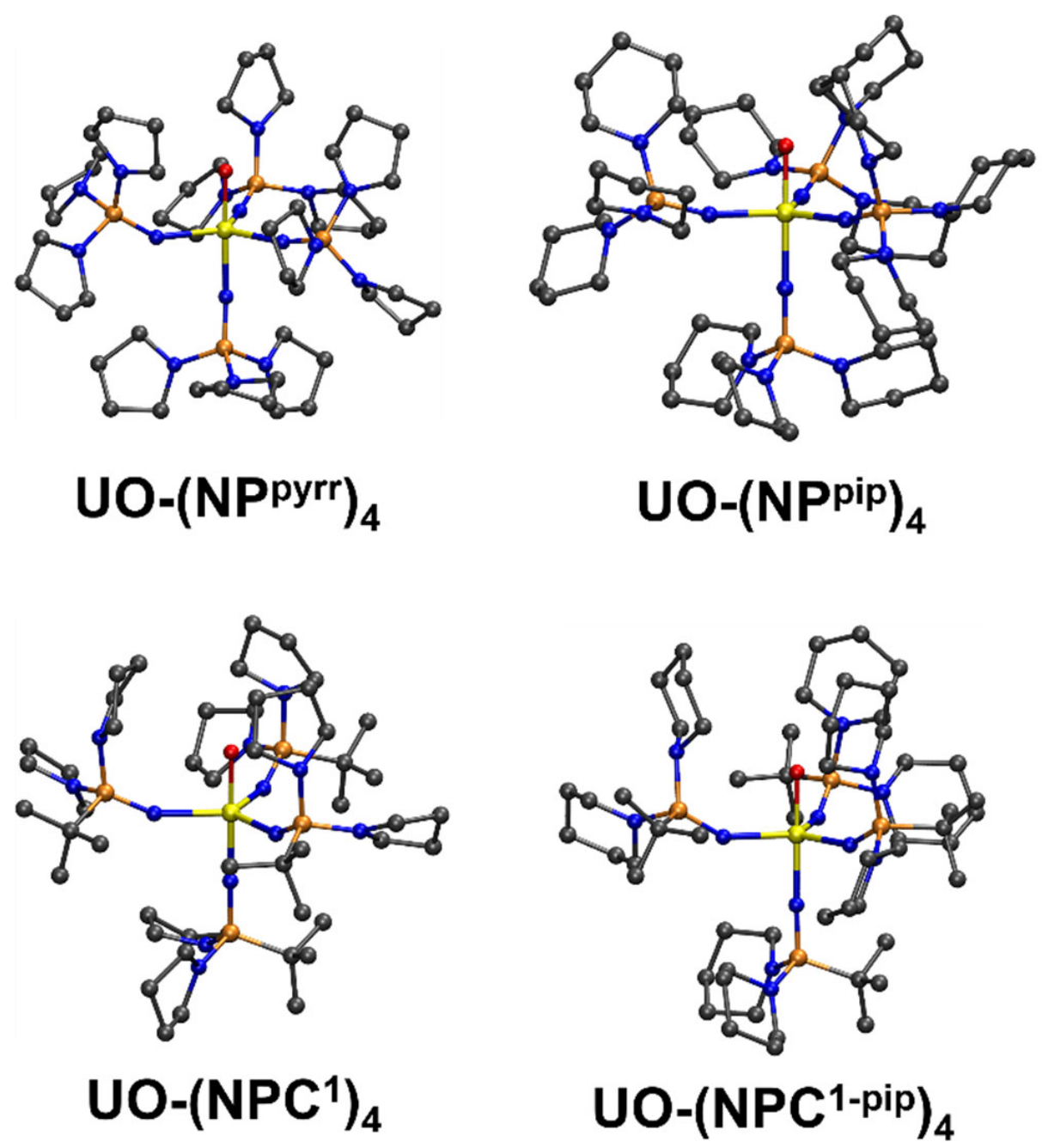

Figure S60: DFT optimized structures of the four complexes proposed to contain an ITI. U in yellow, N in blue, O in red, P in orange, C in grey. H atoms are hidden for clarity.

Table S5: Selected DFT geometric parameters for comparison with experiment. The PBE, TPSS, and MN15-L functionals are employed. Bond distances in Å. The ITI% is the ratio of the U-N_{ax} and U-N_{eq} (avg) bonds.

Ligand	Structure	U=O	U-N_{ax}		U-N_{eq}		U-N_{eq} (avg)	ITI%
**UO-(NP^{pyrr})$_4$**	Exp	1.964	2.033	2.164	2.100	2.120	2.128	95.5
	PBE	1.856	2.091	2.179	2.212	2.190	2.194	95.3
	TPSS	1.851	2.096	2.191	2.207	2.191	2.196	95.4
	MN15-L	1.819	2.076	2.169	2.193	2.158	2.173	95.5
**UO-(NP^{pip})$_4$**	Exp	1.89	1.948	2.160	2.160	2.160	2.160	90.2
	PBE	1.851	2.085	2.195	2.195	2.195	2.195	95.0
	TPSS	1.848	2.094	2.199	2.199	2.199	2.199	95.2
	MN15-L	1.808	2.051	2.178	2.176	2.177	2.177	94.2
**UO-(NPC^1)$_4$**	Exp	1.827	2.034	2.170	2.154	2.179	2.168	93.8
	PBE	1.854	2.088	2.205	2.196	2.196	2.199	95.0
	TPSS	1.849	2.094	2.211	2.202	2.203	2.205	95.0
	MN15-L	1.823	2.058	2.190	2.176	2.172	2.179	94.4
**UO-($NPC^{1\text{-}pip}$)$_4$**	Exp	1.819	2.002	2.175	2.138	2.157	2.157	92.8
	PBE	1.850	2.090	2.207	2.195	2.205	2.202	94.9
	TPSS	1.846	2.092	2.207	2.207	2.195	2.203	95.0
	MN15-L	1.808	2.027	2.191	2.195	2.211	2.199	92.2

Table S6: Selected DFT angles in degrees for comparison with experiment. The PBE, TPSS, and MN15-L functionals are employed. The three U-N-P angles for the equatorial ligands are averaged and the standard deviation is reported.

Complex	Structure	U-N_{ax}-P	U-N_{eq}-P	St. Dev.
**UO-$(NP^{pyrr})_4$**	Exp	146.9	156.8	13.1
	PBE	158.6	155.9	12.7
	TPSS	171.0	158.5	10.6
	MN15-L	139.2	149.0	6.0
**UO-$(NP^{pip})_4$**	Exp	180.0	144.9	0.0
	PBE	179.8	163.3	0.1
	TPSS	179.9	164.2	0.0
	MN15-L	179.8	154.0	13.5
**UO-$(NPC^{1})_4$**	Exp	177.0	151.2	0.8
	PBE	178.8	152.7	1.5
	TPSS	178.6	153.5	1.6
	MN15-L	154.8	145.1	6.5
**UO-$(NPC^{1\text{-}pip})_4$**	Exp	178.0	147.3	0.6
	PBE	178.0	148.0	1.5
	TPSS	177.2	148.2	1.6
	MN15-L	177.0	141.2	1.8

7.2 Bond Order Results

Table S7: Selected bond distances (in Å) and bond orders for the four complexes. Results were computed on the TPSS geometry from Turbomole at the TPSS/TZP level of theory. Both Mayer and Nalewajski-Mrozek Type 3 (NM-3) bond orders are reported.

	Bond	Distance	Mayer	NM-3
**UO-(NP^{pyrr})$_4$**	U-O	1.851	1.922	2.620
	U-N$_{ITI}$	2.096	1.132	1.783
	U-N	2.191	0.944	1.547
	U-N	2.191	0.945	1.544
	U-N	2.207	0.978	1.525
**UO-(NP^{pip})$_4$**	U-O	1.848	1.910	2.585
	U-N$_{ITI}$	2.094	1.174	1.792
	U-N	2.199	0.959	1.535
	U-N	2.199	0.959	1.534
	U-N	2.199	0.957	1.533
**UO-(NPC^1)$_4$**	U-O	1.849	1.890	2.651
	U-N$_{ITI}$	2.094	1.176	1.794
	U-N	2.211	0.942	1.512
	U-N	2.203	0.950	1.544
	U-N	2.202	0.935	1.536
**UO-(NPC$^{1\text{-}pip}$)$_4$**	U-O	1.846	1.882	2.636
	U-N$_{ITI}$	2.092	1.176	1.792
	U-N	2.207	0.971	1.514
	U-N	2.207	0.978	1.514
	U-N	2.195	0.977	1.541

Table S8: CM5 charges for the coordinating N atoms, computed on the TPSS optimized structures.

Functional	Structure	N$_{axial}$	N$_{equat}$		
**PBE-D3/TZP**	**UO-(NP^{pyrr})$_4$**	-0.7715	-0.7336	-0.7304	-0.7171
	**UO-(NP^{pip})$_4$**	-0.7770	-0.7230	-0.7230	-0.7229
	**UO-(NPC^1)$_4$**	-0.7743	-0.7204	-0.7215	-0.7239
	**UO-(NPC$^{1\text{-}pip}$)$_4$**	-0.7712	-0.7148	-0.7141	-0.7220
**TPSS-D3/TZP**	**UO-(NP^{pyrr})$_4$**	-0.7790	-0.7403	-0.7402	-0.7331
	**UO-(NP^{pip})$_4$**	-0.7829	-0.7333	-0.7333	-0.7333
	**UO-(NPC^1)$_4$**	-0.7822	-0.7298	-0.7303	-0.7331
	**UO-(NPC$^{1\text{-}pip}$)$_4$**	-0.7770	-0.7238	-0.7248	-0.7313

7.3 Computed ^{31}P-NMR Parameters

Table S9: The ^{31}P isotropic shielding constants for the H_3PO_4 acid reference, PPh_3 reference, the PH_3 reference, the free ligand references, and for the four complexes as computed on the TPSS geometry from Turbomole at the PBE/TZP level of theory. The shift associated with the axial position in bold.

	Scalar	Spin Orbit	Δ_{SO}
**H_3PO_4 Reference**	301.546	316.092	-14.546
**PPh_3 Reference**	304.025	315.986	-11.961
**PH_3 Reference**	584.328	597.194	-12.866
**HNP^{pyrr} Reference**	290.615	303.286	-12.671
**HNP^{pip} Reference**	284.158	309.443	-25.285
**$HNPC^{1}$ Reference**	270.852	283.365	-12.513
**$HNPC^{1\text{-}pip}$ Reference**	265.136	277.565	-12.429
**$UO\text{-}(NP^{pyrr})_4$**	328.970	315.514	13.456
	348.418	330.265	18.153
	**377.486**	**356.508**	**20.978**
	350.939	330.113	20.826
**$UO\text{-}(NP^{pip})_4$**	340.49	321.758	18.732
	**373.876**	**348.356**	**25.520**
	340.425	321.644	18.781
	340.221	321.524	18.697
**$UO\text{-}(NPC^{1})_4$**	**351.498**	**335.729**	**15.769**
	316.471	302.819	13.652
	313.427	301.201	12.226
	315.001	302.453	12.548
**$UO\text{-}(NPC^{1\text{-}pip})_4$**	291.914	279.733	12.181
	**346.808**	**326.446**	**20.362**
	293.579	280.933	12.646
	295.758	282.447	13.311

Table S10: The ^{31}P isotropic shielding constants for the H_3PO_4 acid reference, PPh_3 reference, the PH_3 reference, the free ligand references, and for the four complexes as computed on the TPSS geometry from Turbomole at the PBE0/TZP level of theory. The shift associated with the axial position in bold.

	**Scalar**	**Spin Orbit**	**Δ_{SO}**
**H_3PO_4 Reference**	316.173	331.363	-15.19
**PPh_3 Reference**	329.275	341.471	-12.196
**PH_3 Reference**	592.884	605.873	-12.989
**HNP^{pyrr} Reference**	302.929	316.04	-13.111
**HNP^{pip} Reference**	296.347	309.443	-13.096
**$HNPC^{1}$ Reference**	284.732	297.68	-12.948
**$HNPC^{1\text{-}pip}$ Reference**	279.392	292.267	-12.875
**$UO\text{-}(NP^{pyrr})_4$**	337.871	329.624	8.247
	356.988	343.335	13.653
	**396.394**	**368.307**	**28.087**
	360.477	343.381	17.096
**$UO\text{-}(NP^{pip})_4$**	350.379	335.087	15.292
	**393.349**	**359.342**	**34.007**
	350.363	335.177	15.186
	350.189	335.087	15.102
**$UO\text{-}(NPC^{1})_4$**	**370.960**	**350.439**	**20.521**
	326.897	318.654	8.243
	324.237	317.645	6.592
	325.566	318.635	6.931
**$UO\text{-}(NPC^{1\text{-}pip})_4$**	303.408	297.207	6.201
	**365.794**	**339.285**	**26.509**
	305.453	298.785	6.668
	307.35	299.794	7.556

Focusing first on NMR spectroscopy, the chemical shifts (δ) and nuclear shielding constants (σ) were computed. The nuclear shielding constant (σ) is comprised of three components:

$$\sigma = \sigma_{dia} + \sigma_{para} + \sigma_{SO} \qquad (17)$$

where σ_{dia} is the diamagnetic contribution, σ_{para} is the paramagnetic contribution, and σ_{SO} is the spin–orbit contribution. The magnitude of σ_{SO} is used for assessing M-L covalency and correlates to the amount of ligand *s*-character and M-L orbital overlap present in the M-L bonds.[19, 20] The σ_{SO} value can be indirectly obtained from DFT as the difference in σ values from a scalar relativistic (SR) and spin–orbit (SO) calculation. This computed relativistic shift is referred to as Δ_{SO}.

On the other hand, the chemical shift including spin–orbit coupling (δ_{SO}) is the value directly comparable to experiment. The ^{31}P chemical shifts are reported with reference to an H_3PO_4 acid reference. The computed values use the following equation to compute the DFT chemical shift with respect to the H_3PO_4 acid reference (δ_{acid}) directly from the nuclear shielding constant for both the reference ($\sigma_{acid,ref}$) and the complex ($\sigma_{complex}$) as shown in the following equation:

$$\delta_{acid} = \sigma_{acid,ref} - \sigma_{complex} \qquad (18)$$

This equation can be used computing the nuclear shielding constants both with scalar relativistic methods or spin-orbit corrections. The results from DFT using the acid reference are reported for the PBE and PBE0 functionals in **Tables S11** and **S12**, respectively.

Table S11: Average ^{31}P chemical shifts in ppm for the H_3PO_4 acid reference and for the four complexes as computed on the TPSS geometry from Turbomole at the PBE/TZP level of theory. Both the scalar-relativistic chemical shift, δ_{SR}, and the spin-orbit chemical shift, δ_{SO}, are given. The difference between the two is the spin-orbit shift, Δ_{SO}. Experimental values should be compared to the computations with spin-orbit and are given for reference (columns highlighted in blue to emphasize this comparison). The axial value is given first. Equatorial values are averaged with standard deviations provided. The differences between the experimental and DFT values (δ_{SO}) are also reported.

Species	δ_{SR}	δ_{SO}	Δ_{SO}	Exp	Δ(DFT-Exp)
**UO-(NP^{pyrr})$_4$**	-75.9	-40.4	-35.5	-23.2	-17.2
	-41.2 ± 12.0	-9.2 ± 8.5	-32.0	0.6	-9.8
**UO-(NP^{pip})$_4$**	-72.3	-32.3	-40.1	-18.2	-14.1
	-38.8 ± 0.1	-5.6 ± 0.1	-33.3	9.9	-15.5
**UO-(NPC^1)$_4$**	-50.0	-19.6	-30.3	-7.0	-12.6
	-13.4 ± 1.5	13.9 ± 0.8	-27.4	21.2	-7.3
**UO-(NPC$^{1\text{-}pip}$)$_4$**	-45.3	-10.4	-34.9	-2.8	-7.6
	7.8 ± 1.9	35.1 ± 1.4	-27.3	25.4	+9.7

Table S12: Average ^{31}P chemical shifts in ppm for the H_3PO_4 acid reference and for the four complexes as computed on the TPSS geometry from Turbomole at the PBE0/TZP level of theory. Both the scalar-relativistic chemical shift, δ_{SR}, and the spin-orbit chemical shift, δ_{SO}, are given. The difference between the two is the spin-orbit shift, Δ_{SO}. Experimental values should be compared to the computations with spin-orbit and are given for reference (columns highlighted in blue to emphasize this comparison). The axial value is given first. Equatorial values are averaged with standard deviations provided. The differences between the experimental and DFT values (δ_{SO}) are also reported.

Species	δ_{SR}	δ_{SO}	Δ_{SO}	Exp	Δ(DFT-EXP)
**UO-(NP^{pyrr})$_4$**	-80.2	-36.9	-43.3	-23.2	-13.7
	-35.6 ± 12.2	-7.4 ± 7.9	-28.3	0.6	-8.0
**UO-(NP^{pip})$_4$**	-77.2	-28.0	-49.2	-18.2	-9.8
	-34.1 ± 0.1	-3.8 ± 0.1	-30.4	9.9	-13.7
**UO-(NPC^1)$_4$**	-54.8	-19.1	-35.7	-7.0	-12.1
	-9.4 ± 1.3	13.1 ± 0.6	-22.4	21.2	-8.1
**UO-(NPC$^{1\text{-}pip}$)$_4$**	-49.6	-7.9	-41.7	-2.8	-5.1
	10.8 ± 2.0	32.8 ± 1.3	-22.0	25.4	+7.4

Although the H_3PO_4 acid reference is used in experiment, prior literature suggests that errors associated with DFT arise in part its inability to compute the reference (e.g., $\sigma_{acid,ref}$), and not the complex of interest (e.g., $\sigma_{complex}$) accurately.[21, 22] Errors associated with calculating the H_3PO_4 acid reference directly with DFT can be contributing herein. When experimental data is available, secondary referencing can be used to apply an empirical correction to the computed values using the following equation:[23-25]

$$\delta_{calc} = \sigma_{secondary\text{-}ref} - \sigma_{complex} + \delta_{ref} \qquad (19)$$

where δ_{calc} is the calculated chemical shift with respect to the H_3PO_4 acid reference, $\sigma_{secondary\text{-}ref}$ is the calculated nuclear shielding constant for a secondary reference (presumably a molecule in which the DFT nuclear shielding constants result in less error in their computation), $\sigma_{complex}$ is the nuclear shielding constant for the complex of interest, and δ_{ref} is the experimental chemical shift measured for the secondary reference with respect to the H_3PO_4 acid reference.

First, PPh_3 was tested as an external reference. However, prior work has suggested that error cancelation can be facilitated with the NMR active nuclei, in this case P, being in a similar environment in the complex of interest and the secondary reference. Therefore, the free ligands were also measured and

employed as a secondary reference. Focusing only on values computed with our highest level of theory (PBE0, including spin-orbit coupling), comparisons are made to determine which free ligand should be used as the secondary reference in the DFT computation. The chemical shifts using this secondary reference approach for each of the free ligand or PPh_3 are computed with respect to the acid reference using **Equation (19)**. The experimental chemical shifts used in **Equation (19)** were -5.8, 28.44, 31.72, 47.49, and 51.29 ppm for PPh_3, **HNP^{pyrr}**, **HNP^{pip}**, **HNPC^{1}**, and **HNPC$^{1\text{-}pip}$**, respectively.

Computed chemical shifts are reported in the following tables using each of the free ligands, PPh_3, and PH_3 as the secondary reference (**Table S13**). REF-PPh3 uses PPh_3, REF-PH3 uses PH_3, REF-1 uses **HNP^{pyrr}**, REF-2 uses **HNP^{pip},** REF-3 uses **HNPC^{1}**, and REF-4 uses **HNPC$^{1\text{-}pip}$** as the secondary reference. The differences with experiment for each secondary reference choice are reported in **Table S14**, along with the maximum absolute difference, the minimum absolute difference, the average of the differences, and their standard deviation. The errors are smaller compared to the chemical shifts computed using the acid reference directly (via **Equation (18)**). Moreover, errors of similar magnitude are obtained for REF-1 to REF-4. However, the smallest errors are obtained for both REF-PH3 and REF-2. We remind readers that the quality of the computational is not impacted by the secondary reference choice, but improved agreement with experiment is observed due to favorable error cancelation. The PH_3 reference likely does well because it is one of the simplest possible ^{31}P containing molecules (minimizing structural contribution to chemical shift calculation based on inherent sensitivity of ^{31}P NMR shifts to local geometry at the P-atom) and the experimental measurement (–266.1 ppm)[26] is performed on the gas phase. On the other hand, the free ligand references do well because the P center is in a similar chemical environment in both the secondary reference and in the complex. We chose to include the results in the main text using the **HNP^{pip}** free ligand as the secondary reference, though we note that there is not a strong reason to choose one secondary reference over the other.

Table S13: The DFT calculated values computed using the secondary reference approach with a different choice of secondary reference. Computations are at the PBE0 level of theory including spin-orbit coupling. Specifically, $\delta_{REF\text{-}PPh3}$ uses PPh_3**,** $\delta_{REF\text{-}PH3}$ uses PH_3, $\delta_{REF\text{-}1}$ uses **HNP^{pyrr},** $\delta_{REF\text{-}2}$ uses **HNP^{pip},** $\delta_{REF\text{-}3}$ uses **HNPC^{1}**, and $\delta_{REF\text{-}4}$ uses **HNPC$^{1\text{-}pip}$** as the secondary reference.

Species	$\delta_{REF\text{-}PPh3}$	$\delta_{REF\text{-}PH3}$	$\delta_{REF\text{-}1}$	$\delta_{REF\text{-}2}$	$\delta_{REF\text{-}3}$	$\delta_{REF\text{-}4}$	Exp
**UO-(NP^{pyrr})$_4$**	-32.6	-28.5	-23.8	-27.1	-23.1	-24.8	-23.2
	-3.1 ± 7.9	1.0 ± 7.9	5.7 ± 7.9	2.4 ± 7.9	6.4 ± 7.9	4.8 ± 7.9	0.6
**UO-(NP^{pip})$_4$**	-23.7	-19.6	-14.9	-18.2	-14.2	-15.8	-18.2
	0.6 ± 0.1	4.7 ± 0.1	9.4 ± 0.1	6.0 ± 0.1	10.1 ± 0.1	8.4 ± 0.1	9.9
**UO-(NPC^{1})$_4$**	-14.8	-10.7	-6.0	-9.3	-5.3	-6.9	-7.0
	17.4 ± 0.6	21.5 ± 0.6	26.2 ± 0.6	22.9 ± 0.6	26.9 ± 0.6	25.2 ± 0.6	21.2
**UO-(NPC$^{1\text{-}pip}$)$_4$**	-3.6	0.5	-5.2	1.9	5.9	4.3	-2.8
	37.1 ± 1.3	41.2 ± 1.3	45.9 ± 1.3	42.6 ± 1.3	46.6 ± 1.3	45.0 ± 1.3	25.4

Table S14: The difference between experiment and the values obtained using the secondary reference approach with a different choice of secondary reference. Computations are at the PBE0 level of theory including spin-orbit coupling. Specifically, $\Delta_{exp\text{-}REF\text{-}PPh3}$ uses PPh_3, $\Delta_{exp\text{-}REF\text{-}PH3}$ uses PH_3, $\Delta_{exp\text{-}REF\text{-}1}$ uses **HNP^{pyrr}**, $\Delta_{exp\text{-}REF\text{-}2}$ uses **HNP^{pip}**, $\Delta_{exp\text{-}REF\text{-}3}$ uses **HNPC^{1}**, and $\Delta_{exp\text{-}REF\text{-}4}$ uses **HNPC$^{1\text{-}pip}$** as the secondary reference. The maximum and minimum absolute errors are reported, along with the average error and its standard deviation. The errors including all values are reported first, while the errors in parentheses exclude the large error associated with the equatorial position of **UO-(NPC$^{1\text{-}pip}$)$_4$**.

Species	$\Delta_{exp\text{-}REF\text{-}PPh3}$	$\Delta_{exp\text{-}REF\text{-}PH3}$	$\Delta_{exp\text{-}REF\text{-}1}$	$\Delta_{exp\text{-}REF\text{-}2}$	$\Delta_{exp\text{-}REF\text{-}3}$	$\Delta_{exp\text{-}REF\text{-}4}$
**UO-(NP^{pyrr})$_4$**	9.4	5.3	0.6	4.0	-0.1	1.6
	3.7	-0.4	-5.1	-1.8	-5.8	-4.2
**UO-(NP^{pip})$_4$**	5.5	1.4	-3.3	0.0	-4.0	-2.4
	9.3	5.2	0.5	3.9	-0.2	1.5
**UO-(NPC^{1})$_4$**	7.8	3.7	-1.0	2.3	-1.7	-0.1
	3.8	-0.3	-5.0	-1.7	-5.7	-4.0
**UO-(NPC$^{1\text{-}pip}$)$_4$**	0.8	-3.3	2.4	-4.7	-8.7	-7.1
	-11.7	-15.8	-20.5	-17.2	-21.2	-19.6
**Max Abs. Error**	11.7 (9.4)	15.8 (5.3)	20.5 (5.1)	17.2 (4.7)	21.2 (8.7)	19.6 (7.0)
**Min Abs. Error**	0.8 (0.8)	0.3 (0.3)	0.5 (0.5)	0.0 (0.0)	0.1 (0.1)	0.1 (0.1)
**Avg Error**	3.6 (5.8)	-0.5 (1.7)	-3.9 (-1.6)	-1.9 (0.3)	-5.9 (-3.7)	-4.3 (-2.1)
**St. Dev.**	6.9 (3.2)	6.9 (3.2)	7.2 (3.0)	6.9 (3.3)	6.9 (3.2)	6.9 (3.3)

Table S15: Average ^{31}P chemical shifts in ppm for the four complexes as computed on the TPSS geometry from Turbomole at the PBE/TZP level of theory. The **HNP^{pip}** ligand is used as the secondary reference and chemical shifts are computed using **Equation (19)**. Both the scalar-relativistic chemical shift, δ_{SR}, and the spin-orbit chemical shift, δ_{SO}, are given. The difference between the two is the spin-orbit shift, Δ_{SO}. Experimental values should be compared to the computations with spin-orbit and are given for reference (columns highlighted in blue to emphasize this comparison). The axial value is given first. Equatorial values are averaged with standard deviations provided. The difference between the experimental and DFT values (δ_{SO}) are also reported.

Species	δ_{SR}	δ_{SO}	Δ_{SO}	Exp	Δ(DFT-Exp)
**UO-(NP^{pyrr})$_4$**	-61.6	-15.3	-46.3	-23.2	-7.9
	-26.9 ± 12.0	15.9 ± 8.5	-42.8	0.6	-15.3
**UO-(NP^{pip})$_4$**	-58.0	-7.2	-50.8	-18.2	-11.0
	-24.5 ± 0.1	19.5 ± 0.1	-44.0	9.9	-9.6
**UO-(NPC^{1})$_4$**	-35.6	5.4	-41.1	-7.0	-12.4
	0.9 ± 1.5	39.0 ± 0.8	-38.1	21.2	-17.8
**UO-(NPC$^{1\text{-}pip}$)$_4$**	-30.9	14.7	-45.6	-2.8	-17.5
	22.1 ± 1.9	60.1 ± 1.4	-38.0	25.4	-34.7

Table S16: Average ^{31}P chemical shifts in ppm for the four complexes as computed on the TPSS geometry from Turbomole at the PBE0/TZP level of theory. The **HNP^{pip}** ligand is used as the secondary reference and chemical shifts are computed using **Equation (19)**. Both the scalar-relativistic chemical shift, δ_{SR}, and the spin-orbit chemical shift, δ_{SO}, are given. The difference between the two is the spin-orbit shift, Δ_{SO}. Experimental values should be compared to the computations with spin-orbit and are given for reference (columns highlighted in blue to emphasize this comparison). The axial value is given first. Equatorial values are averaged with standard deviations provided. The difference between the experimental and DFT values (δ_{SO}) are also reported.

Species	δ_{SR}	δ_{SO}	Δ_{SO}	Exp	Δ(DFT-Exp)
**UO-(NP^{pyrr})$_4$**	-68.3	-27.1	-41.2	-23.2	+4.0
	-23.7 ± 12.0	2.4 ± 7.9	-25.9	0.6	-1.8
**UO-(NP^{pip})$_4$**	-65.3	-18.2	-46.9	-18.2	+0.0
	-22.2 ± 0.1	6.0 ± 0.1	-28.1	9.9	+3.9
**UO-(NPC^1)$_4$**	-42.9	-9.3	-33.4	-7.0	+2.3
	-2.5 ± 1.3	22.9 ± 0.6	-20.1	21.2	-1.7
**UO-(NPC$^{1\text{-}pip}$)$_4$**	-37.3	1.9	-39.4	-2.8	-4.7
	22.7 ± 2.0	42.6 ± 1.3	-19.7	25.4	-17.2

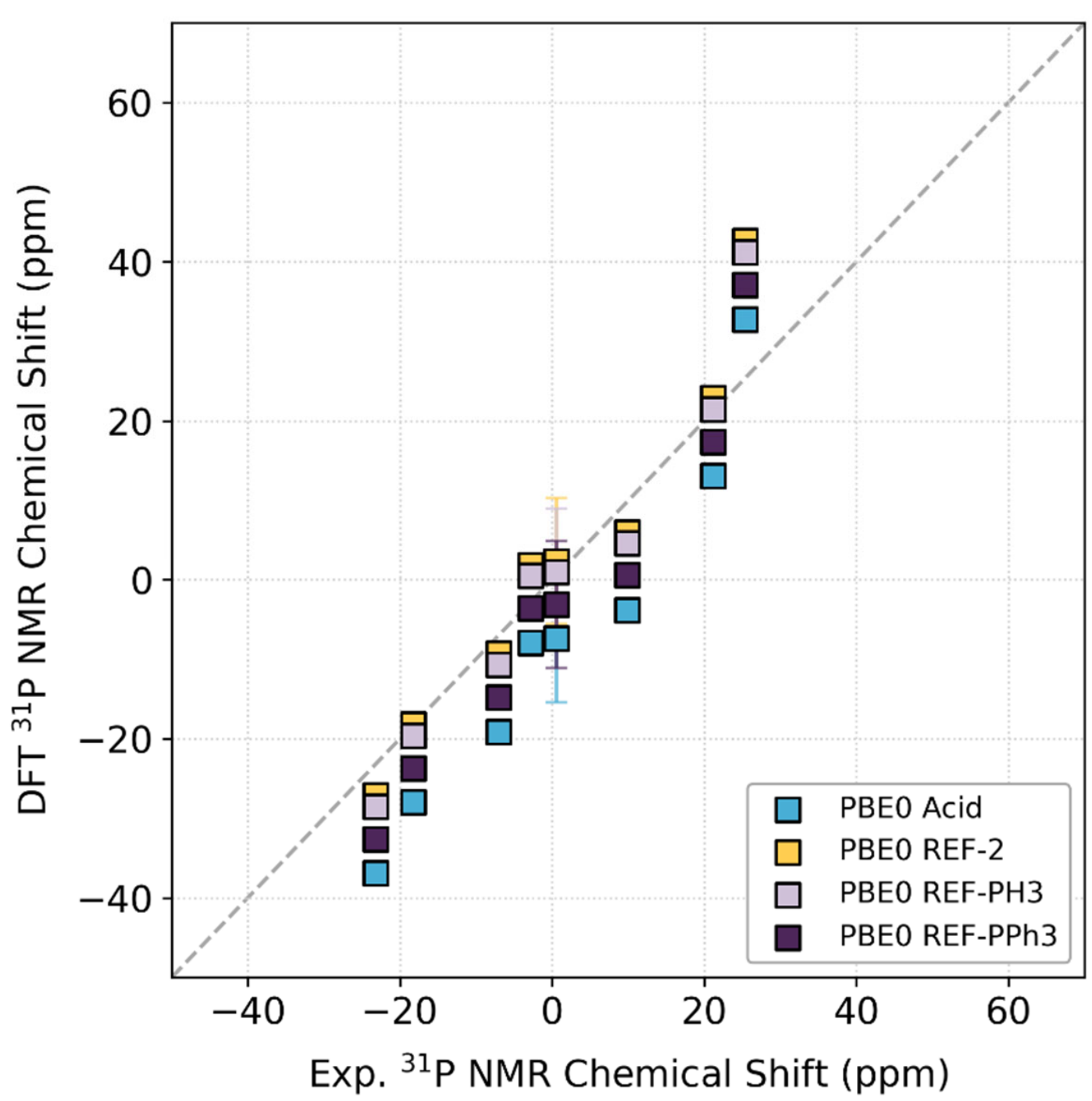

Figure S61: Scatter plot of the experimental ^{31}P chemical shifts vs the DFT values for both the axial and equatorial ligand in ppm. Values with the acid reference are in blue. Values with the **HNP^{pip}** ligand as the secondary reference (REF-2) are in yellow. Values with the PH_3 as the secondary reference (Ref-PH_3) are in light purple. Values with the PPh_3 as the secondary reference (Ref-PPh_3) are in dark purple. DFT values are computed on the TPSS geometry from Turbomole at the PBE0/TZP (squares) levels of theory. The PBE0 values with REF-2 and REF-PH3 are closest to the ideal agreement (shown with the dashed gray line).

7.4 Natural Localized Molecular Orbitals

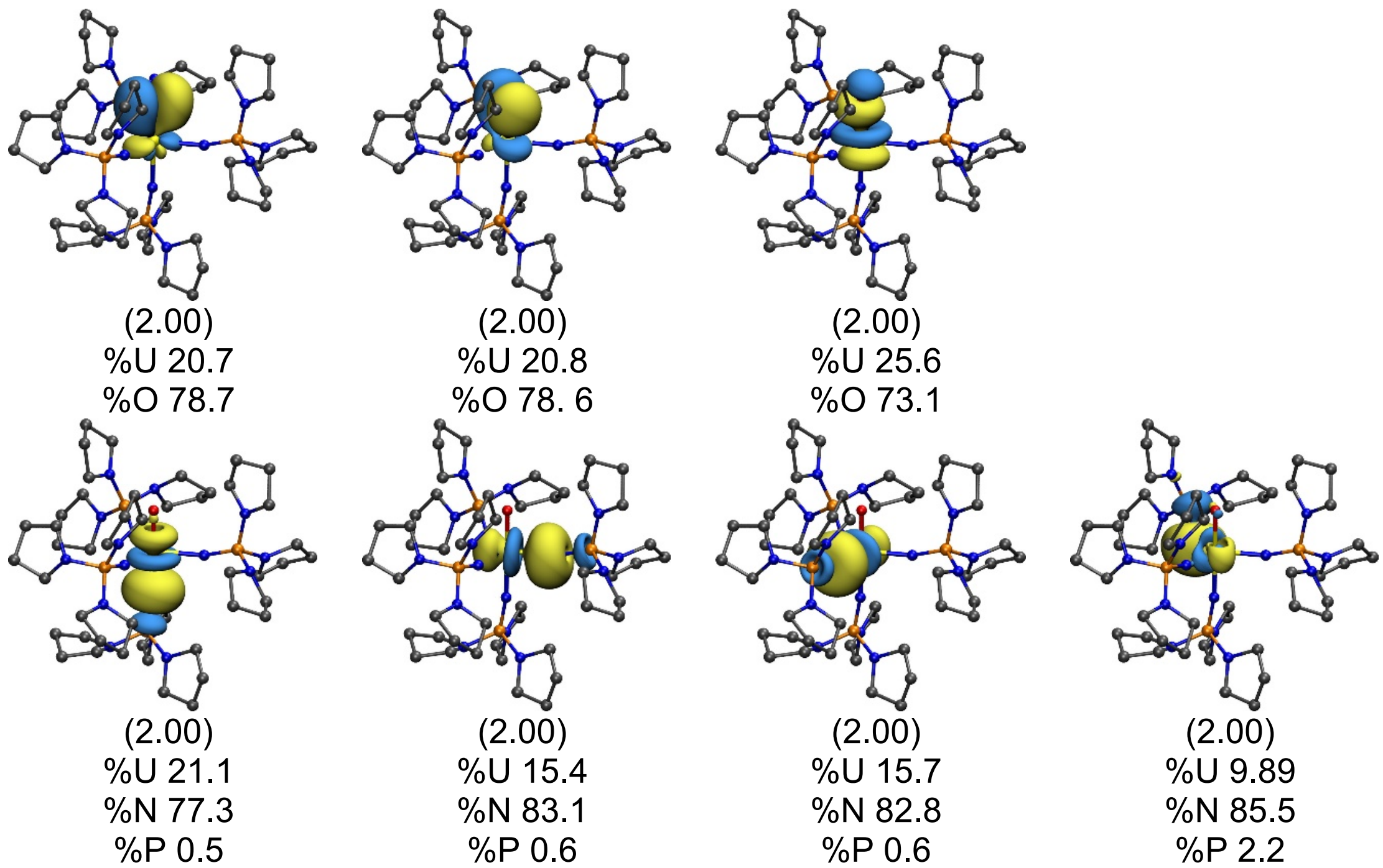

Figure S62: Selected natural localized molecular orbitals (NLMOs) and their corresponding occupation numbers (in parentheses) from **UO-(NP^{pyrr})$_4$**. Computed with PBE/TZP on the TPSS optimized geometry. Percent contributions for U and O are provided for the U=O bonds (top), while U, N, and P contributions are included for the σ contributions to the U-N bonds (bottom).

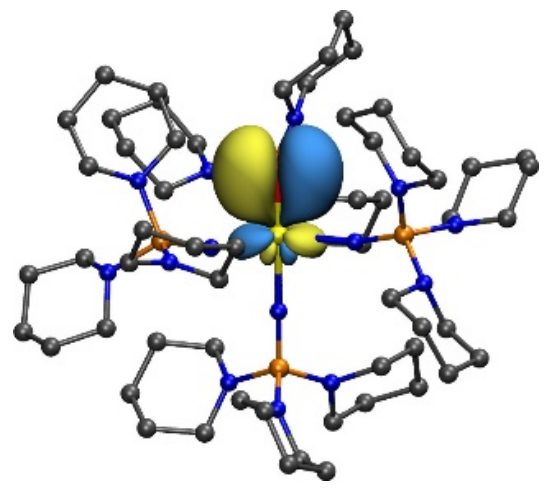

(2.00)
%U 20.2
%O 79.0

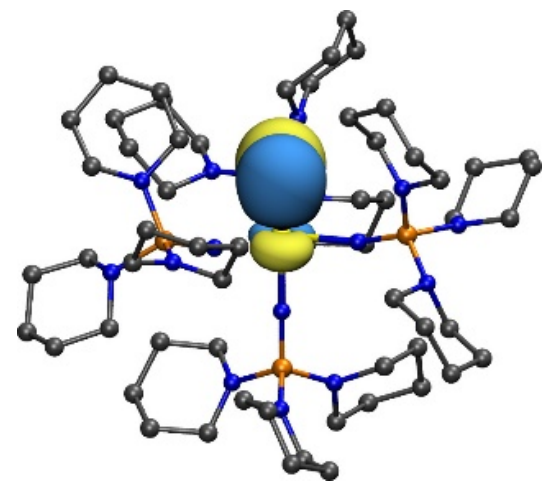

(2.00)
%U 20.3
%O 78.9

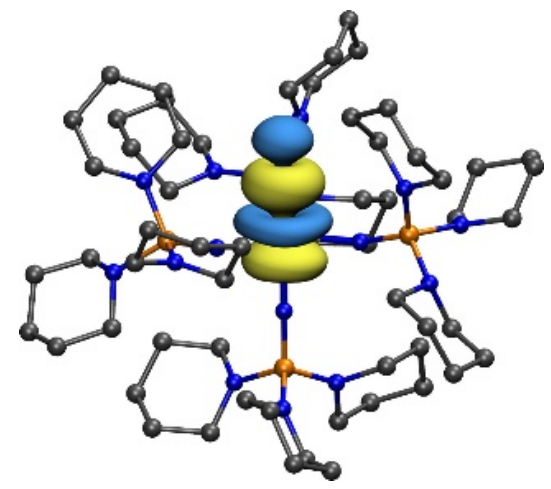

(2.00)
%U 26.0
%O 72.5

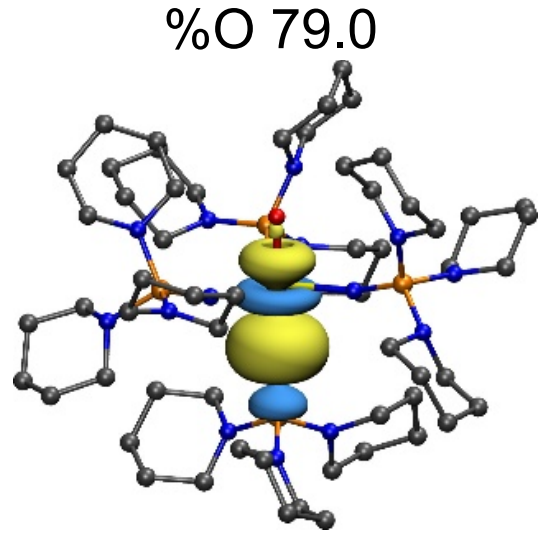

(2.00)
%U 20.3
%N 77.9
%P 0.5

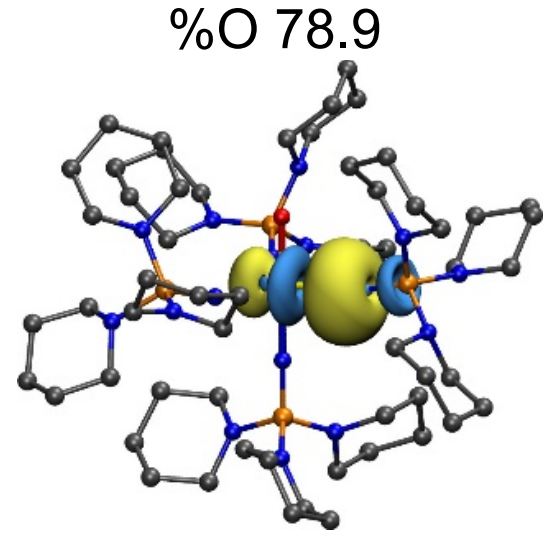

(2.00)
%U 14.3
%N 84.2
%P 0.7

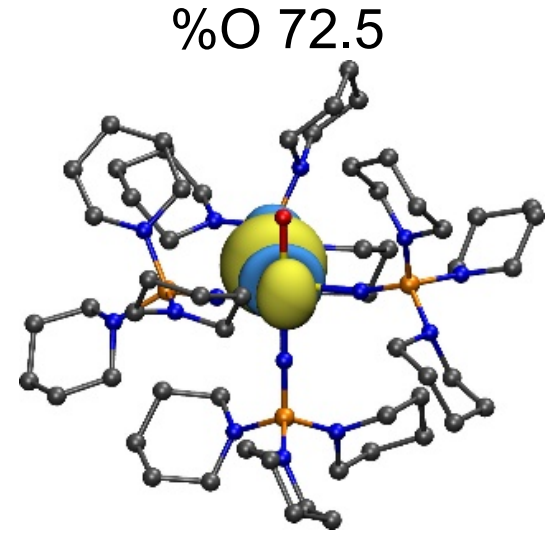

(2.00)
%U 14.3
%N 84.1
%P 0.7

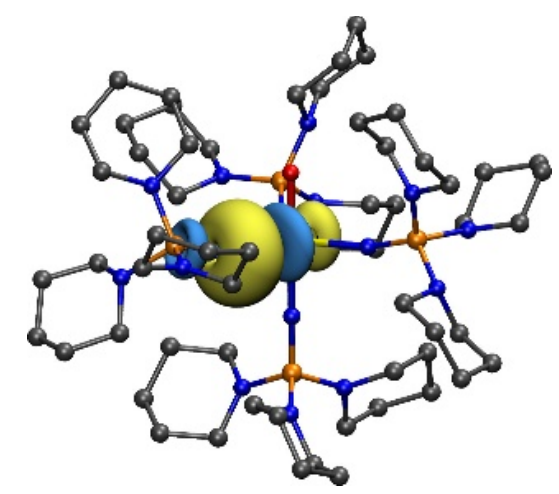

(2.00)
%U 14.3
%N 84.1
%P 0.7

Figure S63: Selected natural localized molecular orbitals (NLMOs) and their corresponding occupation numbers (in parentheses) from **UO-(NP^{pip})$_4$.** Computed with PBE/TZP on the TPSS optimized geometry. Percent contributions for U and O are provided for the U=O bonds (top), while U, N, and P contributions are included for the σ contributions to the U-N bonds (bottom).

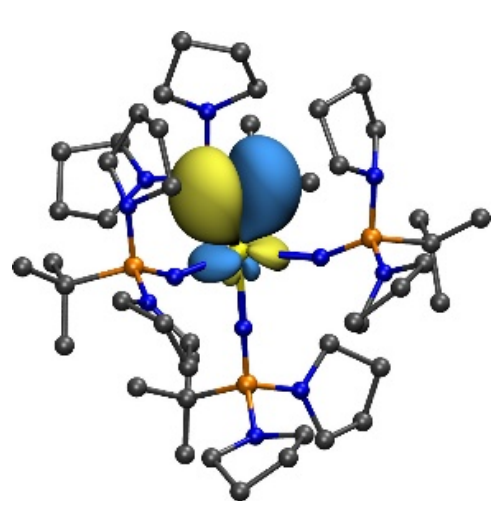

(2.00)
%U 18.6
%O 80.7

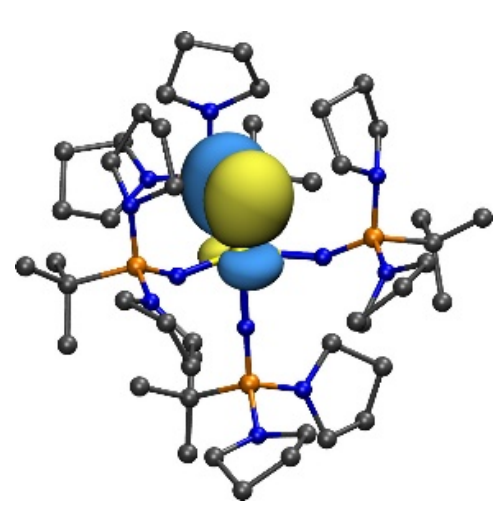

(2.00)
%U 18.6
%O 80.7

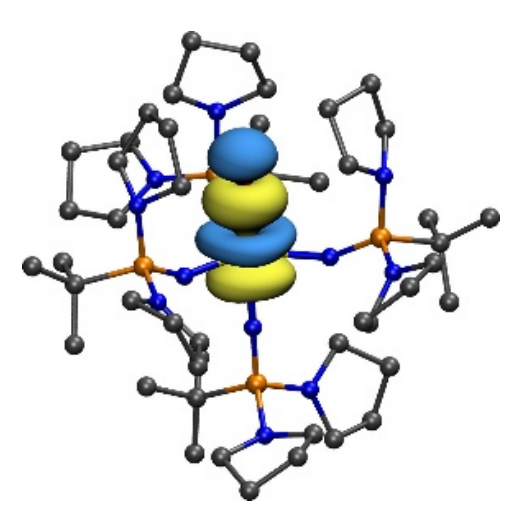

(2.00)
%U 24.7
%O 73.7

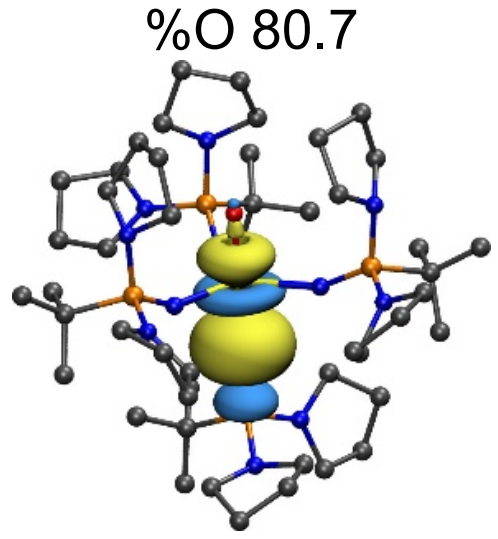

(2.00)
%U 17.3
%N 80.7
%P 0.7

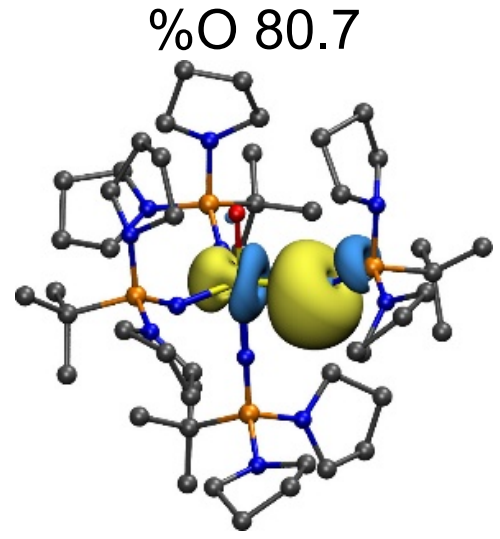

(2.00)
%U 8.1
%N 88.9
%P 1.1

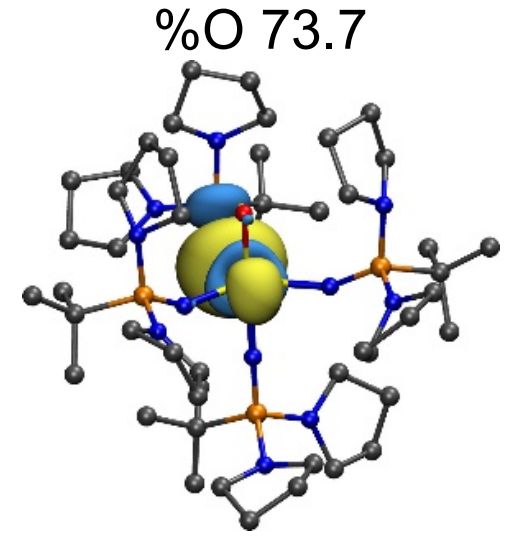

(2.00)
%U 8.0
%N 88.8
%P 1.1

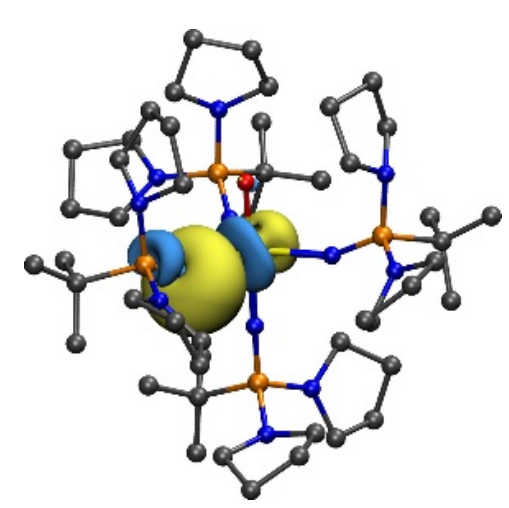

(2.00)
%U 8.1
%N 88.8
%P 1.1

Figure S64: Selected natural localized molecular orbitals (NLMOs) and their corresponding occupation numbers (in parentheses) from **UO-(NPC^1)$_4$.** Computed with PBE/TZP on the TPSS optimized geometry. Percent contributions for U and O are provided for the U=O bonds (top), while U, N, and P contributions are included for the σ contributions to the U-N bonds (bottom).

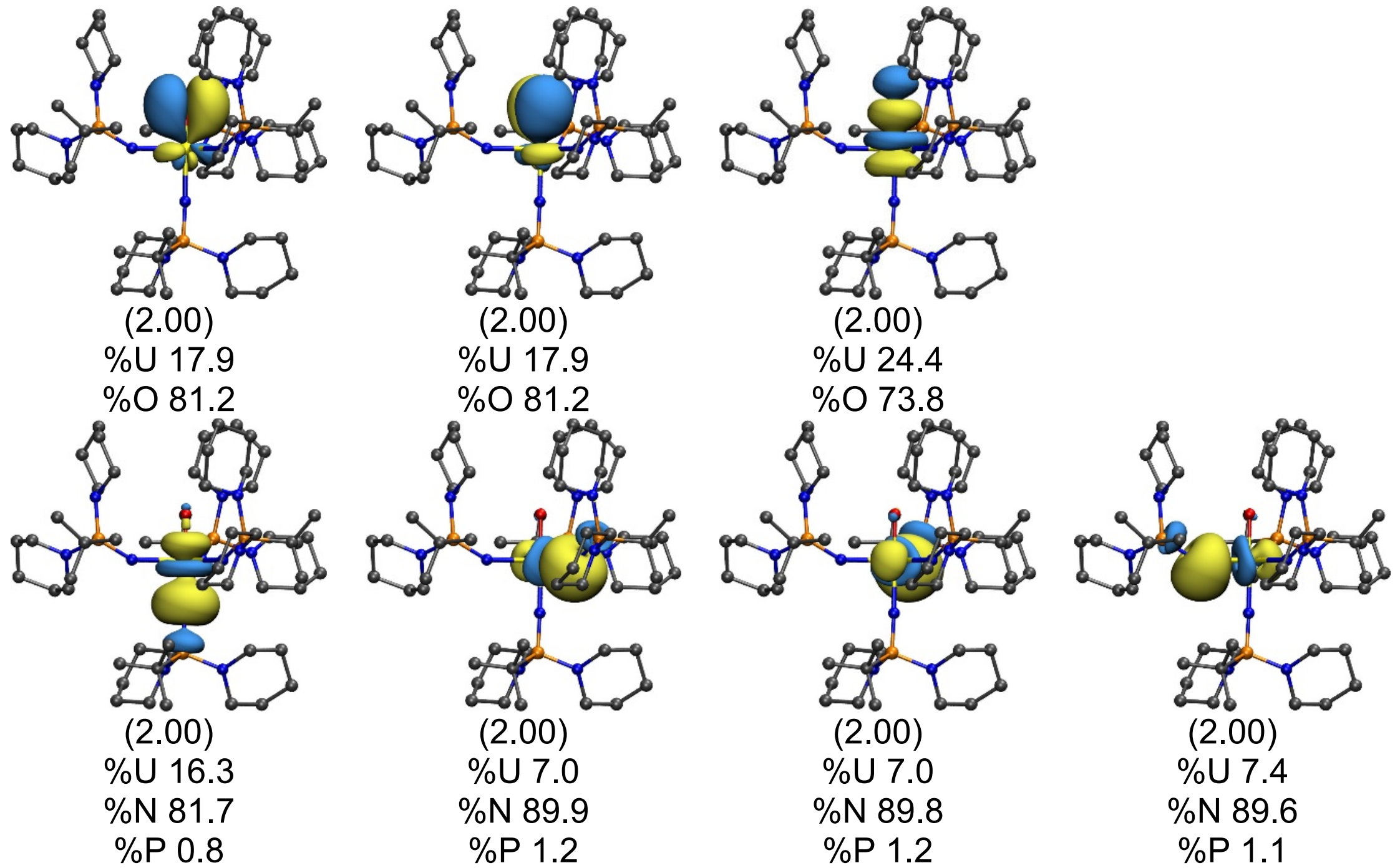

Figure S65: Selected natural localized molecular orbitals (NLMOs) and their corresponding occupation numbers (in parentheses) from **UO-(NPC$^{1\text{-pip}}$)$_4$**. Computed with PBE/TZP on the TPSS optimized geometry. Percent contributions for U and O are provided for the U=O bonds (top), while U, N, and P contributions are included for the σ contributions to the U-N bonds (bottom).

Table S17: Atomic orbital contributions to the uranium contribution to the NLMOS for the U=O σ bond. The total contribution from uranium is also given.

	U in U=O orbital				
	**s**	**p**	**d**	**f**	**Total**
**UO-(NP^{pyrr})$_4$**	0.13	0.12	16.15	83.60	25.6
**UO-(NP^{pip})$_4$**	0.26	0.17	15.41	84.15	26.0
**UO-(NPC^{1})$_4$**	0.40	0.20	9.21	90.19	24.7
**UO-(NPC$^{1\text{-pip}}$)$_4$**	0.43	0.25	8.48	90.84	24.4

Table S18: Atomic orbital contributions to the uranium contribution to the NLMOS for the U-N σ bond in the axial and equatorial ligands. The total contribution from uranium is also given for each orbital.

	U in axial U-N bond					U in equat. U-N bond				
	**s**	**p**	**d**	**f**	**Total**	**s**	**p**	**d**	**f**	**Total**
**UO-(NP^{pyrr})$_4$**	3.66	0.04	31.73	64.56	21.1	7.14	0.09	57.50	35.26	13.6
**UO-(NP^{pip})$_4$**	0.74	0.05	32.77	66.44	20.3	0.90	0.11	64.27	34.72	14.3
**UO-(NPC^{1})$_4$**	0.35	0.08	20.44	79.13	17.3	0.44	0.42	51.44	47.70	8.1
**UO-(NPC$^{1\text{-pip}}$)$_4$**	0.47	0.11	16.96	82.46	16.3	0.49	0.61	47.29	51.60	7.2

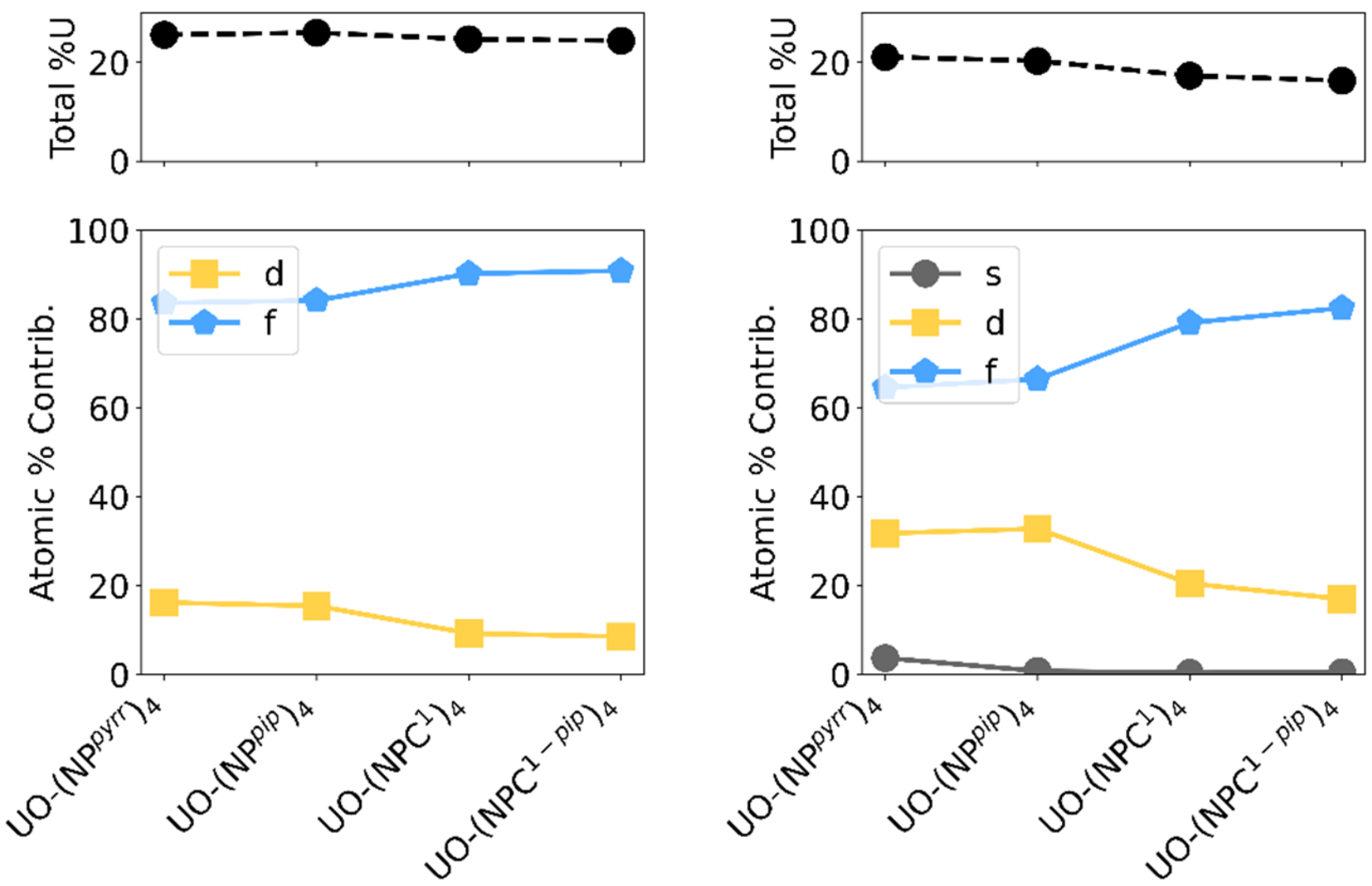

Figure S66: Decomposition of the U contribution to the U=O σ bond (left) and the axial U-N σ bond (right). The total percent U contribution is shown in the top plot, while the atomic contributions from uranium are plotted below. Contributions from p orbitals to both NLMOs are very small (**Table S17** and **S18**) and are not plotted in either graph. Likewise, the small contributions from the s orbitals are not included in the left plot.

7.5 EDA and ETS-NOCV Results

Table S19: Energy decomposition analysis results for the axial ligand (TPSS/TZP on the TPSS geometry). The steric interaction is the sum of the Pauli repulsion and the electrostatic contribution. The percent electrostatic and orbitalic interactions are of the total attractive energy. Energies in kcal/mol.

	INT E	Pauli	Elect	Orb	%Elec	%Orb	Steric
**UO-(NP^{pyrr})$_4$**	-157.1	243.5	-234.8	-165.8	58.6	41.4	8.7
**UO-(NP^{pip})$_4$**	-160.6	243.9	-235.4	-169.1	58.2	41.8	8.6
**UO-(NPC^{1})$_4$**	-159.7	243.9	-235.1	-168.5	58.3	41.7	8.8
**UO-(NPC$^{1\text{-}pip}$)$_4$**	-157.9	243.2	-233.0	-168.1	58.1	41.9	10.2

Table S20: Energy decomposition analysis results for an equatorial ligand (TPSS/TZP on the TPSS geometry). The steric interaction is the sum of the Pauli repulsion and the electrostatic contribution. The percent electrostatic and orbitalic interactions are of the total attractive energy. Energies in kcal/mol.

	INT E	Pauli	Elect	Orb	%Elec	%Orb	Steric
**UO-(NP^{pyrr})$_4$**	-149.3	174.3	-203.8	-119.9	63.0	37.0	-29.4
**UO-(NP^{pip})$_4$**	-147.8	170.9	-199.6	-119.1	62.6	37.4	-28.7
**UO-(NPC^{1})$_4$**	-146.7	166.1	-198.5	-114.2	63.5	36.5	-32.4
**UO-(NPC$^{1\text{-}pip}$)$_4$**	-145.9	172.1	-200.4	-117.7	63.0	37.0	-28.3

Table S21: Energy decomposition analysis results for the axial ligand (TPSS-D3(BJ)/TZP on the TPSS geometry). The steric interaction is the sum of the Pauli repulsion and the electrostatic contribution. The percent electrostatic, orbitalic, and dispersion interactions are of the total attractive energy. Energies in kcal/mol.

	INT E	Pauli	Elect	Orb	Disp	%Elec	%Orb	%Disp	Steric
**UO-(NP^{pyrr})$_4$**	-180.9	243.5	-234.8	-165.8	-23.77	55.3	39.1	5.6	8.66
**UO-(NP^{pip})$_4$**	-186.4	243.9	-235.4	-169.1	-25.79	54.7	39.3	6.0	8.55
**UO-(NPC^{1})$_4$**	-182.4	243.2	-233.0	-168.1	-24.49	54.7	39.5	5.8	10.19
**UO-(NPC$^{1\text{-}pip}$)$_4$**	-182.4	243.2	-233.0	-168.1	-24.49	54.7	39.5	5.8	10.19

Table S22: Energy decomposition analysis results for an equatorial ligand (TPSS-D3(BJ)/TZP on the TPSS geometry). The steric interaction is the sum of the Pauli repulsion and the electrostatic contribution. The percent electrostatic, orbitalic, and dispersion interactions are of the total attractive energy. Energies in kcal/mol.

	INT E	Pauli	Elect	Orb	Disp	%Elec	%Orb	%Disp	Steric
**UO-(NP^{pyrr})$_4$**	-167.6	174.3	-203.8	-119.9	-18.29	59.6	35.1	5.3	-29.43
**UO-(NP^{pip})$_4$**	-167.7	170.9	-199.6	-119.1	-19.84	59.0	35.2	5.9	-28.68
**UO-(NPC^{1})$_4$**	-167.5	166.1	-198.5	-114.2	-20.80	59.5	34.3	6.2	-32.44
**UO-(NPC$^{1\text{-}pip}$)$_4$**	-168.0	172.1	-200.4	-117.7	-22.09	58.9	34.6	6.5	-28.25

Table S23: ETS-NOCV analysis results for an axial ligand (TPSS/TZP on the TPSS geometry). NOCV pair orbital interaction energies are given for the strongest three interactions (all others are smaller than -6 kcal/mol). For each pair, the NOCV eigenvalue, $|v_e|$, which corresponds to the number of electrons transferred is given in parentheses. The total of all orbital interaction energies is also reported. Energies in kcal/mol.

	**UO-(NP^{pyrr})$_4$**	**UO-(NP^{pip})$_4$**	**UO-(NPC^{1})$_4$**	**UO-(NPC$^{1\text{-}pip}$)$_4$**
**Axial**	-59.4 (0.75)	-61.2 (0.75)	-61.3 (0.76)	-61.2 (0.75)
	-30.9 (0.69)	-30.6 (0.69)	-31.5 (0.70)	-31.7 (0.69)
	-30.4 (0.67)	-30.6 (0.69)	-31.2 (0.67)	-30.5 (0.67)
Total	*-162.8*	*-166.0*	*-165.9*	*-166.3*
**Equat.**	-35.1 (0.64)	-34.2 (0.64)	-33.9 (0.65)	-36.7 (0.67)
	-23.2 (0.62)	-22.5 (0.61)	-21.7 (0.58)	-22.4 (0.60)
	-25.6 (0.52)	-25.6 (0.52)	-24.2 (0.51)	-22.9 (0.49)
Total:	*-117.1*	*-116.2*	*-111.5*	*-115.3*

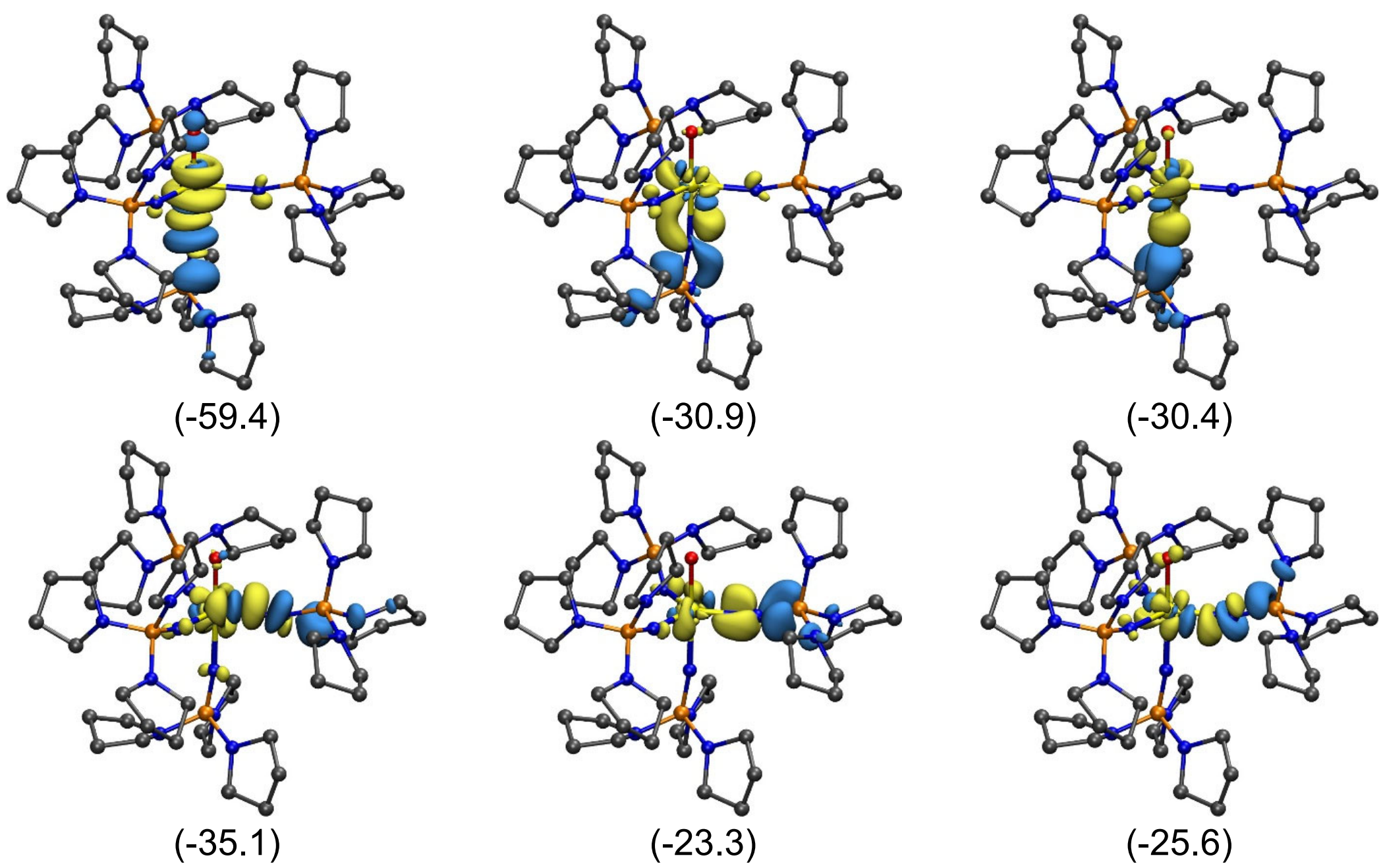

Figure S67: NOCV deformation densities for the axial (top row) and equatorial (bottom row) ligands in **UO-(NP^{pyrr})$_4$.** Energies are given in kcal/mol and in parentheses. An isosurface of 0.003 a.u. is used. Blue indicates regions of charge depletion, and yellow indicates regions of charge accumulation.

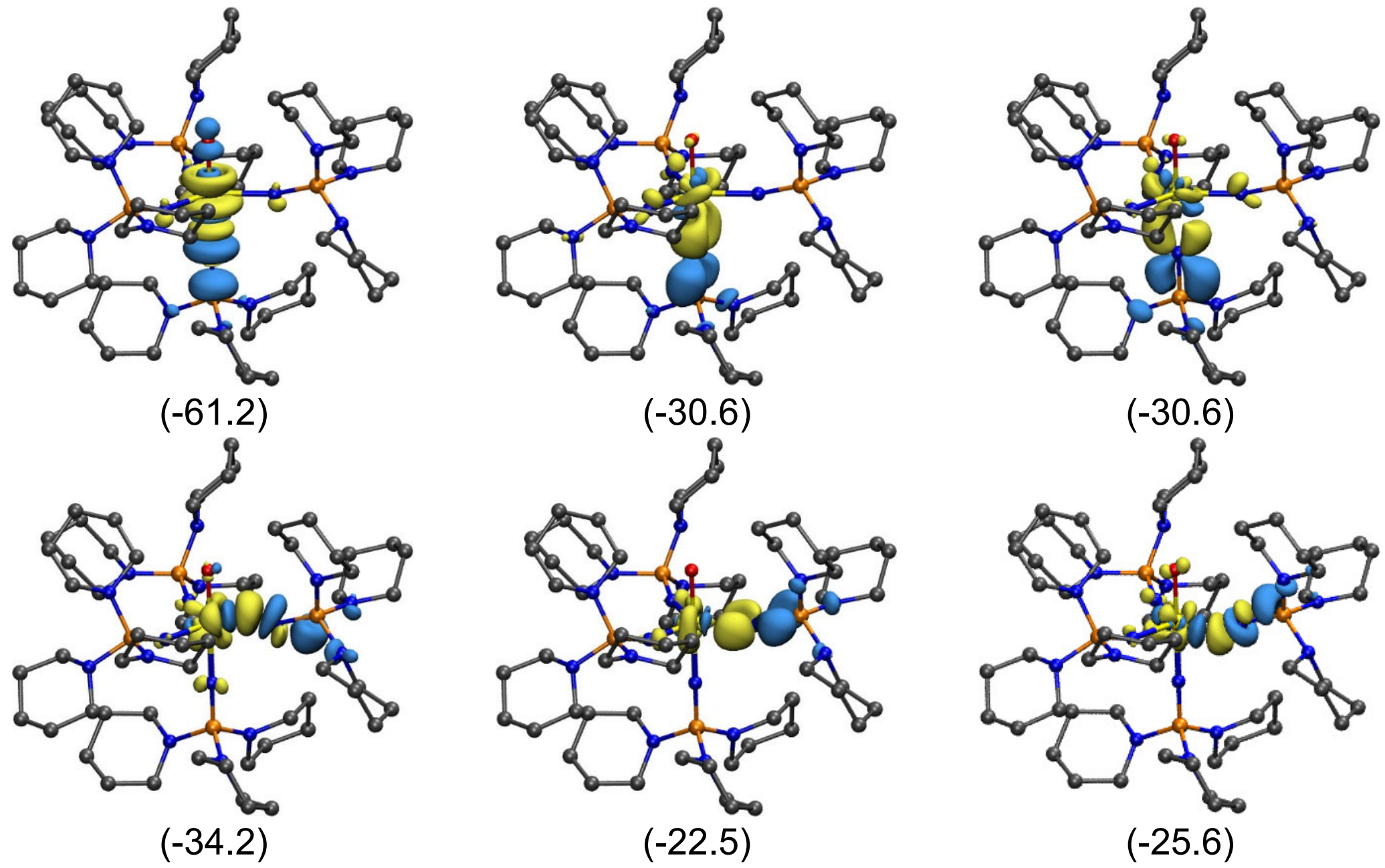

Figure S68: NOCV deformation densities for the axial (top row) and equatorial (bottom row) ligands in **UO-(NP^{pip})$_4$.** Energies are given in kcal/mol and in parentheses. An isosurface of 0.003 a.u. is used. Blue indicates regions of charge depletion, and yellow indicates regions of charge accumulation.

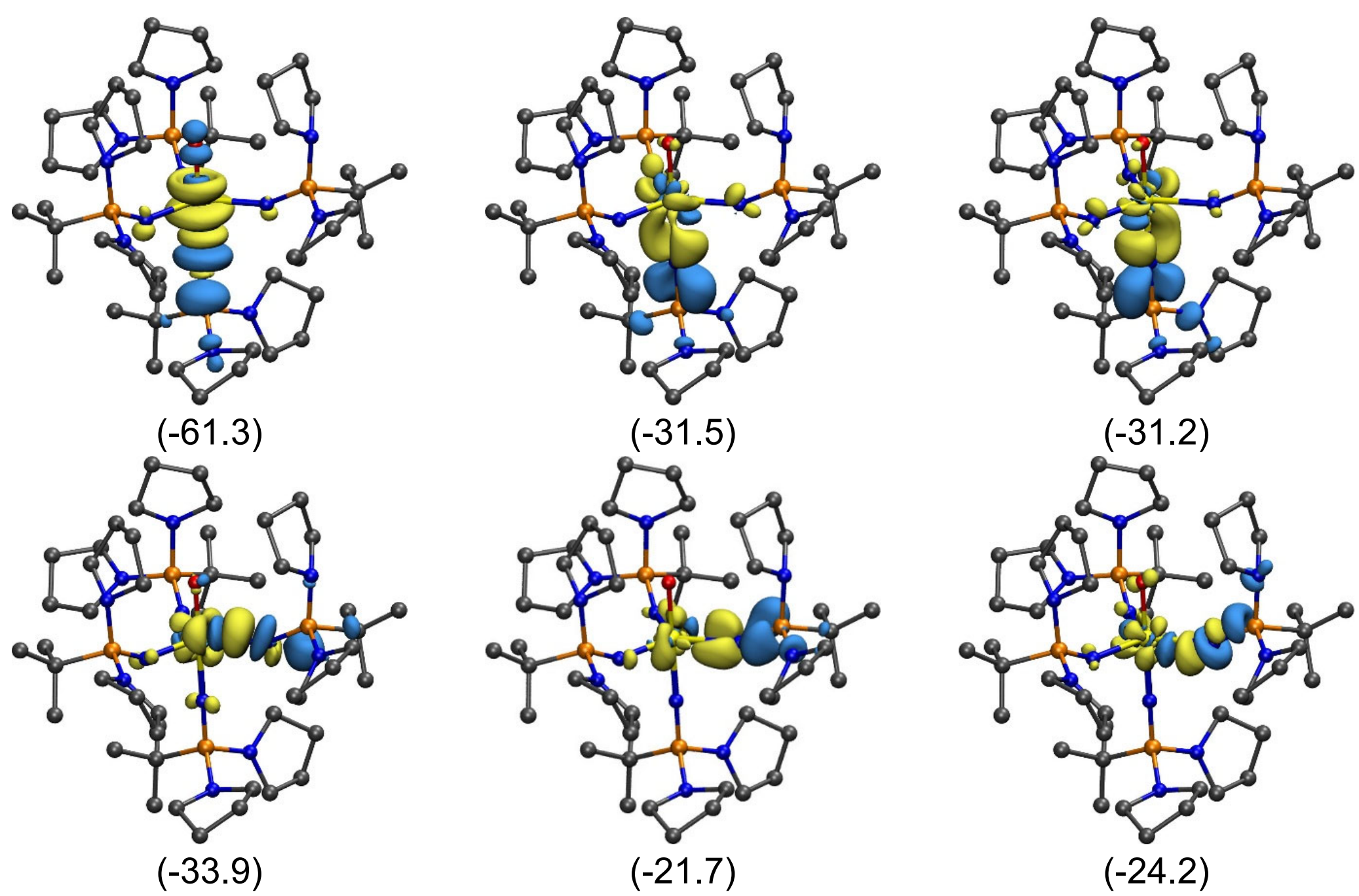

Figure S69: NOCV deformation densities for the axial (top row) and equatorial (bottom row) ligands in **UO-(NPC^1)$_4$.** Energies are given in kcal/mol and in parentheses. An isosurface of 0.003 a.u. is used. Blue indicates regions of charge depletion, and yellow indicates regions of charge accumulation.

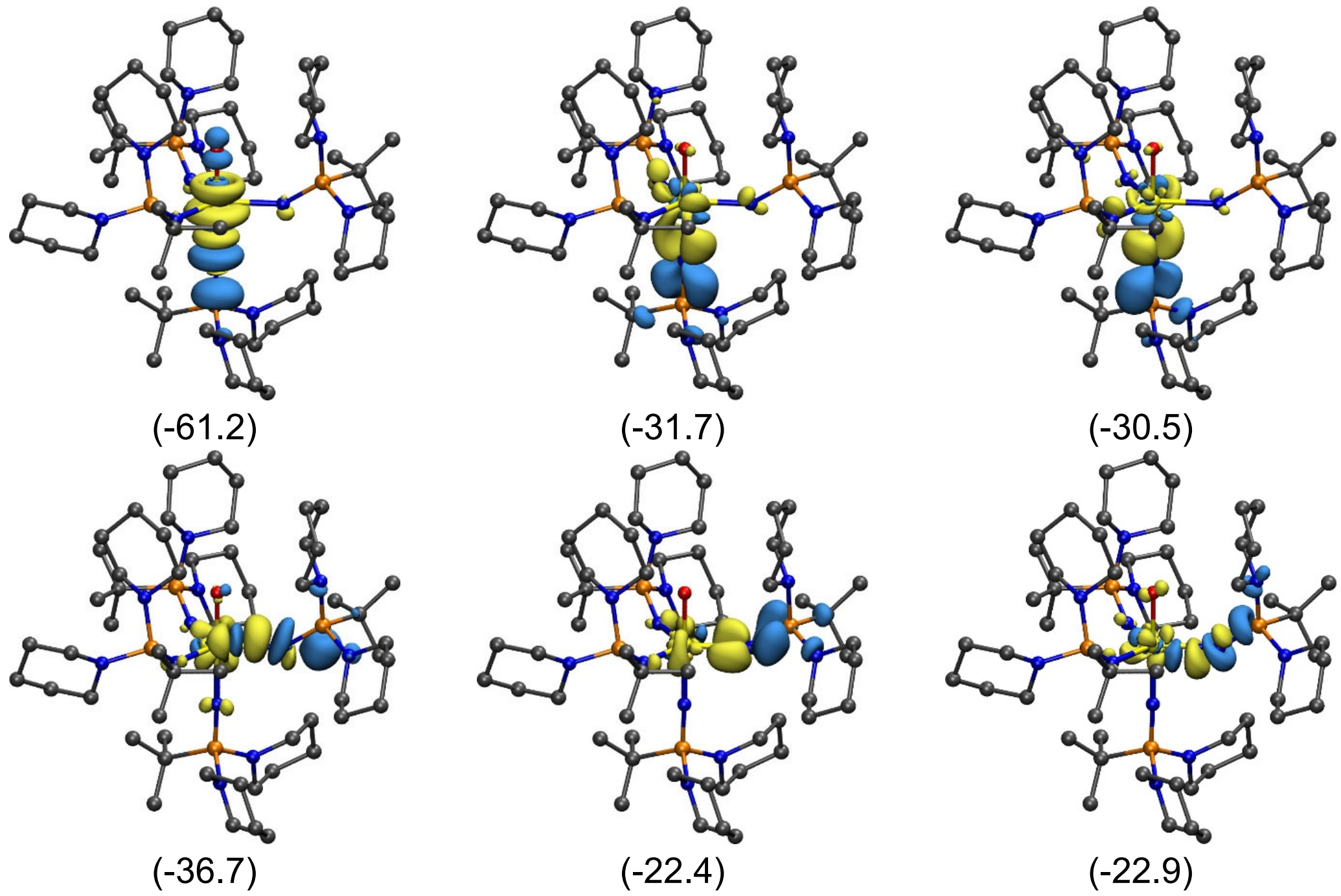

Figure S70: NOCV deformation densities for the axial (top row) and equatorial (bottom row) ligands in complex **UO-(NPC$^{1\text{-pip}}$)$_4$.** Energies are given in kcal/mol and in parentheses. An isosurface of 0.003 a.u. is used. Blue indicates regions of charge depletion, and yellow indicates regions of charge accumulation.

Table S24: QTAIM parameters (TPSS) at the bond critical points (BCPs) along the U=O, U-N$_{axial}$, and U-N$_{equat}$ bonds. Values for the equatorial position are reported as an average of the three bonds. The bond degree (BD) is the total electron energy density (H) normalized by electron density (**ρ**) at the BCP.

Bond	Species	ρ	∇ρ	Ellipticity	G(r)	V(r)	H(r)	|V|/G	BD
**U=O**	**UO-(NP^{pyrr})$_4$**	0.246	0.411	0.009	0.346	-0.588	-0.243	1.703	-0.988
	**UO-(NP^{pip})$_4$**	0.247	0.411	0.001	0.348	-0.594	-0.246	1.705	-0.993
	**UO-(NPC^1)$_4$**	0.247	0.411	0.001	0.348	-0.594	-0.246	1.705	-0.993
	**UO-(NPC$^{1\text{-pip}}$)$_4$**	0.248	0.411	0.001	0.349	-0.595	-0.246	1.705	-0.994
**U-N$_{axial}$**	**UO-(NP^{pyrr})$_4$**	0.137	0.295	0.015	0.154	-0.234	-0.080	1.521	-0.585
	**UO-(NP^{pip})$_4$**	0.137	0.300	0.000	0.155	-0.235	-0.080	1.516	-0.582
	**UO-(NPC^1)$_4$**	0.137	0.300	0.000	0.155	-0.235	-0.080	1.516	-0.582
	**UO-(NPC$^{1\text{-pip}}$)$_4$**	0.136	0.300	0.016	0.153	-0.231	-0.078	1.509	-0.574
**U-N$_{equat}$**	**UO-(NP^{pyrr})$_4$**	0.110	0.306	0.089	0.123	-0.170	-0.047	1.379	-0.426
	**UO-(NP^{pip})$_4$**	0.108	0.308	0.091	0.122	-0.167	-0.045	1.369	-0.416
	**UO-(NPC^1)$_4$**	0.108	0.308	0.091	0.122	-0.167	-0.045	1.369	-0.416
	**UO-(NPC$^{1\text{-pip}}$)$_4$**	0.108	0.295	0.117	0.120	-0.165	-0.046	1.383	-0.424

7.6 Selected Structure Proposed as Barrier

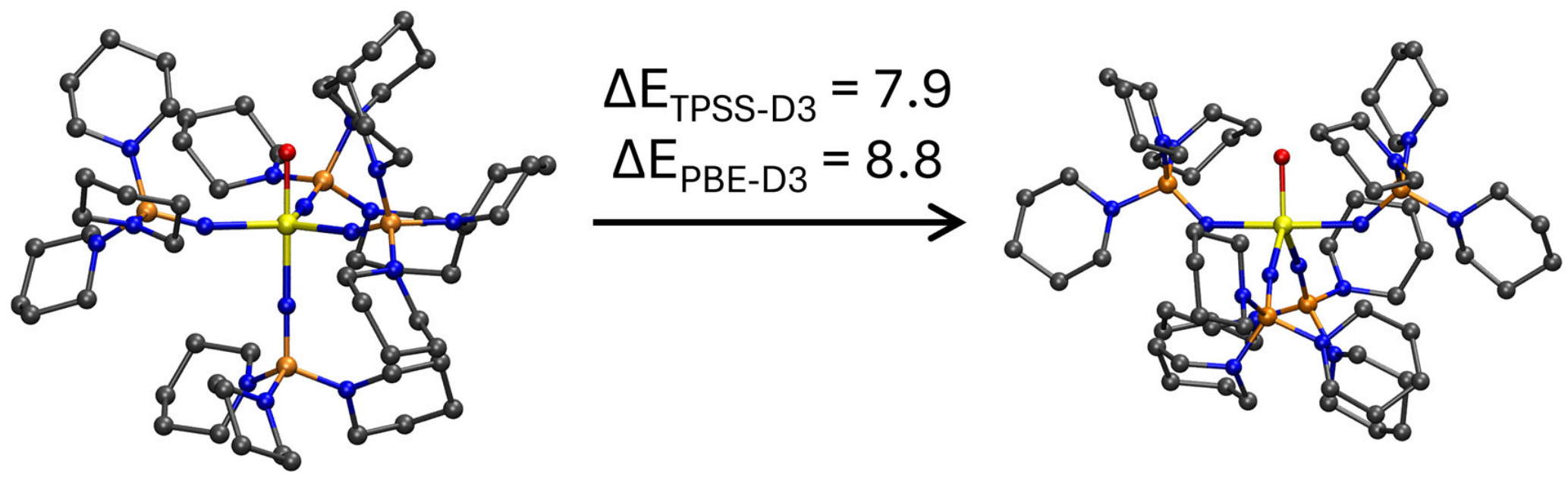

Figure S71: Ball and stick models of **UO-(NP^{pip})$_4$** (left) and one proposed intermediate, **UO-(NP^{pip})$_4$-int** (right). The relative electronic energies (in kcal/mol) are reported at the TPSS-D3(BJ)(COSMO,THF) and PBE-D3(BJ)(COSMO,THF) levels of theory.

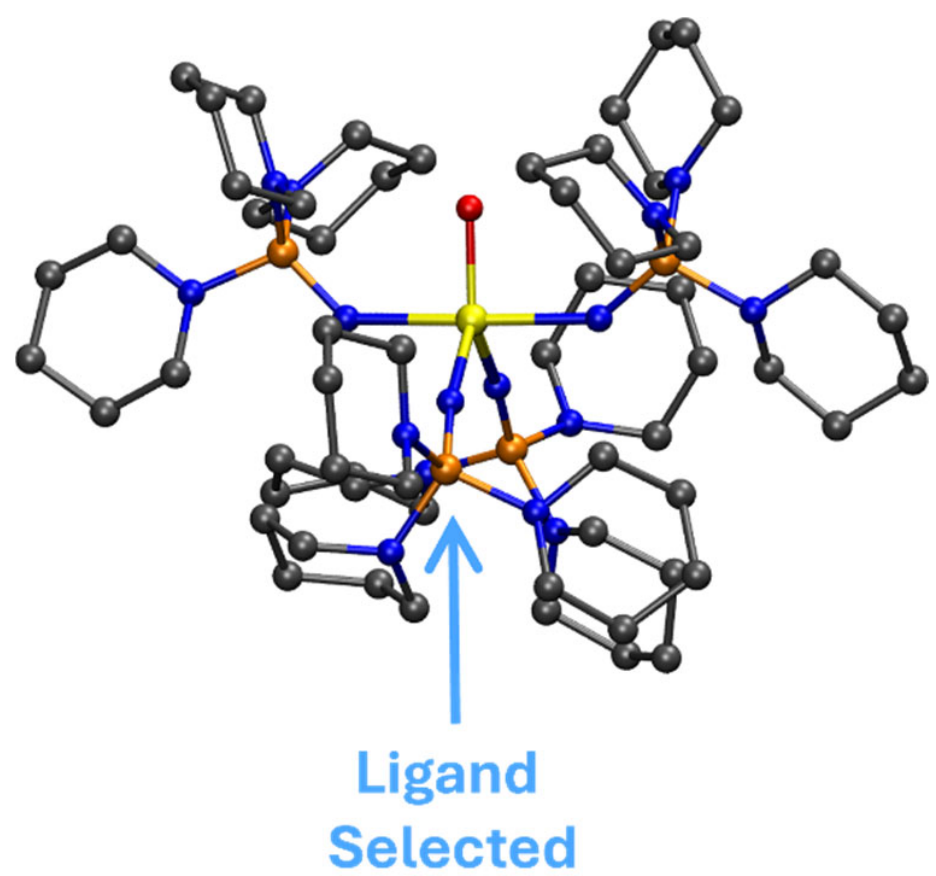

Figure S72: Ball and stick model of **UO-(NP^{pip})$_4$-int** highlighting the ligand used in the EDA calculations reported in **Table S25**, the ETS-NOCV calculations in

Table S26, and in **Figure S73**: NOCV deformation densities for the selected intermediate barrier structure of **UO-(NPpip)$_4$**. Energies are given in kcal/mol and in parentheses. An isosurface of 0.003 a.u. is used. Blue indicates regions of charge depletion, and yellow indicates regions of charge accumulation.

Table S25: Energy decomposition analysis results for **UO-(NP^{pip})$_4$** for the axial ligand and equatorial ligand. This is compared to the analysis for **UO-(NP^{pip})$_4$-int**; the selected ligand is highlighted in **Figure S72**. The results are reported at the TPSS-D3(BJ)/TZP level of theory on the TPSS COSMO geometry using THF as the solvent. The steric interaction is the sum of the Pauli repulsion and the electrostatic contribution. The percent electrostatic, orbitalic, and dispersion interactions are of the total attractive energy. Energies in kcal/mol.

	INT E	Pauli	Elect	Orb	Disp	%Elec	%Orb	%Disp	Steric
**Axial**	-189.2	285.7	-257.7	-184.7	-32.5	54.3	38.9	6.8	28.01
**Equatorial**	-168.9	192.1	-212.3	-123.0	-25.8	58.8	34.1	7.1	-20.12
**Intermediate**	-182.0	225.9	-227.5	-143.6	-36.7	55.8	35.2	9.0	-1.59

Table S26: ETS-NOCV analysis results for **UO-(NP^{pip})$_4$** for the axial ligand and equatorial ligand. This is compared to the analysis for one of the ligands in the intermediate structure **UO-(NP^{pip})$_4$-int** (proposed as representative of the barrier) shown on the right in **Figure S71**. NOCV pair orbital interaction energies are given for the strongest three interactions (all others are smaller than –6 kcal/mol). For each pair, the NOCV eigenvalue, $|v_e|$, which corresponds to the number of electrons transferred is given in parentheses. The total of all orbital interaction energies is also reported. Energies in kcal/mol. The results are reported at the TPSS-D3(BJ)/TZP level of theory on the TPSS COSMO geometry using THF as the solvent.

Axial	Equatorial	Intermediate
-66.1 (0.77)	-37.5 (0.63)	-38.9 (0.72)
-33.5 (0.71)	-22.4 (0.60)	-25.1 (0.64)
-33.5 (0.71)	-24.1 (0.51)	-34.5 (0.58)
-182.6	*-120.6*	*-143.0*

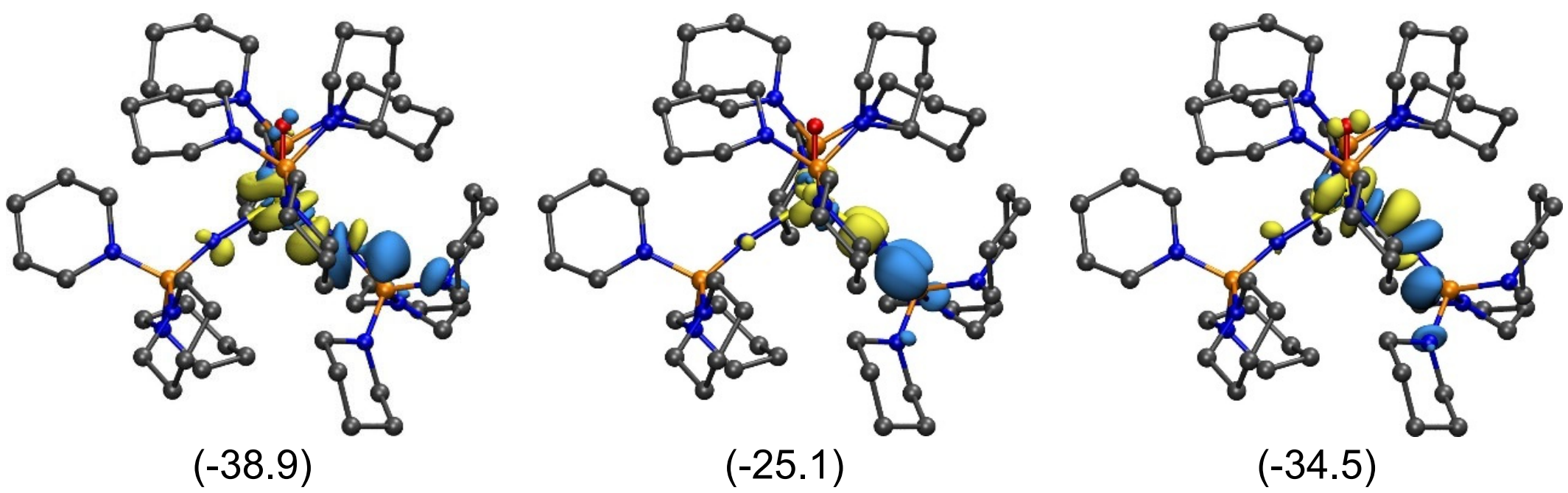

Figure S73: NOCV deformation densities for the selected intermediate barrier structure of **UO-(NP^{pip})$_4$.** Energies are given in kcal/mol and in parentheses. An isosurface of 0.003 a.u. is used. Blue indicates regions of charge depletion, and yellow indicates regions of charge accumulation.

Table S27: The G-parameters, G(Complex), computed from the TPSS optimized structures, using the Solid-G program.

	G-parameter
**UO-(NP^{pyrr})$_4$**	89.22
**UO-(NP^{pip})$_4$**	89.45
**UO-(NPC^1)$_4$**	88.87
**UO-(NPC$^{1\text{-}pip}$)$_4$**	91.11

References

(1) Johnson, S. A.; Kiernicki, J. J.; Fanwick, P. E.; Bart, S. C. New Benzylpotassium Reagents and Their Utility for the Synthesis of Homoleptic Uranium(IV) Benzyl Derivatives. *Organometallics* **2015**, *34* (12), 2889.

(2) Monreal, M. J.; Thomson, R. K.; Cantat, T.; Travia, N. E.; Scott, B. L.; Kiplinger, J. L. UI_4(1,4-dioxane)$_2$, [UCl_4(1,4-dioxane)]$_2$, and UI_3(1,4-dioxane)$_{1.5}$: Stable and Versatile Starting Materials for Low- and High-Valent Uranium Chemistry. *Organometallics* **2011**, *30* (7), 2031.

(3) Carpino, L. A.; Padykula, R. E.; Barr, D. E.; Hall, F. H.; Krause, J. G.; Dufresne, R. F.; Thoman, C. J. Synthesis, characterization, and thermolysis of 7-amino-7-azabenzonorbornadienes. *J. Org. Chem.* **2002**, *53* (11), 2565.

(4) Rice, N. T.; McCabe, K.; Bacsa, J.; Maron, L.; La Pierre, H. S. Two-Electron Oxidative Atom Transfer at a Homoleptic, Tetravalent Uranium Complex. *J. Am. Chem. Soc.* **2020**, *142* (16), 7368.

(5) Niklas, J. E.; Studvick, C. M.; Bacsa, J.; Popov, I. A.; La Pierre, H. S. Ligand Control of Oxidation and Crystallographic Disorder in the Isolation of Hexavalent Uranium Mono-Oxo Complexes. *Inorg. Chem.* **2023**, *62* (5), 2304.

(6) Willcott, M. R. MestRe Nova. *J. Am. Chem. Soc.* **2009**, *131* (36), 13180.

(7) Bruker, *SAINT V8.40B*; Bruker AXS Inc., Madison, Wisconsin, USA.

(8) Krause, L.; Herbst-Irmer, R.; Sheldrick, G. M.; Stalke, D. Comparison of silver and molybdenum microfocus X-ray sources for single-crystal structure determination. *J. Appl. Crystallogr.* **2015**, *48* (1), 3.

(9) Sheldrick, G. SHELXT - Integrated space-group and crystal-structure determination. *Acta Crystallogr., Sect. A* **2015**, *71* (1), 3.

(10) Sheldrick, G. Crystal structure refinement with SHELXL. *Acta Crystallogr., Sect. C:Struct. Chem.* **2015**, *71* (1), 3.

(11) Dolomanov, O. V.; Bourhis, L. J.; Gildea, R. J.; Howard, J. A. K.; Puschmann, H. OLEX2: a complete structure solution, refinement and analysis program. *J. Appl. Crystallogr.* **2009**, *42* (2), 339.

(12) Kratzert, D. *FinalCif V125*, https://dkratzert.de/finalcif.html.

(13) Macrae, C. F.; Sovago, I.; Cottrell, S. J.; Galek, P. T. A.; McCabe, P.; Pidcock, E.; Platings, M.; Shields, G. P.; Stevens, J. S.; Towler, M.; Wood, P. A. Mercury 4.0: from visualization to analysis, design and prediction. *J. Appl. Crystallogr.* **2020**, *53* (1), 226.

(14) Cavanagh, J.; Fairbrother, W. J.; Palmer, A. G.; Rance, M.; Skelton, N. J. CHAPTER 3 - EXPERIMENTAL ASPECTS OF NMR SPECTROSCOPY. *Protein NMR Spectroscopy (Second Edition)*; Academic Press, **2007**; pp 114.

(15) Shanan-Atidi, H.; Bar-Eli, K. H. A convenient method for obtaining free energies of activation by the coalescence temperature of an unequal doublet. *J. Phys. Chem.* **1970**, *74* (4), 961.

(16) Egan, W. M. I. The barrier to pyramidal inversion in phospholes II. Nucleophilic displacements at phosphorus in 3-methyl-1-phenylphospholanes. Ph.D. Thesis, Princeton University, **1971**.

(17) OriginPro, Version 2024, OriginLab Corporation, Northhampton, MA, USA.

(18) Jeener, J.; Meier, B. H.; Bachmann, P.; Ernst, R. R. Investigation of exchange processes by two-dimensional NMR spectroscopy. *J. Chem. Phys.* **1979**, *71* (11), 4546.

(19) Hayton, T. W.; Autschbach, J. Using NMR Spectroscopy to Evaluate Metal–Ligand Bond Covalency for the f Elements. *Acc. Chem. Res.* **2025**, *58* (3), 488.

(20) Vícha, J.; Novotný, J.; Komorovsky, S.; Straka, M.; Kaupp, M.; Marek, R. Relativistic Heavy-Neighbor-Atom Effects on NMR Shifts: Concepts and Trends Across the Periodic Table. *Chem. Rev.* **2020**, *120* (15), 7065.

(21) Rusakova, I. L.; Rusakov, Y. Y. Modern Quantum Chemistry Methodology for Predicting ^{31}P Nuclear Magnetic Resonance Chemical Shifts. In *Int. J. Mol. Sci.*, 2026; Vol. 27, p 704.

(22) Latypov, S. K.; Polyancev, F. M.; Yakhvarov, D. G.; Sinyashin, O. G. Quantum chemical calculations of ^{31}P NMR chemical shifts: scopes and limitations. *Phys. Chem. Chem. Phys.* **2015**, *17* (10), 6976.

(23) Sarotti, A. M.; Pellegrinet, S. C. A Multi-standard Approach for GIAO ^{13}C NMR Calculations. *J. Org. Chem.* **2009**, *74* (19), 7254.

(24) Sarotti, A. M.; Pellegrinet, S. C. Application of the Multi-standard Methodology for Calculating ^{1}H NMR Chemical Shifts. *J. Org. Chem.* **2012**, *77* (14), 6059.

(25) Payard, P.-A.; Perego, L. A.; Grimaud, L.; Ciofini, I. A DFT Protocol for the Prediction of ^{31}P NMR Chemical Shifts of Phosphine Ligands in First-Row Transition-Metal Complexes. *Organometallics* **2020**, *39* (17), 3121.

(26) Jameson, C. J.; De Dios, A.; Keith Jameson, A. Absolute shielding scale for ^{31}P from gas-phase NMR studies. *Chem. Phys. Lett.* **1990**, *167* (6), 575.
